# Increase in wild animal consumption across Central Africa

**DOI:** 10.1038/s41586-026-10422-w

**Published:** 2026-04-29

**Authors:** Mattia Bessone, Daniel J. Ingram, Katharine Abernethy, Sylvanus Abua, Sophie Allebone-Webb, Daniela Antonacci, Riyong Kim, Stephanie Brittain, Daniel Cornelis, Diane Detoeuf, Charles A. Emogor, Julia E. Fa, Steffen Foerster, Davy Fonteyn, Maria Grande Vega, Chloe Hodgkinson, Amy Ickowitz, Cédric Thibaut Kamogne Tagne, Della Kemalasari, Noëlle Kümpel, Simon Lhoest, Germain Mavah, Rodrigue Guy Mouanda Niamba, Donald Midoko Iponga, Eleanor J. Milner-Gulland, Jonas Muhindo, Théodore Munyuli, Robert Nasi, Steeve Ngama, Jonas Nyumu, Justin Ombeni, John R. Poulsen, Dominic Rowland, Yahya Sampurna, François Sandrin, Malcolm Starkey, Caleb Tata, Julius C. Tieguhong, Nathalie van Vliet, Philippe Vigneron, Robin C. Whytock, Michelle Wieland, David Wilkie, Jasmin Willis, Juliet Wright, Lauren Coad

**Affiliations:** 1 Center for International Forestry Research, World Agroforestry (CIFOR-ICRAF), Bogor, Indonesia; 2 Department for the Ecology of Animal Societies, Max Planck Institute of Animal Behavior, Konstanz, Germany; 3 Centre for the Advanced Study of Collective Behaviour, University of Konstanz, Konstanz, Germany; 4 Durrell Institute of Conservation and Ecology (DICE), School of Natural Sciences, University of Kent, Canterbury, UK; 5 Faculty of Natural Sciences, University of Stirling, Stirling, UK; 6 Institute for Tropical Ecology Research (IRET), CENAREST, Libreville, Gabon; 7 Zoological Society of London, London, UK; 8 Centre for Environmental Policy & Department of Life Sciences, Imperial College London, Ascot, UK; 9 Wildlife Conservation Society, New York, NY USA; 10 Centre for Forest and Landscape, University of Copenhagen, Frederiksberg, Denmark; 11 Interdisciplinary Centre for Conservation Science (ICCS), Department of Biology, University of Oxford, Oxford, UK; 12 Forêts et Sociétés, Université de Montpellier, CIRAD, Montpellier, France; 13 Department of Zoology, University of Cambridge, Cambridge, UK; 14 Department of Natural Sciences, Manchester Metropolitan University, Manchester, UK; 15 Natural Sciences and Environment Hub, University of Gibraltar, Campus Europa Point, Gibraltar; 16 Department of Evolutionary Anthropology, Duke University, Durham, NC USA; 17 Research Group SILVANET, College of Forestry and Natural Environment, Universidad Politécnica de Madrid, Madrid, Spain; 18 Asociación Ecotono, Madrid, Spain; 19 Fauna & Flora, Cambridge, UK; 20 University College London, London, UK; 21 Collective Action to Save the Environment (CASE), Yaoundé, Cameroon; 22 BAY-SUP, The Higher Institute of Environmental Sciences, Yaoundé, Cameroon; 23 BirdLife International, Cambridge, UK; 24 Gembloux Agro-Bio Tech, Université de Liège, Gembloux, Belgium; 25 Oxford Martin School, University of Oxford, Oxford, UK; 26 Solutions for Wildlife (SO WILD), Kisangani, Democratic Republic of the Congo; 27 Department of Nutrition and Dietetics, Institut Supérieur de Techniques Médicales ISTM, Bukavu, Democratic Republic of the Congo; 28 Laboratory of Entomology, Centre de Recherche en Sciences Naturelles CRSN-LWIRO, Bukavu, Democratic Republic of the Congo; 29 Wildlife and Sustainable Development Research Program, IRAF-CENAREST, Libreville, Gabon; 30 University of Kisangani (UNIKIS), Kisangani, Democratic Republic of the Congo; 31 Laboratory of Functional and Applied Entomology, LENAF, Institut Facultaire des Sciences Agronomiques IFA, Kisangani, Democratic Republic of the Congo; 32 Nicholas School of the Environment, Duke University, Durham, NC USA; 33 The Nature Conservancy, Boulder, CO USA; 34 The Biodiversity Consultancy, Cambridge, UK; 35 Department of Geography, University of Cambridge, Cambridge, UK; 36 Forests, Resources and People (FOREP), Limbe, Cameroon; 37 African Natural Resources Management and Investment Centre, African Development Bank, Abidjan, Ivory Coast; 38 Okala, Stirling, UK

**Keywords:** Sustainability, Conservation biology, Developing world, Conservation biology

## Abstract

While human activities are driving widespread declines in wildlife populations^1,2^, in Central Africa, the meat of wild animals, or wild meat, represents a major component of the diets of millions of people^3^. To halt faunal degradation while ensuring sustainable use of wildlife, it is crucial to understand the scale and drivers of wild meat consumption. Here, using data from over 12,000 households from 252 locations in Central Africa, we show that wild meat is a fundamental component of the diets of rural populations, accounting for 20% of the recommended daily protein intake, compared with 13% and 6% for those living in towns and cities. We estimate that the total annual biomass of wild meat consumed in Central Africa increased from 0.73 million to 1.10 million tonnes between 2000 and 2022, with increasing demand from towns and cities. To ensure that wild meat is available to rural communities, in accordance with the Sustainable Development Goals^4^ and the Kunming–Montreal Global Biodiversity Framework^5^, reducing wild meat consumption in urban metropolises is key. While our results are based on the most comprehensive dataset available, the geographical coverage is incomplete and the dataset represents a minimal fraction of the entire population of Central Africa. Targeted studies are needed to validate our model and assess critical areas of intervention.

## Abstract (web summary)

Wild meat is a fundamental component of the diets of rural Central African populations, accounting for 20% of the recommended daily protein intake, compared with 13% and 6% for those living in towns and cities.

## Main

The growth of the human population from 1.6 to 8 billion people over the past two centuries has greatly increased the global demand for food^6^, catalysing considerable changes in food production and distribution systems. While globalized food systems are among the main drivers of climate change^7^ and biodiversity loss^8^, they also provide the growing human population with access to food, including meat^9^.

In contrast to the tropical forest regions of Latin America and Asia^10^, in Central Africa, food systems are still largely based on small enterprises and family farms^11^ or on hunter-gatherer foraging lifestyles^12^, and the meat of wild animals, or wild meat, remains a primary source of food for millions of people^3^. For millennia, when human population densities were far lower than present day and hunting was a matter of subsistence, hunting may have been sustainable for most wild species^13^. However, in the past century, human populations in Central Africa have grown from 25 to 140 million, greatly increasing the demand for both food and income^14^. Nowadays, human consumption of wildlife is a threat to 31% of all mammals, birds, reptiles and amphibians currently threatened with extinction in the region^2^.

In much of remote rural Central Africa, marine fish and meat from livestock are in short supply and expensive due to poor national transport infrastructure^15^ and fiscal and administrative barriers to local business development^16,17^. Moreover, livestock diseases^18^ and a lack of forage^19^ make livestock rearing challenging. Consequently, rural communities often consume wild meat and local freshwater fish as their main animal source foods, which provide most of the proteins^20,21^ and micronutrients^22^ necessary to fulfil nutrient requirements.

However, 51% of Central Africans now live in urban areas^23^, where direct access to wild food resources can be scarce but modern domestic food systems are often still underdeveloped^24^. The trade of wild meat into these cities is mostly unregulated^25^ and has therefore become a major source of income for both rural and urban individuals^26^. Established major cities in Central Africa have developed peri-urban agriculture and viable international import infrastructure. However, imported meats are often from highly intensive production systems with high environmental costs, and are considered to be unhealthy by some consumers^27^. Current international trade regulations allowing the import of cheap foreign meat has also disincentivized the creation of large-scale national domestic meat production^16^. The consumption of wild meat is still deeply embedded in the culture of many Central African urban populations^3^ and purchased for reasons associated with taste^28^, health, cultural celebration or as a status symbol^27,29^. These purchases, although infrequent per capita, are sufficient to drive a thriving supply chain^30^.

The consumption of wild meat is therefore a major component of Central Africa’s socioeconomic fabric, and ensuring that any use of wildlife is sustainable is key to achieving the United Nations Sustainable Development Goals (SDGs) by 2030^4^. Wild meat consumption makes important contributions to human nutrition (goal 2: zero hunger) and health (goal 3: good health and well-being), particularly in rural areas, but achieving responsible consumption (goal 12) by urban consumers, who typically eat wild meat for reasons other than subsistence, is crucial for ensuring sustainability^4^. Managing the trade of wildlife for food has potential ramifications for the SDGs, as the wild meat sector provides informal employment to many people (goal 8: decent work and economic growth), including women (goal 5: gender equality). If not properly managed, overexploitation poses a threat to biodiversity (goal 15: life on land), with potential consequences on ecosystem services and functioning (goal 13: climate action)^4^.

While numerous site-level studies have provided key insights into wild meat consumption, taken in isolation, they are unable to provide an overview at the scale required for national and regional policy making and planning. This study collates and analyses all available site-level data to provide an evidence base to inform national and regional policy discussions, providing a quantitative spatial and temporal analysis of wild meat consumption in Central Africa. Specifically, we investigate: (1) the ecological, economic and sociocultural factors associated with wild meat consumption; (2) how consumption rates vary geographically within the region; (3) how consumption rates have changed over time.

## Creating a regional evidence base

**Fig. 1:**
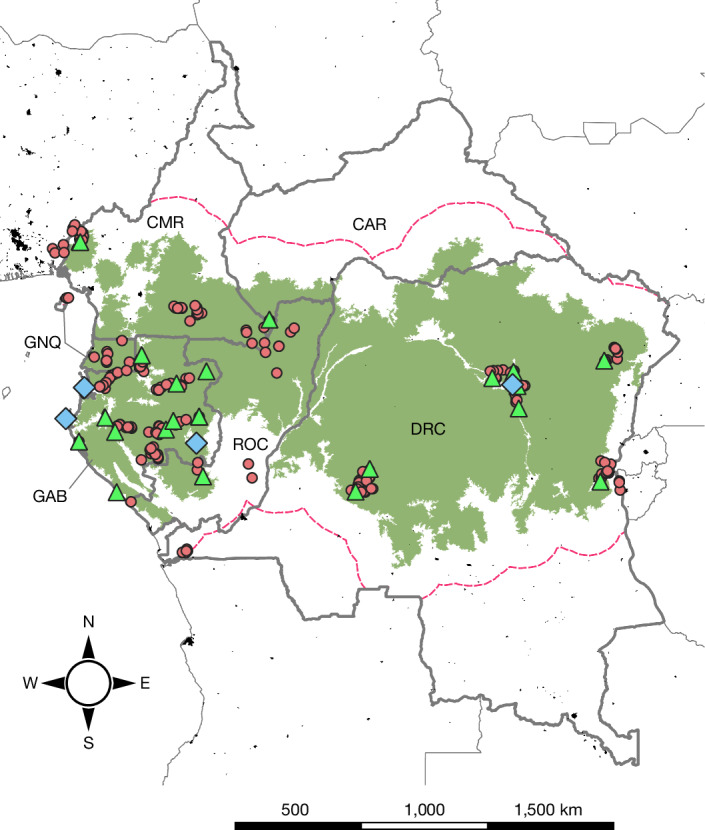
Geographical distribution of the 252 locations included in the analysis. Villages (red dots), towns (green triangles) and cities (blue diamonds), monitored between 2000 and 2022 in Cameroon (CMR), Central African Republic (CAR), Democratic Republic of the Congo (DRC), Equatorial Guinea (GNQ), Gabon (GAB) and Republic of the Congo (ROC). Two studies were conducted in the Nigerian (NGA) part of the Cross River–Korup–Takamanda transnational landscape, representing the same forest system, located less than 70 km from the border with Cameroon. The green areas represent patches of continuous forest (>5,000 km^2^)^68^ and the black areas depict large urban centres^37^. The dashed purple line represents the region used to predict regional consumption rates and consumed biomass. This area represents a buffer (radius, 140 km) around the major patches of the Central African Forest region, encompassing all of the surveyed locations and including areas of the forest-savannah transition (Supplementary Fig. 1). Credit: country outlines, https://geoportal.icpac.net/ under an Open Database License ODbL 1.0; the map was created using QGis (v.3.22.1)^69^.

**Extended Data Fig. 1:**
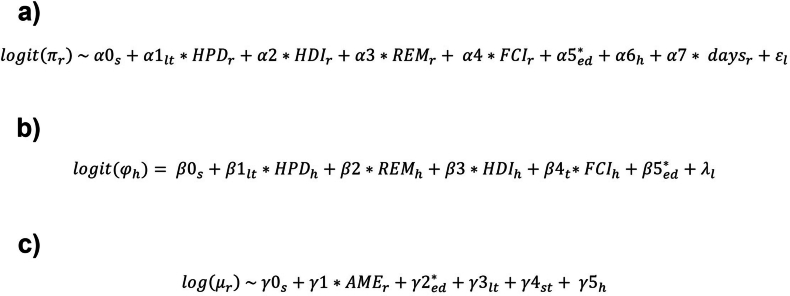
Linear models for the estimation of wild meat a) consumption probability π; b) frequency of consumption φ; b) consumed quantity per AME μ. a) α0 is a varying intercept by study *s* (number of levels “n” = 30); α1 is the parameter of the effect of *HPD*, varying by location type *lt* (interaction between *HPD* and *LT* [n = 3]); α2, α3 and α4 are covariate-specific slopes for remoteness *REM*, human development index *HDI* and forest condition index *FCI* respectively; α5 is categorical parameter for education level *ED* ([n = 3]); α6 is a random factor varying by household *h*; α7 is the parameter defining the increment in $\pi$ as a function of the recall duration *days*; $\varepsilon$ is the spatial autocorrelation term between each location *l*. b) ${\beta }$0 is the intercept varying by study *s*; ${\beta }$1 is the parameter of the effect of human population density *HPD*, varying by location type *lt*; ${\beta }$2, ${\beta }$3 and ${\beta }$4 are covariate-specific slopes for *REM*, *HDI* and *FCI* respectively and ${\beta }$5 is categorical parameter for education level *ED* ([n = 3]); *λ* is the spatial autocorrelation term between each location *l*. c) *γ*0 is the intercept varying by study *s*; *γ*1 is the slope for the number of AME participating in a recall event; *γ*2 is categorical parameter for education level *ED* ([n = 3]), *γ*3 and *γ*4 are categorical parameters for location type *lt* and study type *st*; and *γ*5 is a random factor varying by household *h* | * in the models evaluating the interaction between *ED* and *LT* [n = 7], this parameter is defined as ${x}_{{ed},{lt}}$ (Supplementary Results – Supplementary Table 5).

We collated consumption data from 30 published and unpublished wild meat consumption studies, conducted between 2000 and 2022 and covering 252 locations in seven Central African countries (approximately 4 × 10^6^ km^2^; Fig. 1). These studies represented both rural and urban areas, and each location was defined as village (*n* = 224), town (*n* = 24) or city (*n* = 4) by data providers, considering subregional differences in settlement population size. Collectively, our database includes data from 12,453 individual households and 163,896 recall events, defined as occasions when households were asked about wild meat consumption in a given period between 1 to 365 days. We considered three different types of data, characterized by increasing levels of information with respect to wild meat consumption (Extended Data Table 1), described in detail in the ‘Data preparation’ section of the Methods:

1. Probability of consumption: whether a household consumed meat (1) or not (0) (that is, consumption events) over a certain period (Extended Data Fig. 1a).
2. Frequency of consumption: how often wild meat was consumed by a household over a certain period (that is, the number of consumption events/the duration (in days) of the recall) (Extended Data Fig. 1b).
3. Quantity consumed: the quantity of undressed wild meat consumed by the household over a certain period. To account for lower energetic requirements of women and children, we standardized our estimates by using the adult male equivalent (AME) transformation^31^, therefore referring to the quantity of wild meat consumed per day per AME (Extended Data Fig. 1c).

## Correlates of wild meat consumption

On the basis of existing literature, we evaluated a set of potential predictors of wild meat consumption probability, frequency and quantity (Extended Data Table 2). Motivations, research questions and hypotheses specific to each fixed and random factor included in the model are described in the Methods.

**Fig. 2:**
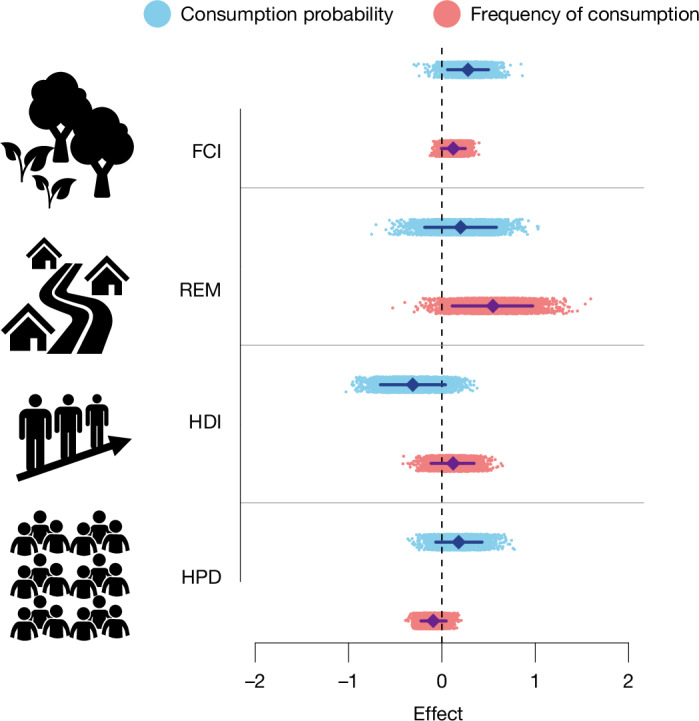
Estimated effect of the predictors of wild meat consumption and frequency of consumption. The estimated effects (where 0 is no effect) of the predictors of wild meat consumption probability (blue) and frequency of consumption (pink) are shown. FCI, forest condition index; REM, remoteness; HDI, human development index; HPD, human population density. The coloured clouds of dots show the posterior distribution of the effect estimated by the model (*n* = 4,000 posterior draws). The coloured diamonds show the mean of the posterior distribution. The solid bars show the 95% highest posterior density intervals. Icons are from https://www.svgrepo.com/ under a Creative Commons CC0 1.0 Licence.

**Fig. 3:**
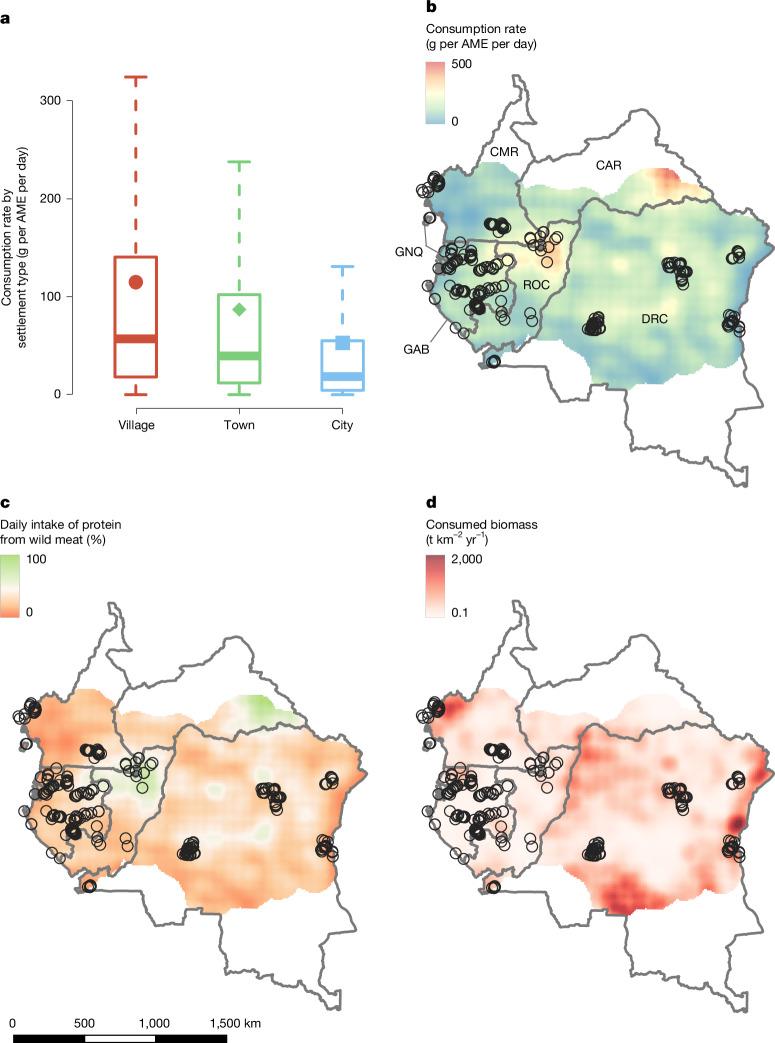
Model prediction of wild meat consumption in the Central African forest region. **a**, The estimated daily quantity (grams undressed wild meat per AME per day) consumed in villages (<10,000 inhabitants, red), towns (>10,000 and <100,000, green) and cities (>100,000, blue) obtained from* n* = 4,000 posterior draws. The box limits show the interquartile range and the whiskers show 1.5× the interquartile range (outliers are not shown for clarity). The solid horizontal lines show the median of the posterior distribution. The coloured dots show the mean of the posterior distribution.** b**, Geographical variation in estimated consumption rates (grams undressed wild meat per AME per day) in 2022.** c**, Estimated wild meat contribution to the recommended daily protein intake.** d**, Geographical variation in estimated total consumed biomass in 2022. The black circles show the surveyed locations. A detailed discussion of these maps is provided in the Supplementary Discussion. Country outlines are from https://geoportal.icpac.net/ under an Open Database License ODbL 1.0; maps were created using QGis (v.3.22.1)^69^.

**Extended Data Fig. 2:**
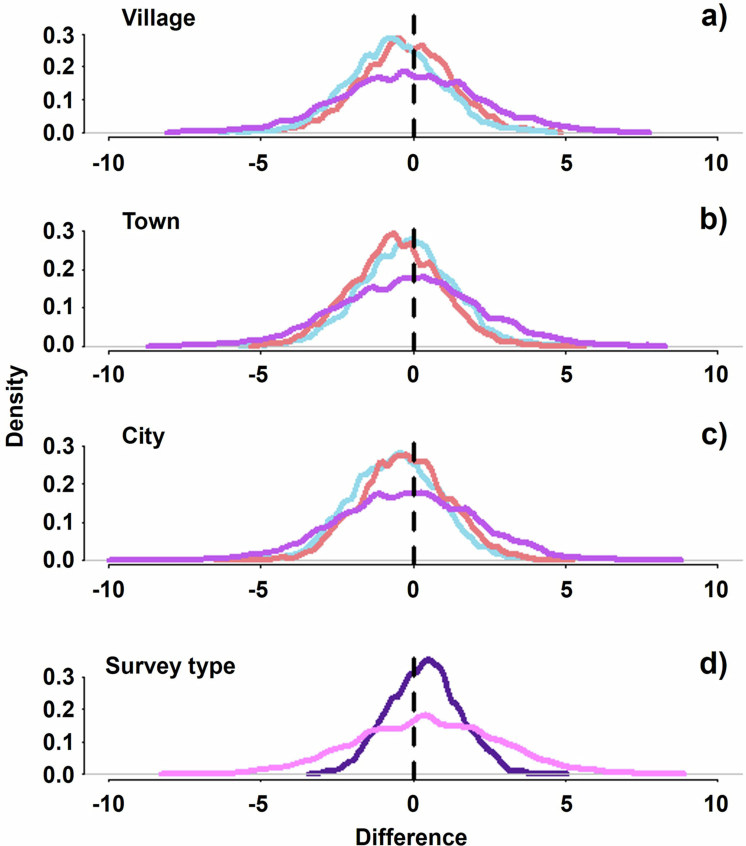
Effect of education level in different settlement types (panels a,b,c) and survey type (panel d) on wild meat consumption probability (blue), frequency of consumption (days/week; pink) and quantity consumed (g/AME/day; purple). Effect on wild meat consumption of 1) education level in villages (<10,000 inhabitants) (a), towns (>10,000 and <100,000) (b), cities (>100,000) (c); 2) survey type (d); *Coloured shapes*: posterior distribution (n = 4,000 posterior draws) of the contrasts (i.e., difference) between the parameters: 1) education level =<primary vs. education level >= secondary for each settlement type (panels a, b, c); 2) short (=<72 h; light purple) and long recall periods (> 72 h; dark purple) vs. cooking pot studies; *Dashed lines*: denote a difference of 0 (i.e., no difference). Note that all plots show posterior distributions overlapping 0 (or mostly overlapping 0), indicating a non-significant difference between parameters (e.g., education level “=<primary” and “>= secondary” or “short recall studies” vs. “cooking pot studies).

The first finding of our analysis was the absence of survey-inherent bias (Extended Data Fig. 2), showing that observed consumption rates were not dependent on the survey method adopted, therefore confirming the comparability of the studies included. Turning to our analysis of possible factors associated with wild meat consumption, we found that forest condition was associated with both the increased probability and frequency of consumption, while remoteness was correlated with a higher frequency of consumption only (Fig. 2). In other words, residents of remote rural communities with access to forest where wildlife is probably abundant and wild meat is largely available and cheap were more likely to consume wild meat, and more often, compared with those from more urbanized areas^32^. Although the estimated effect was inconclusive, our results suggested that wild meat consumption increased with lower values of human development and human population density (Fig. 2 and Supplementary Fig. 8). As a result, wild meat was consumed more often in villages than in towns, with residents of cities consuming the least wild meat (Fig. 3a). We expected that education level and wealth would influence wild meat consumption differently depending on the type of settlement (that is, village, town, city). However, education level did not appear to affect wild meat consumption when assessing the interaction between education level and type of settlement (Extended Data Fig. 2 and Supplementary Table 5), or when households from all settlement types were aggregated (Extended Data Table 3). Finally, the quantity of wild meat consumed in a day per AME was lower when more people were participating in the meal, decreasing by 2.96 g per AME per day (95% confidence interval (CI) = 2.50–3.93) for each additional AME. It could be that households can only afford to buy a certain quantity of wild meat, which must then be shared between more people when households are larger, consistent with previous studies which have shown that larger households can be more likely to be food insecure^33^.

## Wild meat consumption in Central Africa

We estimated a median regional consumption of 50 g per AME per day (mean = 106; s.d. = 169; 95% CI = 10–548) of undressed wild meat. When we restricted our predictions to the Central African forest region, an area of approximately 3 × 10^6^ km^2^ encompassing all the locations included in our analyses while excluding the Sahel regions of Cameroon and Central African Republic and southern Democratic Republic of the Congo (Fig. 2), we obtained similar consumption rates of 51 g per AME per day (mean = 107; s.d. = 171; 95% CI = 10–556), suggesting that wild meat consumption is widespread across the region. We note that the estimated consumption rate accounts for days when wild meat is not consumed and therefore represents the average consumption across survey days.

The World Health Organization recommends a minimum protein intake of 56 g per day for an adult male individual^34^. Although wild meat provides 29.4 g of dry protein per 100 g^35^, only 70% of undressed meat can be consumed and therefore provides protein (that is, dressed meat)^36^. Accordingly, our results indicate that wild animals might account for 18% of the recommended daily protein intake of the region’s population (if we consider the median estimated value: 10 g). However, in many rural areas of the Central African forest region, wild meat contribution is crucial to satisfying daily protein requirements (Fig. 3c).

**Extended Data Fig. 3:**
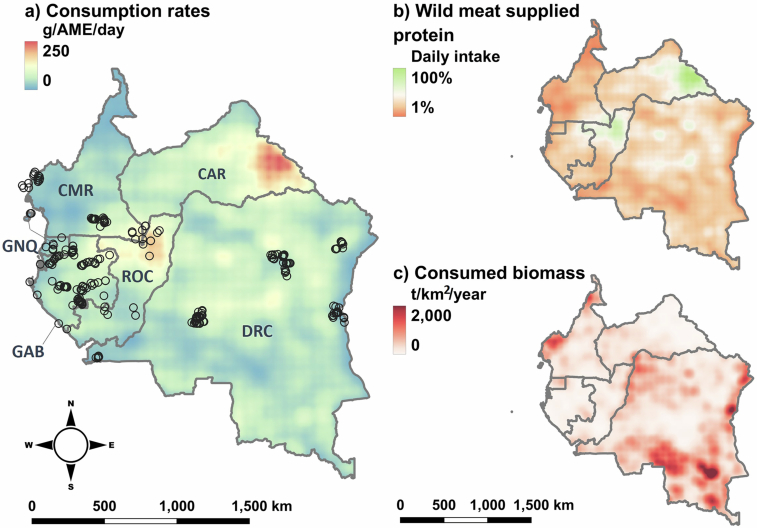
Model prediction of wild meat consumption in the entire Central African region (grams undressed wild meat/AME/day). a) Geographical variation in estimated consumption rates (grams undressed wild meat/AME/day) in 2022; b) Estimated wild meat contribution to the recommended daily protein intake; c) Geographical variation in estimated total consumed biomass in 2022. *Black circles*: surveyed locations. A detailed discussion of these maps is provided in the Supplementary Discussion. Credit: country outlines, https://geoportal.icpac.net/ under an Open Database License ODbL 1.0; map created with QGis 3.22.1^69^.

The human population in Central Africa grew from 72 to over 130 million people in 22 years (2000–2022)^23^ and the total mass of undressed wild meat consumed in the region increased accordingly: from an annual median of 0.99 × 10^6^ t between 2000 and 2010 (mean = 1.45 × 10^6^; s.d. = 1.49 × 10^6^; 95% CI = 0.16 × 10^6^–5.43 × 10^6^), to 1.35 × 10^6^ t per year (mean = 1.96 × 10^6^; s.d. = 2.00 × 10^6^; 95% CI = 0.22 × 10^6^–7.44 × 10^6^) between 2011 and 2021. In our most recent estimate, we calculated that as many as 1.62 × 10^6^ t (mean = 2.37 × 10^6^; s.d. = 2.42 × 10^6^; 95% CI = 0.27 × 10^6^–8.77 × 10^6^) of wild meat were consumed in the region in 2022 (Extended Data Fig. 3). Of those, approximately 68% (median = 1.10 × 10^6^ t per year; mean = 1.61 × 10^6^; s.d. = 3.49 × 10^6^) were consumed in the Central African forest region in 2022 (Fig. 3d), decreasing from 71% between 2011 and 2021 (median = 0.96 × 10^6^ t per year; mean = 1.38 × 10^6^; s.d. = 3.02 × 10^6^) and 74% between 2000 and 2010 (median = 0.73 × 10^6^ t per year; mean = 1.06 × 10^6^; s.d. = 2.34 × 10^6^). The increasing proportion of wild meat consumed in savannah-dominated areas (from 27% in 2000–2010 to 32% in 2022) seems to suggest a growing wild meat trade towards populated areas far from the Central African forest region, for example, southern Democratic Republic of the Congo (Fig. 3c).

**Extended Data Fig. 4:**
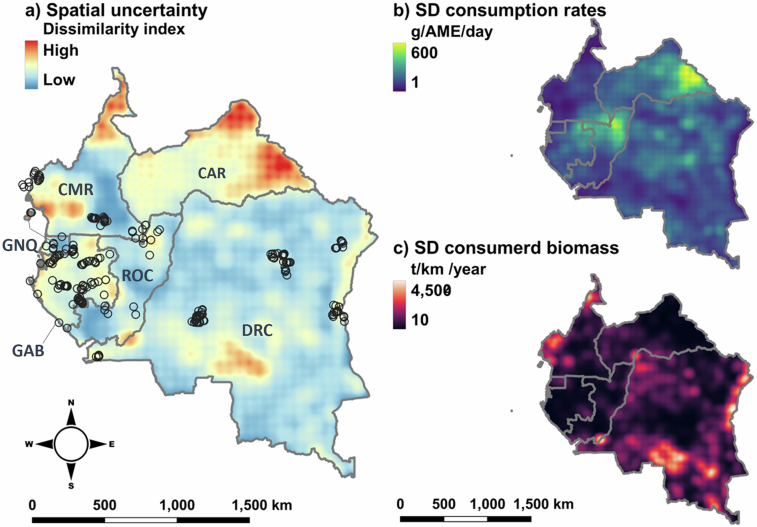
Uncertainty of estimated wild meat consumption in Central Africa. a) Spatial uncertainty based on the difference in characteristics (i.e., continuous variables included in the analysis) between data and predictions [*Black circles*: surveyed locations]; b) Geographical variation in the uncertainty (standard deviation) of estimated consumption rates; c) Geographical variation in the uncertainty (standard deviation) of estimated consumed biomass. *Black circles*: surveyed locations. A detailed discussion of these maps is provided in the Supplementary Discussion. Credit: country outlines, https://geoportal.icpac.net/ under an Open Database License ODbL 1.0; maps created with QGis 3.22.1^69^.

**Extended Data Fig. 5:**
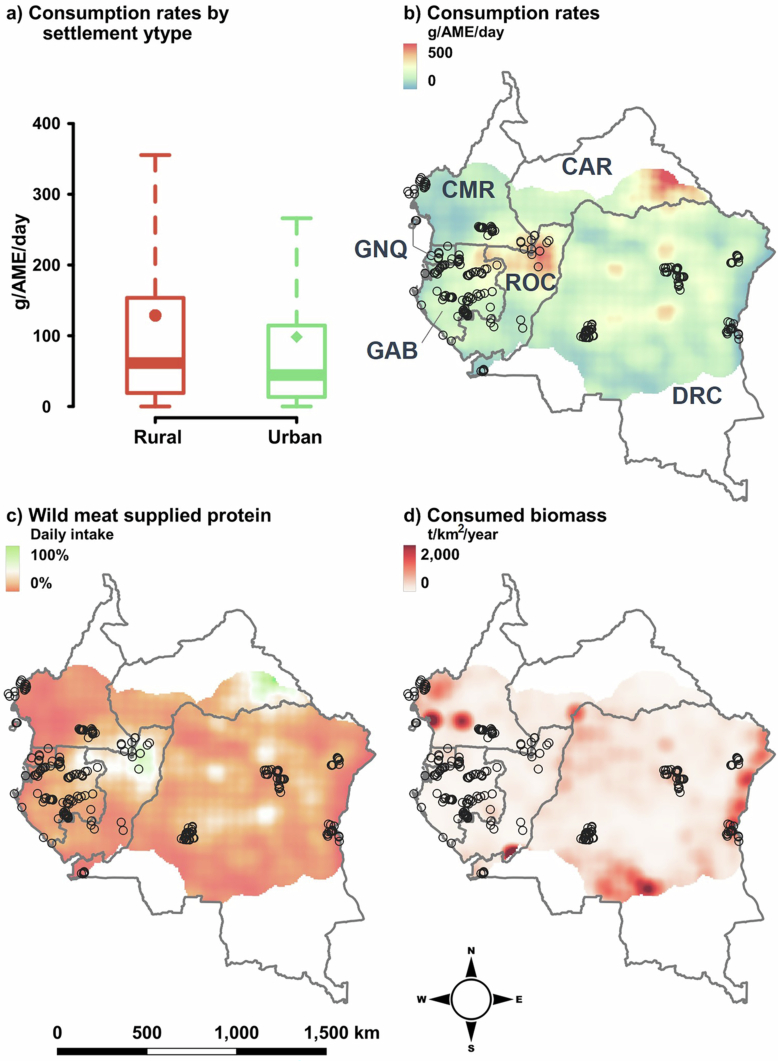
Model prediction of wild meat consumption in the entire Central African forest region (grams undressed wild meat/AME/day) predicting for only two settlement types (rural vs urban^37^). a) Estimated daily quantity consumed in rural (<10,000 inhabitants – red) and urban (>10,000 – green) areas obtained from n = 4,000 posterior draws [*Box*: interquartile range. *Whiskers*: 1.5 times the interquartile range (outliers not shown for clarity). *Solid horizontal lines*: median of the posterior distribution. *Coloured dots*: mean of the posterior distribution]; b) Geographical variation in estimated consumption rates (grams undressed wild meat/AME/day) in 2022; c) Estimated wild meat contribution to the recommended daily protein intake; d) Geographical variation in estimated total consumed biomass in 2022. *Black circles*: surveyed locations. A detailed discussion of these maps is provided in the Supplementary Discussion. Credit: country outlines, https://geoportal.icpac.net/ under an Open Database License ODbL 1.0; maps created with QGis 3.22.1^69^.

Although our estimates are based on the most comprehensive evidence available, we highlight that (1) our sample does not cover the entire region and that our predictions include areas outside those used in our analyses (Fig. 1 and Extended Data Fig. 4a); and (2) areas of higher consumption rates are associated with the high uncertainty of predicted values due to the use of positive-bounded data (Extended Data Fig. 4c). We therefore stress that our estimates must be considered along with the uncertainty of the estimated values (Extended Data Fig. 4). Specifically, we advise caution when using absolute values estimated by the model and instead suggest focusing on the relative importance of the areas identified as hotspots of wild meat consumption in the region (Fig. 3b). We also highlight that, although the categorization of our 252 settlements was based on the direct experience of researchers working in the area (that is, the data providers), to predict consumption rates, we used a standardized categorization applicable across the region using available geographical layers^37^. Specifically, we classified settlements of up to 10,000 people as villages, urban areas with between 10,000 and 100,000 inhabitants as towns and agglomerates of more than 100,000 people as cities. This approach correctly classified 91% of the locations in our database (Supplementary Discussion). However, different cut points might be more accurate depending on the local context, particularly when discriminating between town and villages. To assess this risk and its implication on the final estimates, we ran an additional model predicting consumption rates for only two settlement types, urban and rural^37^ (Supplementary Table 5), which resulted in higher median consumption rates (Extended Data Fig. 5).

## The threat to wildlife populations

Our most recent estimate of annual wild meat consumption (1.10 × 10^6^ t in 2022) represents over half of the 2 × 10^6^ t of mammal standing biomass estimated to be produced by Central African forests in the year 2000^38^. More recent estimates are lacking, but we suspect that, 25 years on, the production of Central African forests is now lower due to ongoing forest loss and degradation^11^. Standing biomass estimates include all vertebrate species and, while our estimates also include the meat of vertebrates other than mammals (for example, birds = 0.7%, reptiles = 2.7%), large and medium terrestrial mammal species^39^ are the major target of hunting for food and income in proximity to rural villages^40,41^, and offtake rates for these species are likely to be unsustainable. Arboreal species, such as primates, are also threatened by regional increases in gun hunting^42^. In the absence of sustainable wildlife management, we expect that the abundance and diversity of the wild animals around Central African rural communities will deteriorate^39,42^, affecting the livelihood and subsistence of communities that (1) do not have a history of livestock rearing^43^; (2) use domestic animals as an asset for insurance to be used in times of economic or nutritional need, rather than a primary source of food^44^; (3) do not have access to domesticated meat^27,45,46^; (4) lack access to the sea, large rivers or lakes and consume fish only seasonally^15^.

## A critical component of human nutrition

**Extended Data Fig. 6:**
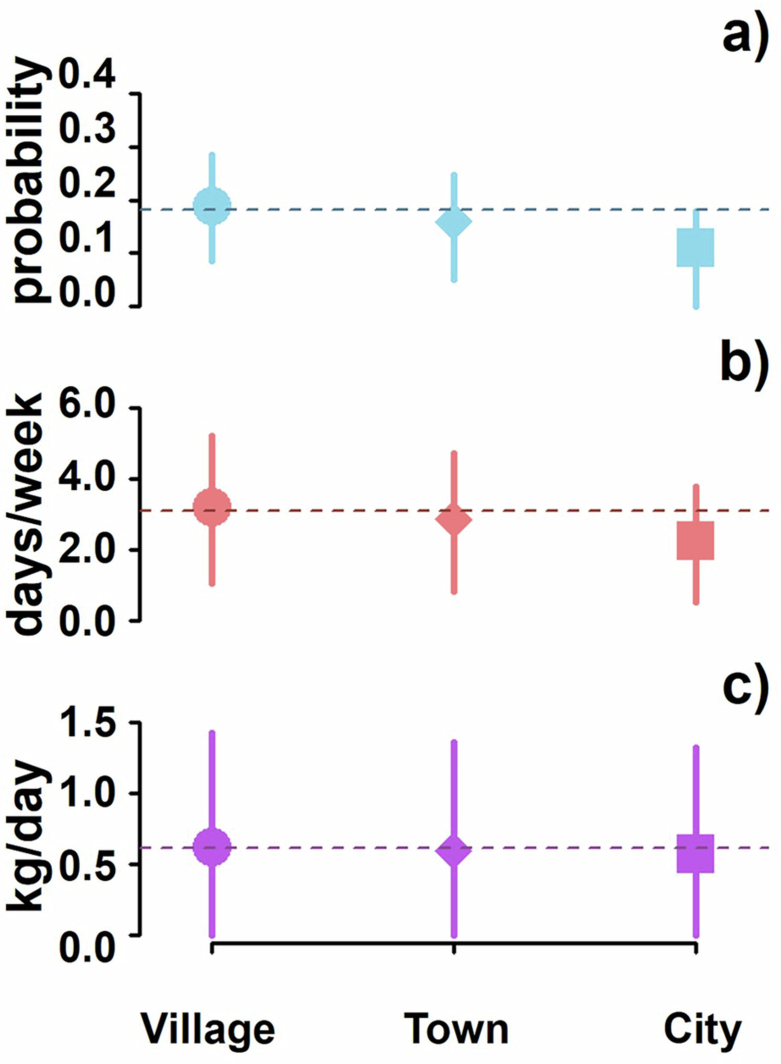
Estimated wild meat a) consumption probability (blue), b) frequency of consumption (days/week; pink) and c) quantity consumed (g/AME/day; purple) in different settlement types. *Settlement types*: villages (<10,000 inhabitants), towns (>10,000 and <100,000), cities (>100,000) obtained from n = 4,000 posterior draws. *Coloured shapes*: mean of the posterior distribution (Circles = villages; Diamonds = towns; Squares = cities); *Solid bars*: 95% Highest Posterior Density Intervals; *Dashed lines*: Mean of the posterior distribution considering all settlement types. Note that consumption rates are the product of (a), (b) and (c) – see Eq. 1.

Rural households in villages showed the highest probability of consuming wild meat as well as the highest frequency of consumption (Extended Data Fig. 6), resulting in a (median) consumption rate of 56 g per AME per day (mean = 113; s.d. = 175; Fig. 3c), or 20% of the recommended daily protein intake (40% if we consider the mean estimated value). In the aftermath of the COVID-19 pandemic, there were calls for a complete global ban of wild meat consumption and trade, based on the assumption that eliminating wild meat from food systems could reduce human–wildlife contacts and therefore the spread of novel zoonotic diseases^47^. Our study shows the critical role that wild meat currently has in providing nutrients required for a healthy diet and food security for rural communities in central Africa^48^. It also suggests that legal and sustainable use of non-protected wild animals in rural areas, including support for sustainable management, may be one way to safeguard the diets of millions of people, especially as other factors such as war^49^ and climate change^50,51^ further strain food security. While endangered and slow reproducing species must be protected according to national and international legislation, clear national laws enabling the management of remaining species, co-designed with Indigenous Peoples and Local Communities^52^, would improve the sustainability of the wild meat sector in rural settings^25^. However, site-specific assessments of wildlife availability and sustainable levels of extraction are fundamental prerequisites of any action aiming to achieve sustainable wildlife management^53^.

## A commodity rooted in culture

Despite lower daily quantities of consumption per person (towns: 38 g per AME per day, mean = 83, s.d. = 139; cities: 16 g per AME per day, mean = 45, s.d. = 90; Fig. 3b), the hotspots of total biomass consumed were found in areas where large numbers of people are concentrated (Fig. 3d). As above, we stress that, owing to the high uncertainty associated with the highest predicted amounts of biomass consumed (Extended Data Fig. 4c), absolute values should be considered with care. Our study predicts that cities and towns (>10,000 inhabitants) might be responsible for the consumption of around 40% (approximately 0.6 × 10^6^ t) of all the wild meat hunted in the region. Although major cities provide access to several alternatives, wild meat is still perceived as healthier than imported domesticated meats^27,45^, it maintains some cultural traditions and, where more expensive than domestic or imported alternatives, acts as a status symbol^27^. To satisfy urban demand, commercial hunters extract large numbers of animals from remote areas^54^, while subsistence hunters might also increase their hunting effort and sell what is not consumed in the household to generate income^55^. As a result, the proportion of wild meat extracted from tropical forests in sub-Saharan Africa that was then sold increased from 34% to 72% over the past 20 years^42^. A substantial reduction in wild meat demand from the major cities of the region is essential and should be a priority for all policies aiming to slow down or halt defaunation^3^. Substantial behavioural changes among urban populations are required, and we propose that tailored demand reduction could be successful at reducing wild meat consumption^56^.

**Extended Data Fig. 7:**
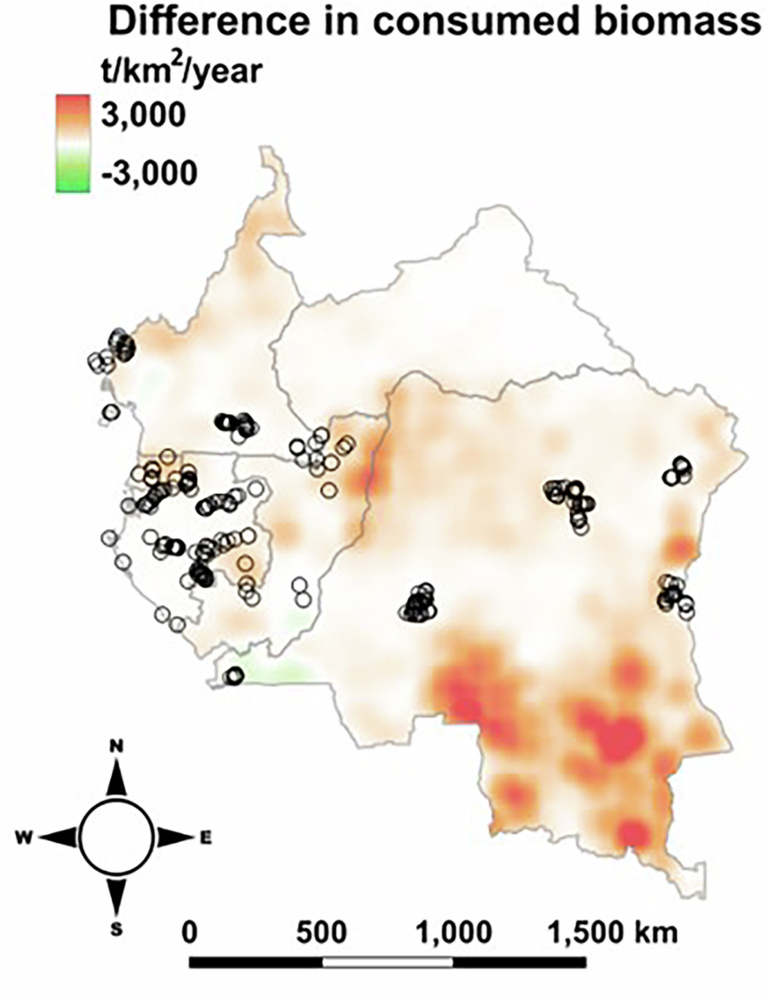
Change in total biomass consumed between 2000–2010 and 2011–2021. *Black circles:* surveyed locations. A detailed discussion of this map is provided in the Supplementary Discussion. Credit: country outlines, https://geoportal.icpac.net/ under an Open Database License ODbL 1.0; map created with QGis 3.22.1^69^.

Our study highlights another key issue^3^: the demand of wild meat in provincial urban areas^57^. These provincial cities and towns are relatively remote, with access to wild areas^58^ and sometimes weaker law enforcement than in larger or capital cities, allowing open access and free trade of wild meat^57^. Accordingly, consumption rates might not be too different from those observed in nearby rural areas^30^ (Fig. 3a,b), a scenario that differs from other tropical regions (like the Amazon) where urbanization favours the transition from consuming wildlife to domesticated animals^59^. In areas in which remoteness, access to wildlife, weak law enforcement, and expensive or unavailable alternatives interact with immigration from rural areas to provincial towns, we expect the demand for wild meat to further increase (Extended Data Fig. 7), negatively affecting the wildlife inhabiting the surrounding areas.

## Towards sustainable wild meat consumption

Using the most comprehensive database of wild meat consumption currently available, our analysis highlights how in Central Africa the demand for wild meat from an increasingly urbanized population is threatening the wildlife populations that underpin the livelihoods of many rural communities. Our study advances previous attempts to assess wild meat consumption in Central Africa in two major ways. First, in contrast with previous studies^36,60^, it provides spatially explicit regional estimates of wild meat consumption. Second, while our understanding of the factors promoting wild meat consumption was previously based on data from few locations, our approach highlights cross-regional commonalities. As such, the results of our study constitute a crucial evidence-based foundation for the development of targeted wild meat policies in Central Africa and possibly beyond. However, data were not available for large areas of Central Africa (Fig. 1). We encourage future field studies to target areas currently lacking consumption data where our prediction map suggests (1) the largest impact in terms of consumed wild meat biomass (Fig. 3d and Extended Data Fig. 3c) and (2) the highest importance of wild meat in people’s diet (Fig. 3b,c and Extended Data Fig. 3a,b). Such studies would not only allow the validation of our model but would also be essential to improve our understanding of wild meat consumption in Central Africa and to assess areas where interventions may be most needed.

The sustainable use of wildlife—preventing defaunation while securing long-lasting access to an important source of food in one of the most food insecure regions in the world—is among the pillars of the global biodiversity framework of the Convention on Biological Diversity^5^ and is critical to the achievement of the SDGs set by the United Nations^4^. Reducing the role of wild meat in the current food system will require increases in the regional production, importation and distribution of healthy, safe and culturally appropriate alternatives. This will require considerable investments in national food systems, developing alternative protein sectors (for example, poultry and fisheries) and providing alternative sources of revenue or employment to those people involved in the wild meat trade^14^. To this aim, information on local food needs and preferences are crucial to select the sectors to be developed in specific contexts^45^.

With climate change expected to affect the productivity of African terrestrial ecosystems in the coming years^61^, improvements in local food system productivity should be designed as far as possible to avoid the extensive conversion of forest to agricultural land^62^ that have triggered disastrous environmental consequences across the world^63^. Agricultural science and technology have made enormous advances in recent years in determining appropriate crop and livestock varieties for the tropics, promoting traditional climate resilient varieties, soil improvements and intensified organic agricultural practices that reduce fertilizer use and promote yield. Modern food systems in Central Africa could avoid the environmental degradation and, ultimately, high costs that have typified the development of other world regions. However, their development will require strong political support at the regional and global level, including the adoption of transformative systemic solutions, such as steady-state and post-development models^64^.

Central African governments, international and national institutions and non-governmental organizations operating in the region should ensure sustainable management of hunting and trade for the benefit of the Central Africa ecosystems^62^, wildlife^42^ and rural communities^65^, and increase efforts to reduce the consumption of wild meat in cities^66^. While a substantial reduction of the approximately 0.6 million tonnes of wild meat consumed in towns and cities today must be accompanied by the rapid development of alternative livelihood opportunities for rural communities, it is an essential step in the creation of a sustainable, equitable and legal wild meat sector^67^.

## Methods

### Data

#### Compiling the database

We compiled our database by identifying all sources providing data on wild meat consumption in seven Central African countries between 2000 and 2022: Cameroon, Central African Republic, Democratic Republic of the Congo, Equatorial Guinea, Gabon and Republic of the Congo. We also included data from the Cross River Forest landscape in southeast Nigeria, as forests there are contiguous with protected areas in Cameroon. We considered peer-reviewed articles, technical reports, PhD and master’s dissertations, online data repositories and unpublished data, adopting a snowball sampling approach^70^ to search reference lists and online libraries. We used wildmeat, wild meat, bushmeat, bush meat and viande de brousse as main keywords, and consumption, nutrition and food as secondary keywords. We defined a study as a set of data collected using a single methodology in a specific study area over a determined timeframe. In this way, each data source could provide more than one study. For example, large projects that monitored multiple regions in different countries were split so that each study area represented a single study. For consistency, we restricted our research to studies investigating wild meat consumption at the household level, discarding those monitoring consumption of individual consumers who could not be aggregated to households, for example by enquiring people randomly met in the streets, a methodology mostly used in cities, where household surveys are difficult to implement. This is also the reason for the limited number of cities included in our database. Most recent studies used KoboToolBox (https://www.kobotoolbox.org/) and different versions of the KoboCollect App (v.2.020.40 and subsequent releases). Older studies recorded data with pen and paper. When possible, we downloaded the raw data from online resources (such as publicly available databases). Alternatively, we contacted the authors to request the raw data.

#### Data preparation

Individual studies underwent a preliminary phase of data cleaning and standardization to conform with the format required by the database https://www.wildmeat.org/. The resulting database included studies providing three different datatypes: (1) consumption/non-consumption; (2) frequency of consumption; (3) quantity consumed, each requiring specific formatting of the raw data.

Consumption/non-consumption data were provided by all studies and were therefore available for all recall events in our database (that is, at the recall level). If a household declared to have consumed wild meat during the specific recall, we recorded a ‘consumption event’ and coded the recall as 1. By contrast, if no wild meat consumption was reported during the recall period, we coded the recall as 0. Here, we considered a minimum recall period of 24 h (see the ‘Statistical analyses’ section for a description of how the number of monitored days was accounted for in the analyses). Therefore, if wild meat consumption was available for multiple meals within 24 h, we aggregated the information available for single days. In other terms, if wild meat was consumed twice (for example, in the morning and afternoon) within the same 24 h, we coded the 24 h recall as 1.

Frequency of consumption—defined as the number of consumption events recorded over the number of days a household’s consumption was monitored—was provided by 24 out of the 30 studies included in our analysis, representing 11,582 households and 107,896 recall events. For studies that recorded the frequency of consumption in categories such as daily, weekly and monthly, we calculated the frequency on a scale from 0 to 1. For example, if a household reported consuming wild meat monthly, it was assigned a frequency of consumption of 0.033 (12/365 days). However, for studies that recorded several recall events from the same household, we calculated the frequency of consumption by dividing the number of consumption events, by the total number of recalled days. For example, if a household was interviewed for 6 days about wild meat consumption over the previous 24 h and consumption occurred on two occasions, we calculated frequency as 2/6 days = 0.33. In this way, the frequency of consumption referred to individual households, rather than to single recall events. Each household had to be monitored for a minimum of 2 days to be considered (see the ‘Statistical analyses’ section for a description of how the number of monitored days was accounted for in the analyses). In other words, studies that recorded wild meat consumption over 24 h, in a single occasion for each household, were considered as consumption/non-consumption data.

Finally, 19 studies provided the quantity consumed (in g, kg, or local units such as leg, piece or entire animal) by the households over a recall period, as well as information on the wild animal species consumed. These studies included 9,189 households and 105,503 recall events. Data were available at the recall level, and we standardized the data as the quantity (in kg) consumed per household per day. Therefore, if the recorded quantities represented the cumulative consumption over a recall period of >24 h, we divided the reported quantities by the duration of the recall (in days). So, if a household reported having consumed 12 kg of undressed meat over a 72 h recall (that is, 3 days), we considered the quantity consumed by the household in a day to be 4 kg. Conversely, if quantities consumed were recorded for multiple meals within 24 h, we summed the quantity of wild meat reported for a single day. Thus, if a household reported to have consumed 0.5 kg of wild meat in the morning and 1 kg in the evening, the quantity of wild meat consumed by the household in that day would be 1.5 kg. Finally, when consumed quantities were reported in local units of measure (Extended Data Table 1), we estimated consumed kilograms following procedures specific for each unit. If the consumed units were reported in local units (such as entire, half, quarter or gigot), we assigned the species-specific average mass value using data available from the literature^71^ or empirical data obtained in Gabon by the authors of this study (L.C., Dibouka, 2001–2010; D.F. and D.C., Lastourville area, 2021). For all other units, including piece, pile and plate, we used estimated conversion factors, based on empirical observation collected by various authors of this study (L.C., K.A., F.S., D.D.). Because in Central Africa wild meat is generally sold and cooked along with bones and sometimes skin, we considered the quantities in our database as the quantity of undressed meat (in kg) consumed per household per day.

### Ethics statement

The procedure used to compile the database was approved by the ethics review committee of CIFOR/ICRAF (SLF6430000-UFW044-AI2; 13/12/2021) and included the anonymization of all sensitive data. All included studies obtained (1) an ethical review of data collection protocols, (2) the agreement of the local communities (focal groups/authorization of the communities’ representatives) before data collection; (3) prior informed consent from all respondents (Extended Data Table 1).

### Statistical analyses

Our model jointly analyses the datatypes described above to estimate consumption rates in the region and investigate drivers of consumption. It is therefore composed of three submodels. The first estimates wild meat consumption probability using consumption/non consumption data as a function of relevant predictors and accounting for spatial autocorrelation between sites. The second submodel models wild meat frequency of consumption as a function of predictors, and, as above, accounts for spatial autocorrelation. The third submodel investigates predictors of daily quantity of wild meat consumed individually, using the AME transformation^31^ (see below for details).

### Estimating wild meat consumption rates

We defined the wild meat consumption rate as the daily quantity of wild meat consumed per AME, using the following formula:

1 $$\mathrm{Consumption}\,\mathrm{rate}=\mathrm{consumption}\times \mathrm{frequency}\times \mathrm{quantity}$$

Where consumption represents a binary output, 0 or 1, of wild meat consumption, where 0 = non consumption and 1 = consumption; frequency represents how often is wild meat consumed on a scale from 0 (never) to 1 (every day); and quantity is the quantity (in kg) of wild meat consumed per AME in 24 h.

#### Consumption probability

The first level of our model estimated the consumption probability from binary data of consumption/non-consumption. Although binary data are the least informative towards the estimation of wild meat consumption, this submodel was important to include more studies into our analysis and increase the study coverage. Moreover, all datatypes could be scaled down to binary consumption/non-consumption data, and we were therefore able to obtain data for all recall events. Finally, modelling consumption probability enabled us to deal with the large proportion of non-consumption records (that is, 84%). We modelled the probability of consumption *π* for each recall event *r* as

2 $${\mathrm{consumption}}_{{r}} \sim \mathrm{Bernoulli}({\pi }_{{r}})$$

Where consumption is a vector of consumption/non-consumption data of length *R*, with *R* equivalent to the number of recall events in our database.

We defined *π* as a function of explanatory variables with logit-link, as

3 $$\mathrm{logit}({\pi }_{r})={\alpha }_{0}+{\alpha }_{1}\times {V1}_{r}+\ldots +\alpha k\times {{Vk}}_{r}+{\alpha }_{k+1}\times {\mathrm{days}}_{r}$$

Where *α*_0_ is the intercept; *α*_1_ to *α*_*k*_ are parameters specific to each variable *V* (*n* = *k*) included in the model, slopes for continuous variables or factors, for categorical variables.

Here we accounted for the fact that longer recalls would increase the probability of recording at least on consumption event, by including the parameter* α*_*k + *1_, defining the increment in *π* as a function of the recall duration in days.

#### Frequency of consumption

The second level of the model estimated the frequency of wild meat consumption, that is, how often a household reportedly consumed wild meat. If frequency data were not available, we assigned a missing code (that is, −1). As events with no consumption of wild meat were modelled in the previous submodel, and we modelled frequency conditional on being >0 (that is, a frequency of 0 was not considered) as

4 $${{\rm{f}}{\rm{r}}{\rm{e}}{\rm{q}}{\rm{u}}{\rm{e}}{\rm{n}}{\rm{c}}{\rm{y}}}_{h} \sim {\rm{B}}{\rm{e}}{\rm{t}}{\rm{a}}({\varphi }_{h}\times \kappa ,(1-{\varphi }_{h})\times \kappa )$$

Where frequency is a vector of length* H*, equivalent to the number of households included in our study; *φ* is the mean frequency of consumption for household *h* and *κ* is the sample size of the beta distribution.

Here, we wanted to account for the possibility that the precision of the observed frequency is conditional on the number of monitored days. For example, if a household was interviewed on a single day and asked how often it consumed wild meat over a year, we expected the uncertainty to be higher than cases in which a household was visited every day and asked about what they ate in the previous 24 h. We assumed that the latter case as the ideal scenario (*n* = 365 monitored days), where we could be certain that the recorded frequency was correct, with minimal associated error. Conversely, we assumed the first (*n* = 1 monitored days) to be the case with the highest uncertainty, as if a household was only visited once and asked what they ate in the previous 24 h (*n* = 1 monitored days). We therefore modelled *φ* for each household *h* as

5 $$\mathrm{logit}({\varphi }_{h}) \sim \mathrm{Normal}({\underline{\varphi }}_{h},{\Sigma }_{h})$$

where $\underline{\varphi }$ is the mean of the frequency of consumption on the logit scale and Σ its s.d., which we modelled conditional on the number of monitored days as

6 $${\Sigma }_{h}=\sigma \times (365-{\mathrm{mdays}}_{h})$$

Here, mdays is the sum of monitored days for household *h*; and *σ* is the reduction rate in Σ for each monitored day, that is,* σ* scales to 0 if 365 days were monitored and is maximum if only 1 day was monitored.

Finally, we defined the mean frequency of consumption $\underline{\varphi }$ for household *h* as a function of explanatory variables with logit-link, as

7 $$\mathrm{logit}({\underline{\varphi }}_{h})={\beta }_{0}+{\beta }_{1}\times V{1}_{h}+\cdots +\beta k\times V{k}_{h}$$

Where *β*_0_ is the intercept, *β*_1_ to *β*_*k*_ are parameters specific to each variable *V* (*n* = *k*) included in the model (slopes for continuous variables; factors for categorical variables).

To improve sampling efficacy of our model, we used the equivalent non-centred parameterization of equation (5), defined as follows^72^:

8 $${\varphi }_{h}={\mathop{\varphi }\limits_{\_}}_{h}+{\Sigma }_{h}\times {\tau }_{h}$$

#### Quantity consumed

Finally, the third level of our model estimated the daily quantity in kg consumed per AME. Here we used data provided by studies that recorded the weight (in g, kg or local units of measure) consumed in a household over a certain recall period. If the quantity consumed was not available, we assigned a missing code (that is, −1). As the probability of consuming wild meat on a certain day and the frequency of wild meat consumption were analysed in previous submodels, we modelled the daily quantity consumed per AME in recall event *r* conditional on it being >0 (that is, kg of consumed wild meat were recorded and > 0), as

9 $${{\rm{Q}}{\rm{u}}{\rm{a}}{\rm{n}}{\rm{t}}{\rm{i}}{\rm{t}}{\rm{y}}}_{r}/{{\rm{A}}{\rm{M}}{\rm{E}}}_{r} \sim {\rm{G}}{\rm{a}}{\rm{m}}{\rm{m}}{\rm{a}}({\mu }_{r}\times \theta ,\theta )$$

where quantity is a vector of length equivalent to the number of recall events *R* considered in our study providing the quantity (in kg) of wild meat consumed per household *h* in recall *r*; AME is a vector of length *R*, storing the number of AME registered for each recall event *r*; *μ* is the mean quantity (in kg) of wild meat consumed per AME in recall *r*; and *θ* is the scale parameter of the gamma distribution.

Finally, we defined *μ* as a function of explanatory variables with log-link, as

10 $$\log ({\mu }_{r})={\gamma }_{0}+{\gamma }_{1}\times {\mathrm{AME}}_{r}+{\gamma }_{2}\times {V1}_{r}+\ldots +{\gamma }_{k}\times {{Vk}}_{r}$$

Where *γ*_0_ is the intercept, *γ*_1_ is covariate-specific slopes for the number of AME participating in a recall event; *γ*_2_ to *γ*_*k*_ are parameters specific to each variable *V* (*n* = *k*) included in the model (slopes for continuous variables, or factors for categorical variables).

#### Spatial autocorrelation

We expected geographically close locations to be more likely to share similar patterns of wild meat consumption. Our model therefore also included a spatial autocorrelation component, allowing for the similarities between two sites to decrease as the distance grows, using the quadratic kernel function. Specifically, we implemented a latent Gaussian process regression, exploiting the Euclidean distance between locations to estimate the covariance of each pair at different distances from each other^72^. In practice, we first built a distance matrix with dimension equal to the number of locations *l* in our model, *D*_*l*__,__*j*_ and then implemented the quadratic kernel function to build the covariance matrix *X*_*l*__,__*j*_

11 $${X}_{l,j}={\zeta }^{2}\exp (-{\rho }^{2}{D}_{l,j}^{2})+\delta $$

Where *ζ* is the marginal s.d., representing the maximum covariance between sites,* ρ* is the rate of decrease in covariance (that is, length scale) and *δ* is a small positive scalar (that is, 10^−9^), added to the diagonal of *X* to ensure that it remains positive.

The resulting covariance matrix was then converted to a Cholesky factor *LX*_*l*__,__*j*_ (that is, the product of the lower triangular matrix and its conjugate transpose) for more efficient numerical solution. Finally, *LX* was multiplied by **η**, a vector of length equal to the number of locations, used to generate a multivariate normal vector **ε**, corresponding to the latent Gaussian process^72,73^.

#### AME imputation

The number of AME per household was unavailable for 55.9% of the recall events. Having included AME in the linear model for *μ* (equation (10)), we were able to use Bayesian imputation to estimate missing values of AME, a method that is independent of the percentage of missing values in the dataset^74^. To do that, we assigned a distribution to the missing values, such as

12 $$\mathrm{missing}\,{\mathrm{AME}}_{m} \sim \mathrm{Normal}(\nu ,\psi )$$

Where missing AME_*m*_ is a vector of length equal to the number of missing values *M*;* ν* is the mean number of AME present in a recall event; and *ψ* its s.d. In this way we obtained the vector AME merged, of length equivalent to the number of recall events *R* and composed of both observed and estimated (that is, imputed) values of AME.

#### Priors

We set weakly informative priors to all our parameters (Supplementary Table 1), providing the model with enough information to avoid exploring impossible values^75^. In the case of the imputation of missing AME, we centred the mean *ν* in equation (12) to the mean number of AME, $\underline{AME}$, calculated from available data, that is, 5.09.

### Simulation study

Before running the model on real data, we evaluated its accuracy in retrieving the parameters of interest in a simulation study in which we investigated three different scenarios of coverage of the study area: 5%, 10% and 15%, similar to the coverage of our data (that is, 7.5% of our prediction grid; Supplementary Fig. 1). For that, we used a simple version of the model described above, including 2 continuous variables and 1 categorical variable.

We created a study area composed of 900 cells, and divided it into three regions, with different characteristics. For each cell, we simulated the mean value of two continuous variables *V*_1_ and *V*_2_. The first region (number of cells = 360) was simulated having high *V*_1_ and low *V*_2_. The second (180 cells), as having high *V*_2_ and low *V*_1_. Finally, the third (360 cells) was simulated with intermediate values of *V*_1_ and *V*_2_. For simplicity, we allocated one location in each cell and considered it as a cluster of villages. We then calculated a distance matrix of the simulated location and created a varying number of households according to the cells’ features. If the site fell within region one, it was given a low number of households (mean = 40). Region two had the highest mean number of households (mean = 100) and region three an intermediate number (mean = 65). We then simulated the number of AME for each household, using a mean of 5 AME per household, and calculated the total number of AME within the study area. We also assigned a categorical variable *V*_3_ (2 levels) to each household, with the first level being less frequent (30%) than the second (70%).

For each household, we simulated (1) consumption probability *π* (equations (2) and (3)); (2) the frequency of consumption *φ* (equations (7) and (8)) and (3) the mean quantity consumed *μ* (equations (9) and (11)), defining their mean values using the simulated variables. Specifically, in (1) the mean consumption probability was simulated as a function of *V*_1_,* V*_3_ and spatial autocorrelation **ε**, making use of the distance matrix between sites described above. In (2) the mean frequency of consumption was a function of *V*_2_, and *V*_3_. In (3) the mean quantity consumed was a function of *V*_2_ and the number of AME in the household. In all of the models, we set intercept and slopes (for continuous variables) varying by region (Supplementary Table 2).

By averaging the values of all simulated households, we calculated the ‘real’ average (1) consumption probability, (2) frequency of consumption and (3) quantity consumed per AME in the region, calculated consumption rates applying equation (1) and obtained the number of tonnes consumed in the study area by summing up the product of the consumption rates and the number of AME simulated in each cell (equation (29)).

We then randomly selected a number of locations, conditional on the coverage scenarios described above. All those selected were assumed to provide information on wild meat consumption/non-consumption. However, we also assumed that only 80% of those provided information on frequency of consumption and only 50% gave information on quantities of wild meat consumed. We also selected a proportion of households within each surveyed cell as well as a subset of household (20%) for which we assumed that the number of AME was unknown. Finally, we simulated (1) the number of monitored days for each selected household, (2) the uncertainty around the real frequency value conditional on the number of monitored days (that is, longer monitoring = lower uncertainty) and (3) the observed frequency values by applying the obtained uncertainty to the real simulated value of each household (equation (5)).

For each scenario, we generated 100 databases and run 1 chain of 2,000 iterations (warmup = 1,000) for each of them (*n* = 100) in R (v.4.2.0)^76^ using Rstan (v.2.26.11)^77^. We verified the accuracy of our model by comparing the posterior distribution of the parameters estimated in each scenario (from the 100 samples aggregated) with the true simulated values. The results of the simulation are provided in the Supplementary Results.

### Correlates of wild meat consumption

To investigate the factors driving wild meat consumption in Central Africa, we evaluated a set of variables available at different levels (Supplementary Table 3): (1) the study level included information specific to the year and design of the studies included in the analysis; (2) the site level provided data relative to the sites where the studies were conducted, including geographical layers available for the entire region; (3) the household level provided information specific to characteristics of each household; and (4) the recall-level data specific to each recall event. Below, we describe continuous and categorical variables, state our hypothesis with respect to the effect on wild meat consumption rates and describe the process to format the data as used in the analysis. However, as random factors were simply identifiers (from 1 to *n*) of specific studies, sites, households and recalls, they did not require any data processing and are not mentioned below.

#### Study type

Wild meat consumption studies are generally conducted using recall interviews, where respondents are asked whether (consumption/non-consumption) or how often (that is, frequency of consumption) they consumed wild meat, and how much of it they consumed (quantity consumed), over a certain period of time, called the recall period. The studies included in our analysis used different recall periods, from 24 h to an entire year. A different approach was represented by ‘cooking pot’ studies, in which respondents were not asked what they consumed, but rather what they cooked. As in Central Africa, it is common to share what is cooked with other households^78^, we expected cooking-pot studies to overestimate the quantity consumed per capita in the interviewed household, as part of the cooked meat might have been consumed elsewhere.

##### Hypothesis

Cooking-pot studies tend to overestimate quantities consumed, but not the frequency of consumption or consumption/non-consumption.

##### Data processing

Studies that recorded quantities of consumed wild meat and used a recall period of 24, 48 or 72 h, were given a dummy study type (ST) value of 1; longer recall periods (1 week, 1 month or 1 year) were used only by studies focussing on the frequency or consumption/non-consumption and were assigned a ST value of 2; cooking-pot studies were given a ST value of 3 (Extended Data Table 1).

#### Location type

To fulfil nutritional requirements, Central African rural populations often have no/few alternatives to the consumption of wild meat. However, urban populations often do, particularly those living in metropolitan areas and capital cities, where affordable alternatives are available^79^. Here, wild meat consumption is less a matter of survival, and more of culture. In Central African cities, wild meat is perceived to be healthier than imported poultry and pork, and it represents both a way to maintain a connection with one’s place of origin (usually a rural area, where wild meat is the main source of protein) and a status symbol, as wild meat is generally more expensive than domestic alternatives^27^. In the region, another settlement type is represented by towns between 10,000 and 100,000 inhabitants, where alternatives are available but are generally more expensive than wild meat.

##### Hypothesis

Hypothesis: wild meat consumption probability and frequency of consumption are highest in the villages, and lowest in urban areas. We also expected the quantity consumed by AME to be higher in the cities (where wild meat is sometimes luxury product).

##### Data processing

None. The data collected in each site were given a dummy code (from 1 to 3; Supplementary Table 3), according to the settlement type.

#### Distance between locations

Wild meat consumption rates are known to vary with respect to many factors, including price, availability of wildlife and alternative sources of protein^15^ and seasonality^78^. Consumption rates are also likely to be driven by fine-scale characteristics at the site and household level, which are mostly expected to be cultural. Some of these factors have been measured in the past and were included in this study, but we suspected others unobserved factors could drive consumption rates in the region. For example, cultural features are shared by more neighbouring villages and change gradually as a function of distance. In other terms, locations closer to each other are more likely to share similar cultural and environmental features than those further apart.

##### Hypothesis

A substantial part of the variation in consumption rates can be explained by unmeasured characteristics shared between geographically related locations.

##### Data processing

We calculated the distance between each pair of locations included in our analysis by georeferencing the site and then using the Distance matrix algorithm in QGis (v.3.22.1)^69^, resulting in a square matrix *D* with dimension equal to the number of locations (*n* = 252), and zeroes on the diagonal.

#### Human population density

Human population density in Central Africa is growing at a 3% annual rate, increasing wild meat demand and, consequently, wildlife extraction rates. As human population density increases, wildlife becomes scarce, wild meat prices increases and consumption rates decrease^32^. However, even limited consumption rates from a large human population can have a major effect on the total amount of biomass consumed. An increasing number of people in the region are moving from rural to urban areas, and the urban population of Central Africa doubled between 2000 and 2020 (https://data.worldbank.org/indicator/SP.URB.TOTL).

##### Hypothesis

High human population density (HPD) values would result in lower consumption probability and frequency of consumption.

##### Data processing

We calculated HPD for each site included in our analysis using dynamic human population layer^80^. To provide a value representative of the surroundings of the site and not relative to a single point in space, we used QGis (v.3.22.1)^69^ to create a 40 km buffer around the georeferenced location of each site. This area represents the furthest distance that communities in Central Africa are willing to cover to procure wild meat^81^. We then averaged the values of HPD for each available year (that is, 2000, 2005, 2010, 2015, 2020) within each 40 km buffer and assigned it to each site according to the time the site was surveyed. In other terms, if a site was surveyed in 2002, it was given the value of HPD calculated for the year 2000; sites surveyed in 2013 were given the value calculated for the year 2015. Consequently, each household, and each recall related to a particular site, obtained the same value of HPD.

#### Remoteness

In Central Africa, remote areas are those where alternatives to wild meat are rarest, and even if available, cannot be afforded by most inhabitants^46^. Moreover, many communities do not have a history of livestock rearing^38^, and locally reared livestock is used as a security commodity in time of economic or nutritional need^44^.

##### Hypothesis

High remoteness (REM) values would result in a higher consumption probability and frequency of consumption, with either an opposite or a non-detectable effect on the daily quantity of wild meat consumed.

##### Data processing

We calculated REM for each site included in our analysis in the same way we did for HPD (see above) using a remoteness layer^82^ available for 2015 only (Supplementary Discussion). Therefore, REM values were assigned to each site independently of survey time.

#### Human development index

The human development index (HDI) is an indicator of human development, calculated as the geometric mean of the normalized indices of (1) life expectancy at birth, (2) average years (for adults >25 years) and expected years of schooling for children; (3) gross national income per capita^83^.

##### Hypothesis

High HDI values would result in lower consumption probability and frequency of consumption, with either an opposite or a non-detectable effect on the daily quantity of wild meat consumed.

##### Data processing

We allocated an HDI value to each site according to site-specific administrative level 1 and year of survey. Consequently, each site, household and recall related to a particular administrative level, obtained the same value of HDI.

#### Forest condition index

In Central Africa, a large proportion of consumed wild meat is sourced in the rainforest^20^. Intact habitats are essential to the persistence of abundant, healthy and diverse wildlife communities. Conversely, in human modified habitats, where forest is degraded, wildlife populations might be depleted and unable to provide a substantial amount of wild meat^64^. Consequently, wild meat consumption is most relevant in regions where the forest is healthy, wildlife is abundant and hunting is profitable, making wild meat a cheap source of food^15^.

##### Hypothesis

High FCI values would result in higher consumption probability and frequency of consumption, and in either a similar or non-detectable effect on the daily quantity of wild meat consumed.

##### Data processing

We calculated FCI for each site included in our analysis in the same way we did for HPD (see above). However, here the forest condition index layer^84^ was available for the year 2019 only (Supplementary Discussion). FCI values were therefore assigned to each site independently of survey time.

#### Education level

The education level attained in a household can be considered as a proxy of its wealth^85^. Higher education increases opportunities to find paid employment, which in turn gives access to more expensive sources of food^58^. However, poor job markets in rural areas limit the earning advantages of education. As such, we expected potentially opposing effects of education as a proxy for wealth in rural and urban areas. Where wild meat is cheaper than alternative sources of protein, education levels might have little effect on consumption rates^15^. However, in cities in which wild meat is expensive, education might be linked to higher consumption, as education is more likely to result in higher wealth in more vibrant job markets, and wealth is more likely to be used to purchase more expensive wild meat^27^. Accordingly, we investigated (1) the fixed effect of education level (ED) (not considering differences between location type) and (2) the interaction between ED and location type (LT) (that is, village, town, city).

##### Hypothesis

Households with higher education show lower consumption rates in rural areas (that is, villages), but higher rates in towns and cities. Education level has no effect on consumption rate when households from different settlement types are aggregated.

##### Data processing

Rural households where the reported highest education was primary (or no education) were given a dummy ED value of 1; town households where the reported highest education was primary (or no education) were given a dummy ED value of 2; city households where the reported highest education was primary (or no education) were given a dummy ED value of 3. In the fixed-effect model, these categories were aggregated by assigning an ED value of 1. Rural households that reported a secondary (or higher) education level were given an ED value of 4; town households that reported a secondary (or higher) education level were given an ED value of 5; city households that reported a secondary (or higher) education level were given an ED value of 6. In the fixed-effect model, these categories were aggregated by assigning an ED value of 2. Finally, households for which the education level was unknown were assigned ED value of 7 (in the interaction model) or 3 (in the fixed-effect model) regardless of the location type.

#### Household size

Finally, the number of people participating in a meal might affect the quantity of wild meat consumed. Assuming a household only has a certain budget to spend, or that the hunters in the household could only provide a certain amount of meat per day, the more people participate in the meal the smaller the quantity consumed per AME.

##### Hypothesis

Higher AME numbers present during a recall event result in lower quantities consumed per capita, and vice versa.

##### Data processing

If the number of AME was not calculated and the sex and age of each person present in the recall were available, we used the following formula^86^:

13 $$\mathrm{AME}=\mathrm{AM}\times 1+\mathrm{AF}\times 0.86+C(10\mbox{--}15\,\mathrm{years})\times 0.96+C(6\mbox{--}10\,\mathrm{years})\times 0.85+C(0\mbox{--}5\,\mathrm{years})\times 0.52$$

Where AM = adult male individual (>16 years old); AF = adult female individual (>16 years old); and *C* = Child. If a child’s age was not specified, we multiplied by 0.78, that is, the average between the three child multipliers. Similarly, if adult sex was not specified, we multiplied the number of adults by 0.93, that is, the average between the adult male and adult female multipliers. If the number of AME was available for the household but not for each recall event (in case of multiple recalls of the same household), we allocated the same AME value to each recall event related to a household. Finally, if no information regarding the age structure of the household was present, we coded with a missing code and imputed the value within the model (equation (12)).

### Model selection process

There is substantial debate on the best process to be used when deciding the explanatory variable to include in a model to avoid (1) spurious correlations and (2) overfitting, while at the same time achieving sufficient predictive power. Here, to reduce the probability of spurious correlations, we made use of our knowledge to select variables that we considered as potential drivers of wild meat consumption probability, frequency of consumption and quantity consumed, based on a priori hypotheses (Extended Data Table 2). Accordingly, we defined three full submodels based on the hypotheses and research questions described above.

To account for study-specific features in terms of the methodology and cultural and contextual characteristics of the study area, we used an intercept varying by study ID. According to our hypotheses, we considered all continuous variables important and included two categorical variables (1) education level ED, to evaluate whether higher education resulted in lower consumption rates, and (2) location type LT, to test our hypothesis of higher consumption rates in rural areas. In the model estimating quantity consumed, we included the number of AME present during the recall period, to test our hypothesis of higher AME resulting in lower quantities consumed per capita. To account for multiple recall events recorded for the same household in the models estimating the probability of consumption and quantity consumed, we also included household *H* as a random factor. When evaluating consumption probability, we also included the duration (in days) of the recall period days, on the assumption that longer recall periods have a higher probability of recording a consumption event.

To test the submodels for overfitting, we evaluated collinearity in the continuous variables included in each submodel by examining the pairs plot of the residuals^87^. In case of issues, we (1) included the spatial autocorrelation component, (2) checked whether collinearity issues remained by visually inspecting the pairs plot of the residuals, and (3) assessed whether the spatial component improved the model’s predictive power by comparing the expected log predictive density (ELPD) using the R package loo (v.2.5.1)^88^. We considered a significant increase in ELPD as an indication of the importance of the autocorrelation term. We considered two models to be equivalent if (1) the ELPD difference was ≤4; (2) the standard error of the difference was ≥ the difference in ELPD^89^. In case of persisting issues, we (4) evaluated the importance of each variable included by removing one at a time to investigate the submodels predictive power using loo. Here, a drop in ELPD with respect to the full model was considered an indication of the importance of the removed variable, that is, the larger the drop, the more important the variable that was removed. Conversely, a non-significant drop indicated a limited importance of the removed variable in explaining the data and suggested that the reduced and full model’s predictive power was similar.

We run each submodel (2 chains, 2,000 iterations, 1,000 warmup) using a subset of our database including 5 studies, spanning 3 countries and 2 time periods, and representing 10 sites, 401 households and 6,628 recalls. The results of the variables selection process are provided in the Supplementary Results.

### Past and present consumption rates

The final step in our study involved the prediction of consumption rates and the estimation of the amount of wild meat consumed per year in the entire region. To do so, we projected a grid of *J* cells over Central Africa, with *J* = 874 (Supplementary Fig. 1). Each cell *j* had size of 5,027 km^2^ (70.09 × 70.09 km), equal to the area of the circle (radius = 40 km) used to calculate the value of continuous variables for each site included in our analyses (see the ‘Correlates of wild meat consumption’ section).

As our data were mostly representative of the Central African forest region (Fig. 1 and Supplementary Discussion), we also restricted our predictions to an area that encompassed all the locations included in our analyses but excluded the Sahel regions of Cameroon and Central African Republic and southern Democratic Republic of the Congo (Supplementary Fig. 1), uncovered by the studies included in our database. To do so, we selected only cells intersecting a buffer around patches of continuous forest^68^ (>5,000 km^2^). To include areas of forest–savannah transition, we set a buffer radius equal to twice the side of the cells (that is, 140.18 km).

We defined 3 scenarios, predicting (1) past (2000–2010); (2) recent (2011–2021); and (3) present (2022) wild meat consumption in Central Africa. Within each cell, we calculated scenario specific values of (1) human population density, (2) remoteness, (3) human development index, (4) forest condition index, (5) education level, (6) location type and (7) number of AMEs, obtaining 7 vectors of length *J*, equal to the number of cells (see below for details).

To discriminate the parameters described above (observed and estimated) from those used for prediction, we annotated all predicted objects with the accent ^~^.

### Calculating prediction variables

Prediction variables for each cell *j*, and scenario *z* were calculated as follow.

#### Human population density

To calculate cell and scenario specific $\widetilde{\mathrm{HPD}}$ values, we used the human population density raster layer clipped over our prediction grid (Supplementary Fig. 2). We averaged HPD values from year 2000, 2005 and 2010 within cell *j* (past scenario), values from year 2015 and 2020 (recent scenario), and values from 2020 (that is, the most recent year).

#### Remoteness

Remoteness data were available for 2015 only. We therefore averaged values of the 2015 remoteness raster layer^82^ (Supplementary Fig. 3) within each cell *j* and use the obtained mean for all scenarios.

#### Human development index

We used subnational human development index^83^ values to calculate cell and scenario specific $\widetilde{\mathrm{HDI}}$. We averaged HDI values (years: 2000 to 2010, past; 2011 to 2019, recent; 2019, present scenario) within each administrative level available in the region (Supplementary Fig. 4). If a cell *j* was completely within the boundaries of an administrative level, it was assigned an averaged $\widetilde{\mathrm{HDI}}$ value calculated as described above. However, if a cell overlapped >1 administrative level, we first calculated the proportion of each administration within the cell and then calculated a weighted $\widetilde{\mathrm{HDI}}$ value, conditional on the area of each administrative level represented in the cells.

#### Proportion of natural terrestrial habitat

The forest condition index layer^84^ was available for 2019 only (Supplementary Fig. 5). We therefore calculated the average $\widetilde{\mathrm{FCI}}$ in 2019 within each cell *j* and use the obtained mean for each scenario *z*.

#### Location type

To predict consumption rates conditional on the type of settlements within each cell *j*, we needed a standardized categorization based on available data across the entire region. However, there is no regional, nor national, database available in Central African countries providing a classification for each settlement. In the same way, there are no databases of, for example, facilities present in each settlement, which could be used for a facility-based classification. As done by several other studies, either focusing specifically on wild meat^30,90,91^ or more generally on urbanization^92–95^, the only tested and replicable approach to (remotely) classify villages, cities and towns across Central Africa is to use population size. In our database, all villages (*n* = 224) had a population up to 10,000 people; towns (*n* = 24) had between 10,000 and 100,000 inhabitants; and cities (*n* = 4) all had more than 100,000 people (Supplementary Discussion). Accordingly, we used a global settlement type layer (resolution: 1 km^2^), available for year 2000, 2005, 2010, 2015 and 2020^37^. We used settlement type data from year 2005 (that is, the midpoint of the period 2000–2010), year 2015 (that is, the midpoint of the period 2011–2021) and year 2020 (that is, most recent available year) for the past, recent and present scenarios, respectively. For each scenario, we first reclassified the settlement type raster to discriminate between rural (coded as 1) and urban (coded as 2) inhabited areas. We then converted the reclassified raster to obtain a vector file of polygons representing urban settlements within the study area (Supplementary Fig. 6) and calculated the number of inhabitants by summing up human population data within each polygon^80^. Based on our population-based classification, we coded polygons as 1 (that is, village) if the number of people calculated within it was <10,000; as 2 (that is, town) if the estimated population was >10,000 but <100,000; and as 3 (that is, city) if the estimated population was >100,000. In this way, we obtained the estimated number of people present, as well as the proportion of people living in villages $\widetilde{{\rm{L}}{\rm{T}}1}$, towns $\widetilde{{\rm{L}}{\rm{T}}2}$ and cities $\widetilde{{\rm{L}}{\rm{T}}3}$, in each cell *j*.

#### Education level

To predict average education level within each cell, we first compiled a database composed of 11 ICF Demographic Health Surveys (DHS) (https://dhsprogram.com/methodology/survey-Types/dHs.cfm) and 14 UNICEF Multiple Indicator Cluster Surveys (MICS) (https://mics.unicef.org/) conducted between 2000 and 2021, and including information on the highest education level of 213,659 households, as well as the subnational district, that is, administration level 1, of the household (Supplementary Table 4 and Supplementary Fig. 7). For each cell *j*, we calculated the proportion of people that reported an education level ≥secondary and averaged values from within each administrative level covered by our prediction grid according to our scenarios. We used data from years 2000 to 2010 and 2011 to 2022 for the past and recent scenario, respectively, and data from the most recent available survey for each country (Supplementary Table 4), to calculate the present proportions of people attending secondary education. In cases in which a cell *j* was completely within the boundaries of an administrative level, the cell was assigned the specific calculated proportion of people attending secondary education $\widetilde{\mathrm{ED}}$. However, if a cell overlapped >1 administrative levels, we first calculated the proportion of the cell that fell within each administration and then calculated a weighted average of the proportion of people attending secondary education, conditional on the areas of each administrative level represented in the cell. As the analysis of the interaction between education level and location type did not show any clear indication of such effect (Extended Data Fig. 2 and Supplementary Table 5), for our prediction, we used the simplest approach and did not consider the interaction, but only the fixed effect of education.

#### Number of AMEs

For each scenario *z*, we multiplied values of $\widetilde{\mathrm{HPD}}$ previously calculated for each cell *j*, by 5,027, that is, the area of the cell in km^2^ to obtain the absolute number of people pop estimated to be present in each cell *j*. We calculated the proportion of children ${\mathrm{prop}}_{\mathrm{child}}$ in each cell *j* for each scenario *z*, using country-specific estimates of the proportion of children in the total population (https://data.worldbank.org/indicator/SP.POP.0014.TO.ZS) available from year 2000 to 2022. Similarly, we calculated the corresponding proportion of adults as

14 $${{\mathrm{prop}}_{\mathrm{adult}}}_{j,z}=1-{{\mathrm{prop}}_{\mathrm{child}}}_{j,z}$$

Finally, we obtained the predicted number of AME in each cell *j* and scenario *z* by adapting equation (13) as:

15 $${\widetilde{\mathrm{AME}}}_{j,z}={(\mathrm{pop}}_{j,z}\times {{\mathrm{prop}}_{\mathrm{adult}}}_{j,z}\times 0.5)\times 1+{(\mathrm{pop}}_{j,z}\times {{\mathrm{prop}}_{\mathrm{adult}}}_{j,z}\times 0.5)\times 0.86+{(\mathrm{pop}}_{j,z}\times {{\mathrm{prop}}_{\mathrm{child}}}_{j,z})\times 0.78$$

Here we assumed a sex ratio of 0.5 in the adult population, and used the same multipliers described above (see the ‘Drivers of wild meat consumption’ section) to convert the number of women and children into AME^86^. We calculated ${\mathrm{prop}}_{\mathrm{child}}$ as the average of the country-specific proportion of children from year 2000 to 2010 (past), 2011 to 2022 (recent) and 2022 (present) and obtained ${\mathrm{prop}}_{\mathrm{adult}}$ applying equation (14).

### Predicting wild meat consumption

We used the variables described above to predict cell and scenario-specific consumption probability $\widetilde{\pi }$, mean frequency of consumption $\widetilde{\varphi }$ and mean quantity consumed $\widetilde{\mu }$. For parameters varying by period *t*, we used the one specific to period 1 (2000–2010) for the past scenario, and the one specific to period 2 (2011–2021) for the recent and present scenarios. For random factors, we used the estimated average, annotated with the accent ‘-’. We weighed the parameters obtained for location type (equation (16), consumption probability; equation (17), frequency of consumption; equation (18), quantity consumed) and education level (equation (19), consumption probability; equation (20), frequency of consumption; equation (21), quantity consumed) by the proportion of people estimated living in villages, towns and cities and attending secondary school in each cell *j*, obtaining weighed parameters used for prediction by applying the following equations:

16 $${\widetilde{\alpha 1}}_{j,z}={\alpha 1}_{\mathrm{lt}1}\times {\widetilde{{\rm{L}}{\rm{T}}1}}_{j,z}+{\alpha 1}_{\mathrm{lt}2}\times {\widetilde{{\rm{L}}{\rm{T}}3}}_{j,z}+{\alpha 1}_{\mathrm{lt}3}\times {\widetilde{{\rm{L}}{\rm{T}}3}}_{j,z}$$

17 $$\widetilde{{\widetilde{\beta 1}}_{j,z}={\beta 7}_{\mathrm{lt}1}\times {\widetilde{{\rm{L}}{\rm{T}}1}}_{j,z}+{\beta 7}_{\mathrm{lt}2}\times {\widetilde{{\rm{L}}{\rm{T}}3}}_{j,z}+{\beta 7}_{\mathrm{lt}3}\times {\widetilde{{\rm{L}}{\rm{T}}3}}_{j,z}}$$

18 $${\widetilde{\gamma 2}}_{j,z}={\gamma 2}_{\mathrm{lt}1}\times {\widetilde{{\rm{L}}{\rm{T}}1}}_{j,z}+{\gamma 2}_{\mathrm{lt}2}\times {\widetilde{{\rm{L}}{\rm{T}}3}}_{j,z}+{\gamma 2}_{\mathrm{lt}3}\times {\widetilde{{\rm{L}}{\rm{T}}3}}_{j,z}$$

19 $${\widetilde{\alpha 5}}_{j,z}={\alpha 5}_{\mathrm{ed}1}\times (1-{\widetilde{\mathrm{ED}}}_{j,z})+{\alpha 5}_{\mathrm{ed}2}\times {\widetilde{\mathrm{ED}}}_{j,z}$$

20 $${\widetilde{\beta 5}}_{j,z}={\beta 5}_{\mathrm{ed}1}\times (1-{\widetilde{\mathrm{ED}}}_{j,z})+{\beta 5}_{\mathrm{ed}2}\times {\widetilde{\mathrm{ED}}}_{j,z}$$

21 $${\widetilde{\gamma 3}}_{j,z}={\gamma 2}_{\mathrm{ed}1}\times (1-{\widetilde{\mathrm{ED}}}_{j,z})+{\gamma 2}_{\mathrm{ed}2}\times {\widetilde{\mathrm{ED}}}_{j,z}$$

Finally, we generated predicted consumption probability $\widetilde{\pi }$, mean frequency of consumption $\widetilde{\varphi }$ and mean quantity consumed $\widetilde{\mu }$ by replacing variables at the recall and household levels in the linear models specific to each submodel (Extended Data Fig. 1a), with those calculated for the prediction grid:

22 $$\mathrm{logit}({\widetilde{\pi }}_{j,z}) \sim \underline{\alpha }+{\widetilde{\alpha 1}}_{j,z}\times {\widetilde{\mathrm{HPD}}}_{j}+\alpha 2\times {\widetilde{\mathrm{HDI}}}_{j}+\alpha 3\times {\widetilde{\mathrm{REM}}}_{j}+\alpha 4\times {\widetilde{FCI}}_{j}+{\widetilde{\alpha 5}}_{j,z}+\underline{\alpha 6}+\alpha 7$$

23 $$\mathrm{logit}({\widetilde{\varphi }}_{j,z}) \sim \underline{\beta 0}+{\widetilde{\beta 1}}_{j,z}\times {\widetilde{\mathrm{HPD}}}_{j,z}+\beta 2\times {\widetilde{\mathrm{HDI}}}_{j,z}+\beta 3\times {\widetilde{\mathrm{REM}}}_{j}+\beta 4\times {\widetilde{\mathrm{FCI}}}_{j}+{\widetilde{\beta 5}}_{j,z}$$

24 $$\log ({\widetilde{\mu }}_{j,z}) \sim \underline{\gamma 0}+\gamma 1\times \underline{\mathrm{AME}}+{\widetilde{\gamma 2}}_{j,z}+{\widetilde{\gamma 3}}_{j,z}+{\underline{\gamma 4}}_{st}+\underline{\gamma 5}$$

Consequently, we generated the predicted consumption, frequency and quantity (see description of equation (1)) for cell *j* and scenario *z* using the following equations:

25 $$\widetilde{{{\rm{c}}{\rm{o}}{\rm{n}}{\rm{s}}{\rm{u}}{\rm{m}}{\rm{p}}{\rm{t}}{\rm{i}}{\rm{o}}{\rm{n}}}_{j,z}}=\mathrm{Bernoulli}({\widetilde{\pi }}_{j,z})$$

26 $$\widetilde{{{\rm{f}}{\rm{r}}{\rm{e}}{\rm{q}}{\rm{u}}{\rm{e}}{\rm{n}}{\rm{c}}{\rm{y}}}_{j,z}}=\mathrm{Beta}({\widetilde{\varphi }}_{j,z}\times \kappa ,(1-{\widetilde{\varphi }}_{j,z})\times \kappa )$$

27 $$\widetilde{{{\rm{q}}{\rm{u}}{\rm{a}}{\rm{n}}{\rm{t}}{\rm{i}}{\rm{t}}{\rm{y}}}_{j,z}}=\mathrm{Gamma}({\widetilde{\pi }}_{j,z}\times \theta ,\theta )$$

In this way, we estimated consumption rates in each cell *j*, and for each scenario *z*, using equation (1) as

28 $$\widetilde{{{\rm{c}}{\rm{o}}{\rm{n}}{\rm{s}}{\rm{u}}{\rm{m}}{\rm{p}}{\rm{t}}{\rm{i}}{\rm{o}}{\rm{n}}{\rm{r}}{\rm{a}}{\rm{t}}{\rm{e}}}_{j,z}}=\widetilde{{{\rm{c}}{\rm{o}}{\rm{n}}{\rm{s}}{\rm{u}}{\rm{m}}{\rm{p}}{\rm{t}}{\rm{i}}{\rm{o}}{\rm{n}}}_{j,z}}\times \widetilde{{{\rm{f}}{\rm{r}}{\rm{e}}{\rm{q}}{\rm{u}}{\rm{e}}{\rm{n}}{\rm{c}}{\rm{y}}}_{j,z}}\times \widetilde{{{\rm{q}}{\rm{u}}{\rm{a}}{\rm{n}}{\rm{t}}{\rm{i}}{\rm{t}}{\rm{y}}}_{j,z}}$$

And calculated the biomass consumed (in kg) in each cell *j*, and for each scenario *z*, as

29 $$\mathrm{kg}\,{\mathrm{consumed}}_{j,z}=\widetilde{{{\rm{c}}{\rm{o}}{\rm{n}}{\rm{s}}{\rm{u}}{\rm{m}}{\rm{p}}{\rm{t}}{\rm{i}}{\rm{o}}{\rm{n}}{\rm{r}}{\rm{a}}{\rm{t}}{\rm{e}}}_{j,z}}\times \widetilde{{{\rm{A}}{\rm{M}}{\rm{E}}}_{j,z}}\times 365$$

Where the $\widetilde{\mathrm{consumption}\,\mathrm{rate}}$ is the result of equation (28) for cell *j* and scenario *z*, $\widetilde{\mathrm{AME}}$ is the number of AMEs estimated to be present in each cell *j* for scenario *z*; 365 is the number of days in a year.

Finally, by summing up the predicted consumption rates, we calculated the total quantity of wild meat (in tonnes) consumed in the region in one year for each scenario *z* as

30 $$\mathrm{Total}\,\mathrm{tonnes}\,{\mathrm{consumed}}_{z}=\mathop{\sum }\limits_{j=1}^{J}\left(\frac{\mathrm{Tonnes}\,{\mathrm{consumed}}_{{j},{z}}}{1,000}\right)$$

Where total tonnes consumed is the number of tonnes consumed in the region in a year for scenario *z*, tonnes consumed is the result of equation (29) for cell *j* and 1,000 is the factor converting consumed kilograms to tonnes.

Finally, we mapped the consumption rates (Fig. 3a) and tonnes of wild meat consumed (Fig. 3c) in Central Africa using QGis (.3.22.1)^69^ by interpolating the values thus obtained using the plugin Heatmap, which returns a density layer using kernel density estimation weighed using the predicted values. For that, we used a radius of 0.9 decimal degrees and a Quartic kernel decay rate.

### Evaluating geographical uncertainty

As we predicted wild meat consumption over the entire Central African region, we wanted to evaluate the uncertainty of our estimates. To do so, we produced maps of uncertainty associated with our spatial estimates following two different approaches.

First, we extracted the s.d. of the posterior distribution of predicted values of each cell *j* (1) consumption rates (Extended Data Fig. 3b) and (2) biomass consumed (Extended Data Fig. 3c).

Second, we produced (3) a map of uncertainty based on the difference between the characteristics (that is, the continuous variables evaluated as potential drivers) of each cell of our prediction grid and the average values of our data. To do so, we first calculated the mean of the values of each continuous variable *V* (that is, human population density HPD, remoteness REM, human development index HDI and forest condition index FCI) assigned to each recall event *r*. By that, we obtained four average values ${\underline{M}}_{V}$, one for each variable *V*. Then, for each variable *V*, we subtracted ${\underline{M}}_{V}$ calculated from the data to the value *x* assigned to each cell *j* of our prediction grid. In doing so, we obtained a difference ${\delta }_{j}={x}_{j}-{\underline{M}}_{V}$, with 0 being equal to no difference between the average of the data and the value of the prediction cell. To standardize the difference, we converted negative values of ${\delta }_{j}$ to positive and then obtained the normalized differences ${\delta }_{j}^{{\prime} }$, with values of between 0 (that is, no difference) and 1 (maximum difference), one for each variable *V*. We then summed the values obtained in this manner for each prediction cell *j* and obtained an index of dissimilarity going from 0 (that is, no difference) to 4 (that is, maximum difference), as $\triangle j={\sum }_{j=1}^{J=874}{\delta }_{j}^{{\prime} }$.

Finally, we mapped the uncertainty values obtained in QGis (v.3.22.1)^69^ (Extended Data Fig. 4). As above we used the plugin Heatmap, with radius around points of 0.9 decimal degrees and a Quartic kernel decay rate.

### Compiling, running and checking the model

We coded our model in Stan^96^ and run it in R (v.4.2.0)^76^, using four chains in parallel for 5,000 (4,000 warmup) iterations each using RStan (v.2.26.11)^77^. We then evaluated the model convergence by examining the potential scale reduction factor Rhat of the estimated parameters (Extended Data Table 3) as well as the trace plots (Supplementary Fig. 15) of the realized iterations^87^. Finally, we ensured that the model did not show issues of collinearity by visually inspecting the pairs plots of the residuals of the explanatory variables^87^ (Supplementary Fig. 16).

### Reporting summary

Further information on research design is available in the Nature Portfolio Reporting Summary linked to this article.

## Online content

Any methods, additional references, Nature Portfolio reporting summaries, source data, extended data, supplementary information, acknowledgements, peer review information; details of author contributions and competing interests; and statements of data and code availability are available at 10.1038/s41586-026-10422-w.

## Supplementary information

Supplementary Information Supplementary Methods, Supplementary Results, Supplementary Discussion and Supplementary References.

Reporting Summary

## Figures and Tables

**Extended Data Table 1:**
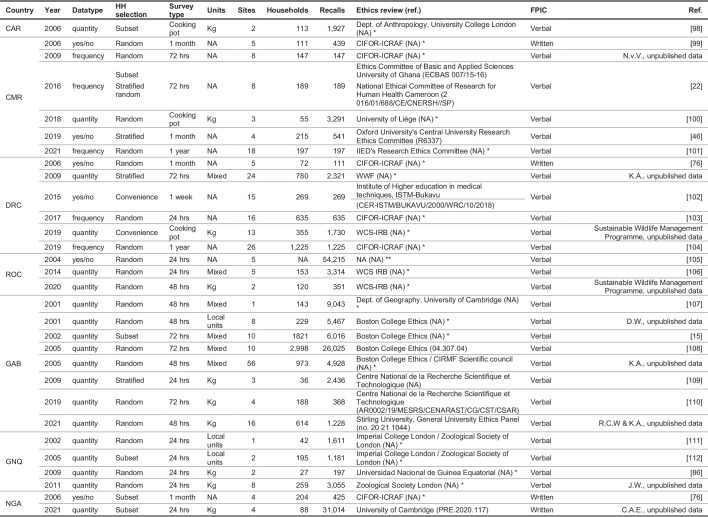
Studies included in the analysis

*Country* | Where the data were collected: CAR = Central Africa Republic; CMR = Cameroon; DRC = Democratic Republic of the Congo; ROC = Republic of Congo; GNQ = Equatorial Guinea; GAB = Gabon; NGA = Nigeria (Cross River State only). *Year* | When data were collected. *Survey type* | Data collection method: 24 h, 48 h, 72 h, 1 week, 1 month, 1 year = duration of recall period in recall studies; Cooking pot = cooking pot studies. *Datatype* | Type of data collected: yes/no = consumption / non-consumption; frequency = frequency of consumption; quantity: quantity consumed (grams, kilograms or local units). *Units* | Recorded units (only studies recording quantity consumed): Kg = kilograms or grams; Local = local units only; Mixed = kilograms or grams measured occasionally, if not measured local units were recorded. *Locations* | Number of locations (i.e., villages, towns, or cities) surveyed. *Households* | Number of monitored households. *Recalls* | Number of recall events monitored in the study. *Ethics Review (ref.)* | Institution (reference number) of ethics review of field protocols and research proposals. *FPIC* | Type of Free Prior Informed Consent obtained. *Ref*. | Bibliographic reference ^98–112^. | * ethics review of research proposals and field protocols obtained, but reference number was unavailable/not assigned. | ** Study conducted under the auspices of WCS-Congo, no ethics review body or reference number available at the time of the study.

**Extended Data Table 2:**
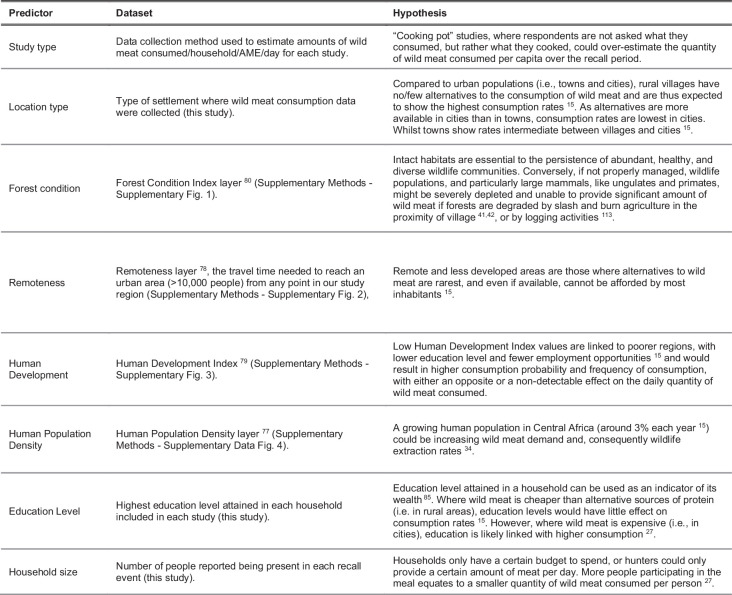
Correlates of wild meat consumption considered in the analysis

*Predictor |* Name of variables included in the analysis. *Dataset |* Data type and source*. Hypothesis |* Expected result and reasons for inclusion in the analysis. For more details, see Methods, “Correlates of wild meat consumption”^113^.

**Extended Data Table 3:**
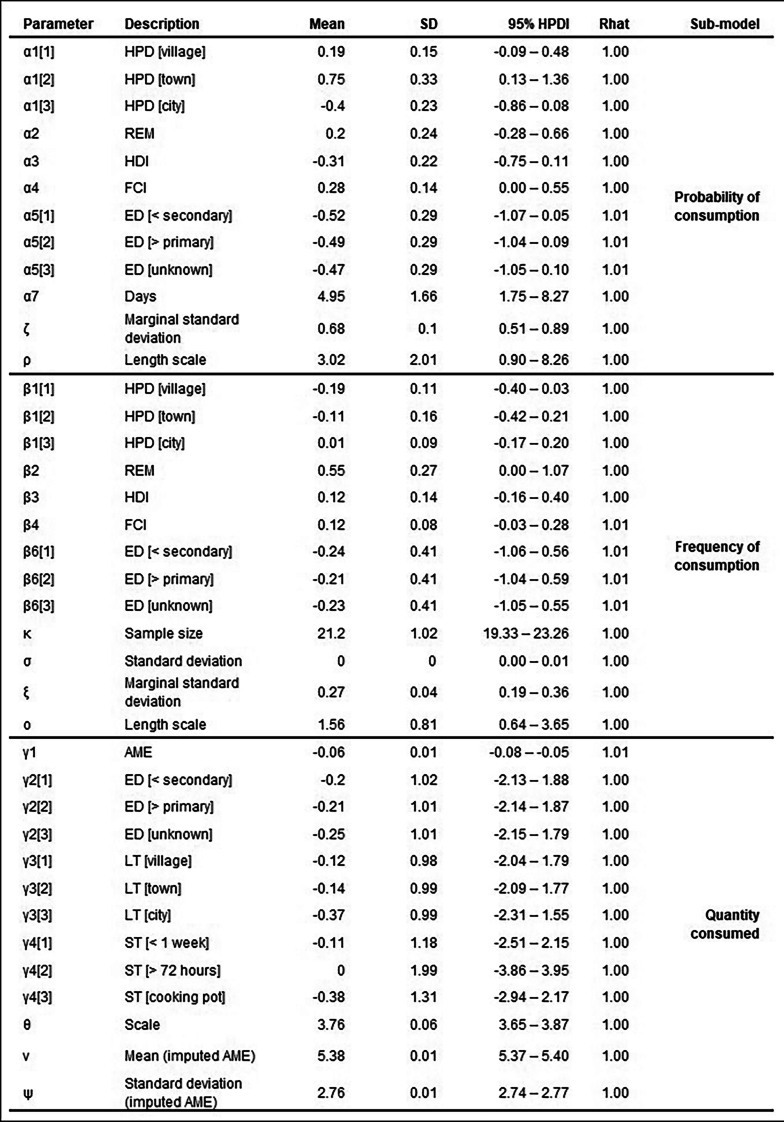
Model full results

*Parameter* | Parameters estimated by the model (Extended Data Figs. 1a–3). *Description* | Parameter explanation (cfr. Table 3 and Extended Data Figs. 1a–3). *Mean* | Estimated mean value. *SD* | Standard deviation. *95% HPDI* | 95% Highest Posterior Density Intervals. *Rhat* | Scale reduction factor measuring convergence. *Sub-model* | Sub-model where the parameter is estimated^15,40,41,82–84^.

## Data Availability

Our study uses raw data from studies conducted from the year 2000 until 2022 and therefore did not generate new data. Wild-meat consumption data were extracted from different published and unpublished sources as described in Extended Data Table 1. Owing to the sensitive nature of the data (including illegal activities, such as the consumption of protected wildlife species), unprocessed datasets are available with restrictions through the WILDMEAT Data Portal (https://explorer.wildmeat.org/). Each dataset is available under different data sharing conditions through a data user agreement, which gives data users control over the distribution and use of their data. The full processed dataset used for analysis will be shared on request with researchers seeking to replicate the study results. Other requests must clearly specify the study objectives, and access will be granted on a case-by-case basis after obtaining permission from the original data providers. In all cases, data recipients will be required to abide by the data-sharing agreements of WILDMEAT. All requests should be addressed to the corresponding author. The data needed to reproduce the figures and maps shown in the main text are available at Zenodo^97^ (10.5281/zenodo.19021125). The spatial layers used in our analysis are described in Extended Data Table 2 and are available at https://www.forestintegrity.com/ (forest condition index, under a CC BY 4.0 licence), https://human-settlement.emergency.copernicus.eu/ghs_pop2019.php/ (human population density, under a CC BY 4.0 licence), https://human-settlement.emergency.copernicus.eu/ghs_smod2023.php (settlement type, under a CC BY 4.0 licence), https://malariaatlas.org (remoteness, a under CC BY 3.0 licence), https://globaldatalab.org/ (subnational human development index), https://dhsprogram.com and https://mics.unicef.org (education level). Forest blocks shown in Fig. 1 are available at https://data.mendeley.com/datasets/7gskp92yx6/1 under a CC BY 4.0 licence.

## Code availability

R and Stan code used to conduct our analyses is freely available at GitHub (https://github.com/mattiabessone/Wild-animal-consumption-is-increasing-in-Central-Africa).
