# Supplementary material for: Increase in wild animal consumption across Central Africa
Source: Nature. 2026 Apr 29;653(8116):1092–8. doi: 10.1038/s41586-026-10422-w (PMC13216070; doi:10.1038/s41586-026-10422-w)
Supplement: Supplementary file 1 — Supplementary Methods, Supplementary Results, Supplementary Discussion and Supplementary References. [file 41586_2026_10422_MOESM1_ESM.pdf]

nature portfolio

https://doi.org/10.1038/s41586-026-10422-w

Supplementary information

Increase in wild animal consumption across Central Africa

In the format provided by the authors and unedited

Increase in wild animal consumption across Central Africa

Mattia Bessone [1,2,3*], Daniel J. Ingram [4,5], Katharine Abernethy [5,6], Sylvanus Abua [1], Sophie Allebone-Webb [7,8], Daniela Antonacci [9], Riyong Kim [1,10], Stephanie Brittain [4,11], Daniel Cornelis [12], Diane Detoeuf [9], Charles A. Emogor [9,13], Julia E. Fa [1,14,15], Steffen Foerster [16], Davy Fonteyn [12], Maria Grande Vega [17,18], Chloe Hodgkinson [19,20], Amy Ickowitz [1], Cédric Thibaut Kamogne Tagne [21,22], Della Kemalasari [1], Noëlle Kümpel [7,8,23], Simon Lhoest [24], Germain Mavah [9], Rodrigue Guy Mouanda Niamba [9], Donald Midoko Iponga [6], Eleanor J. Milner-Gulland [11,25], Jonas Muhindo [1,26], Théodore Munyuli [27,28], Robert Nasi [1], Steeve Ngama [24,29], Jonas Nyumu [1,26,30], Justin Ombeni [27,31], John R. Poulsen [32,33], Dominic Rowland [1], Yahya Sampurna [1], François Sandrin [9], Malcolm Starkey [34,35], Caleb Tata [36], Julius C. Tieguhong [1,37], Nathalie van Vliet [1], Philippe Vigneron [12], Robin C. Whytock [5,38], Michelle Wieland [9], David Wilkie [9], Jasmin Willis [1,11], Juliet Wright [7,9,11] & Lauren Coad [1,11]

Affiliations

[1] Centre for International Forestry Research, CIFOR, Kota Bogor, Indonesia; [2] Department for the Ecology of Animal Societies, Max Planck Institute of Animal Behavior, Konstanz, Germany; [3] Centre for the Advanced Study of Collective Behaviour, University of Konstanz, Konstanz, Germany; [4] Durrell Institute of Conservation and Ecology (DICE), School of Natural Sciences, University of Kent, Canterbury, UK; [5] Faculty of Natural Sciences, University of Stirling, Stirling, UK; [6] Institute for Tropical Ecology Research (IRET), CENAREST, Libreville, Gabon; [7] Zoological Society of London, London, UK; [8] Centre for Environmental Policy & Department of Life Sciences, Imperial College London, Ascot, UK; [9] Wildlife Conservation Society, New York, USA; [10] Centre for Forest and Landscape, University of Copenhagen, Frederiksberg C., Denmark; [11] Interdisciplinary Centre for Conservation Science (ICCS), Department of Biology, University of Oxford, Oxford, UK; [12] Forêts et Sociétés, Université de Montpellier, CIRAD, Montpellier, France; [13] Department of Zoology, University of Cambridge, Cambridge, UK; [14] Department of Natural Sciences, Manchester Metropolitan University, Manchester, UK; [15] Natural Sciences and Environment Hub, University of Gibraltar, Campus Europa Point, Gibraltar

[16] Department of Evolutionary Anthropology, Duke University, Durham, USA; [17] Research Group SILVANET, College of Forestry and Natural Environment, Universidad Politécnica de Madrid, Madrid, Spain; [18] Asociación Ecotono, Madrid, Spain; [19] Fauna & Flora, Cambridge, UK; [20] University College London, London, UK; [21] Collective Action to Save the Environment (CASE), Yaoundé, Cameroon; [22] BAY-SUP, The Higher Institute of Environmental Sciences, Yaoundé, Cameroon; [23] BirdLife International, Cambridge, UK; [24] Gembloux Agro-Bio Tech, Université de Liège, Gembloux, Belgium; [25] Oxford Martin School, University of Oxford, Oxford, UK; [26] Solutions for Wildlife (SO WILD), Kisangani, DRC; [27] Department of Nutrition and Dietetics, Institut Supérieur de Techniques Médicales ISTM, Bukavu, DRC; [28] Laboratory of Entomology, Centre de Recherche en Sciences Naturelles CRSN-LWIRO, Bukavu, DRC; [29] Wildlife and Sustainable Development Research Program, IRAF-CENAREST, Libreville, Gabon; [30] University of Kisangani (UNIKIS), Kisangani, DRC; [31] Laboratory of Functional and Applied Entomology, LENAF, Institut Facultaire des Sciences Agronomiques IFA, Kisangani, DRC; [32] Nicholas School of the Environment, Duke University, Durham, USA; [33] The Nature Conservancy, Spruce St. Boulder, CO, USA; [34] The Biodiversity Consultancy, Cambridge, UK; [35] Department of Geography, University of Cambridge, Cambridge, UK; [36] Forests, Resources and People (FOREP), Botanic Gardens Limbe, Limbe, Cameroon; [37] African Natural Resources Management and Investment Centre, African Development Bank, Abidjan, Ivory Coast; [38] Okala, Stirling, UK

** Corresponding author: mattia.bessone@gmail.com*

Supplementary Methods

Supplementary Fig. 1: Grid used for predicting wild meat consumption rates and total biomass consumed. Each cell has an area of 5,027 km^2 (70,09 * 70,09 km), equal to the area of a circle of radius = 40 km. The latter represents the furthest distance that hunters in Central Africa are willing to cover to procure wild meat [40]. *Background:* green areas represent forest cover [122] and black areas depict large urban centres [33]; *Black circles:* surveyed locations; *Grey squares*: regional prediction grid; *Red squares*: Central African forest region prediction grid. This area was selected by sub-setting the regional prediction grid by selecting only cells intersecting a buffer (dotted black line) drawn around patches of continuous forest [32] (>5,000 km^2). To include areas of forest-savannah transition, we set a buffer radius equal to twice the side of the cells (i.e., 140.18 km). Credit: country outlines, geoportal.icpac.net under an Open Database License ODbL 1.0; map created with QGis 3.22.1 [83].

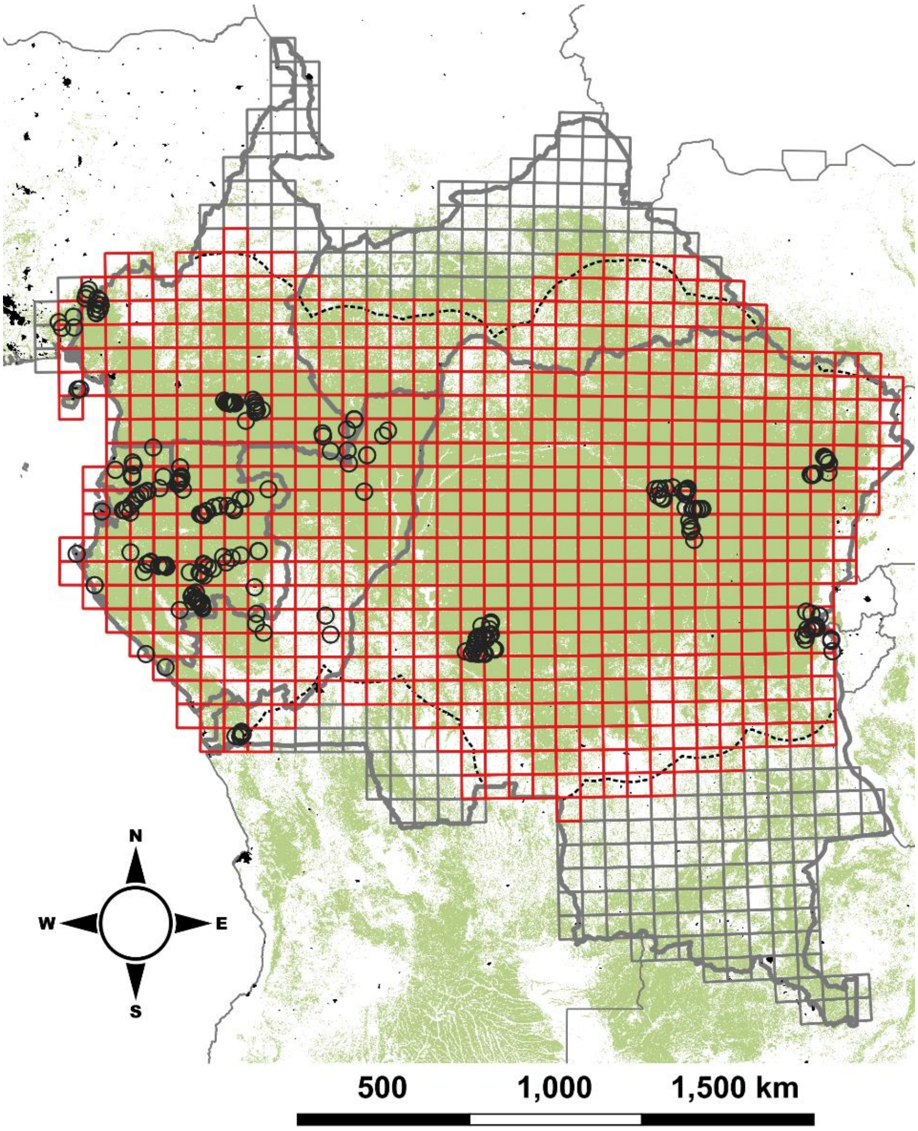

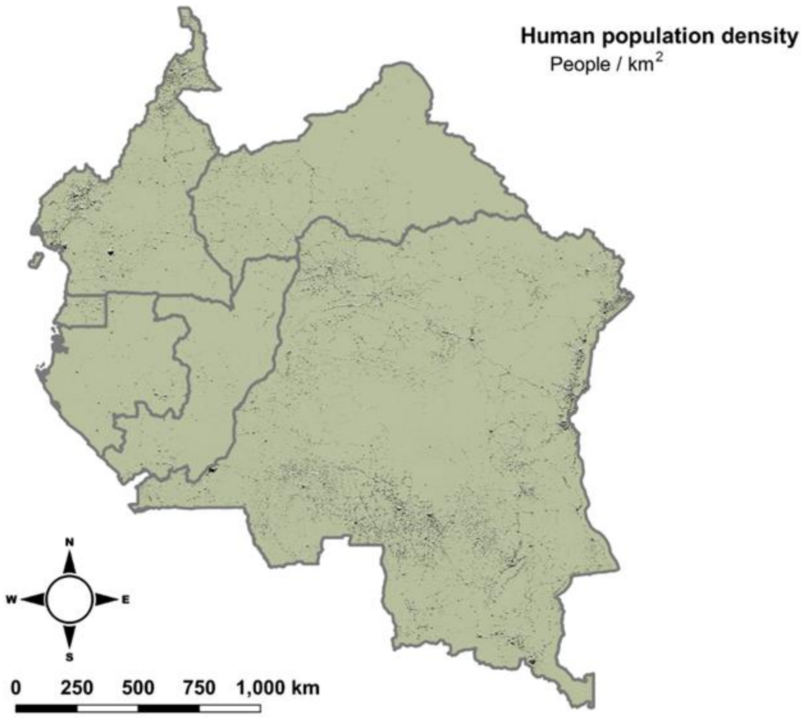

Supplementary Fig. 2: Human Population.
Hillshade map of human population for the year 2020 (data also available for 2000, 2005, 2010 and 2015) [77]. Darker areas represent larger concentration of people. *Credit*: country outlines, geoportal.icpac.net under an Open Database License ODbL 1.0; map created with QGis 3.22.1 [83].

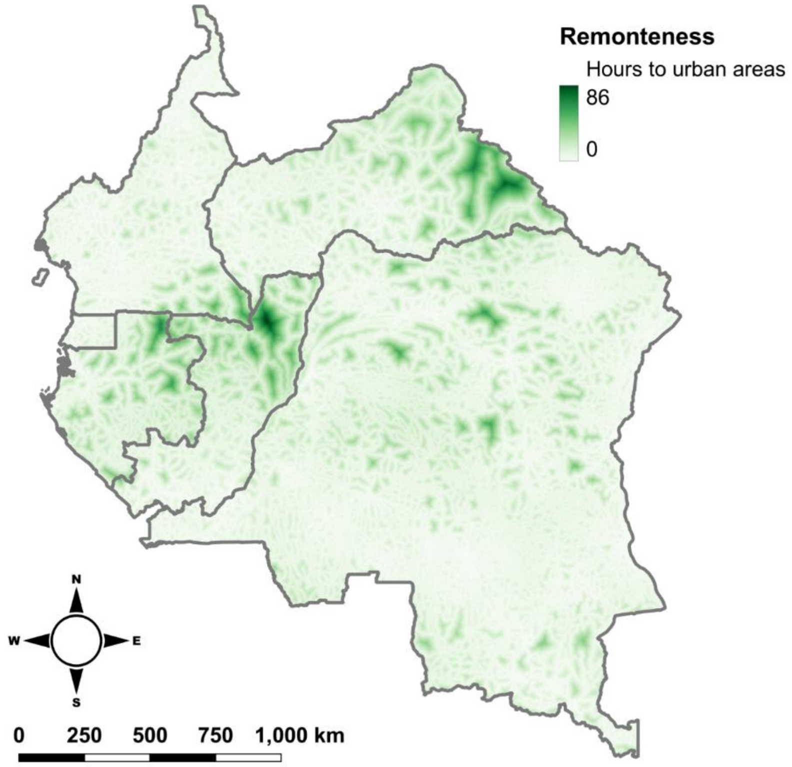

Supplementary Fig. 3: Remoteness.
Geographical variation in the travel time (in hours) required to reach an urban centre in 2015 [78].
Credit: country outlines, geoportal.icpac.net under an Open Database License ODbL 1.0; map created with QGis 3.22.1 [83].

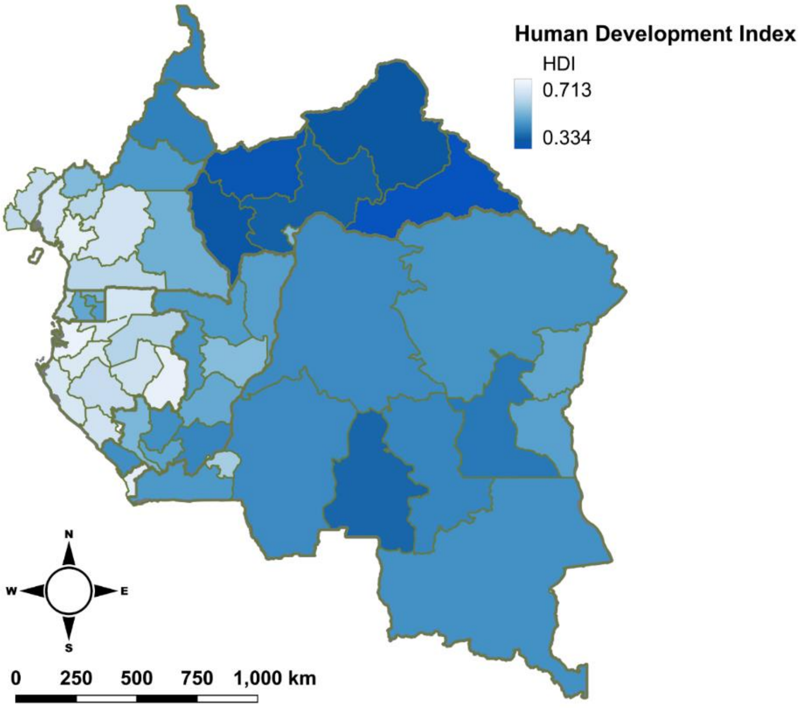

Supplementary Fig. 4: Subnational Human Development Index. Human development index values of each administrative level 1 in Central Africa in 2019 (data available continuously from 2000 to 2019) [79]. *Credit*: country outlines, geoportal.icpac.net under an Open Database License ODbL1.0; map created with QGis 3.22.1 [83].

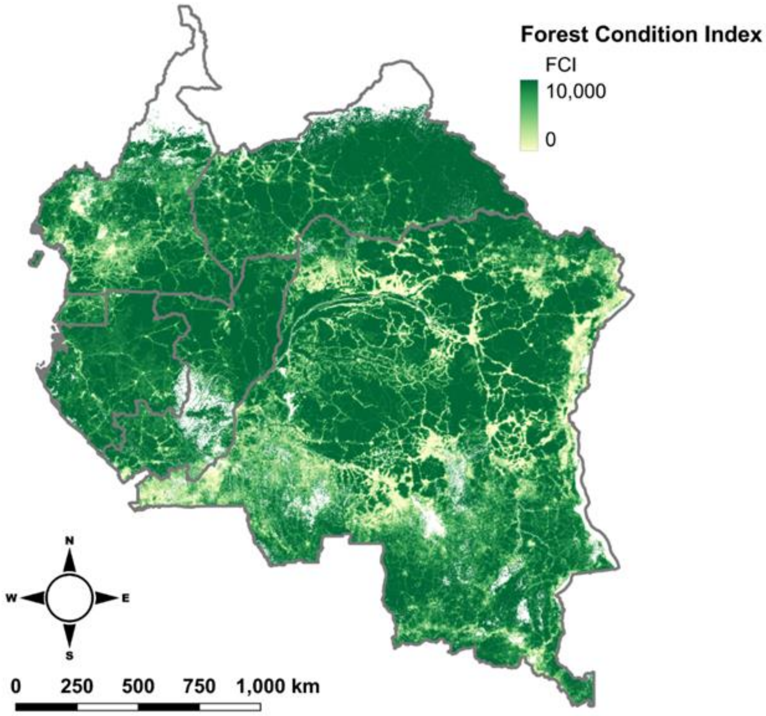

Supplementary Fig. 5: Forest Condition Index. Geographical distribution and condition of forested areas of Central Africa on a scale from 0 (= highly degraded) to 10,000 (= no degradation), in 2019 [80]. *Credit*: country outlines, geoportal.icpac.net under an Open Database License ODbL 1.0; map created with QGis 3.22.1 [83].

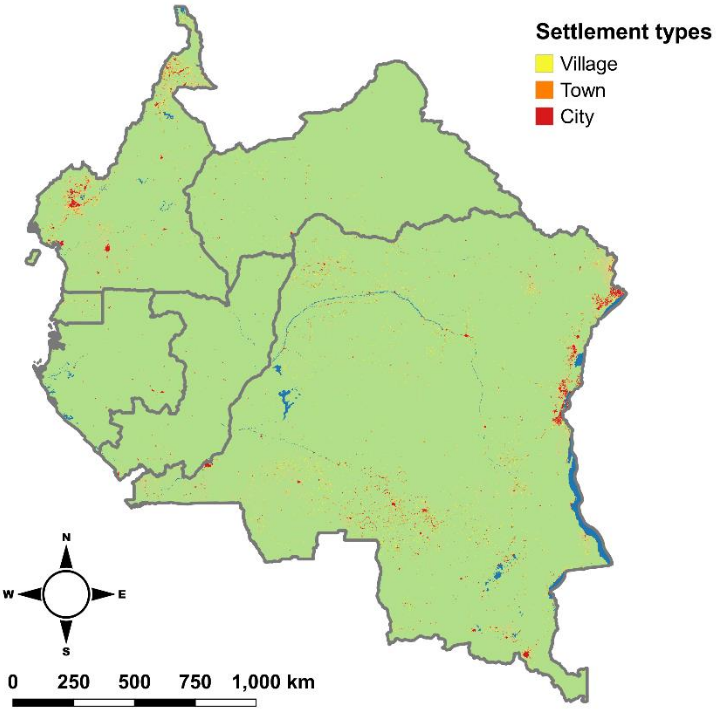

Supplementary Fig. 6: Settlement type.
Settlement types in Central Africa for the year 2020 (data also available for 2000, 2005, 2010 and 2015) [33].
Credit: country outlines, geoportal.icpac.net under an Open Database License ODbL 1.0; map created with QGis 3.22.1 [83].

Supplementary Fig. 7: Subnational proportion of people attaining secondary education. Proportion of people who declared having a secondary education (or higher) in each administrative level 1 in Central Africa for the period 2011-2022, based on 6 ICF Demographic Health Surveys "DHS" (dhsprogram.com) 6 and UNICEF Multiple Indicator Cluster Surveys "MICS" (mics.unicef.org), including information on 112,642 households. Data for Equatorial Guinea are derived from the only survey available (Supplementary Table 4), including information from 2,640 households.
Credit: country outlines, geoportal.icpac.net under an Open Database License ODbL 1.0; map created with QGis 3.22.1 [83].

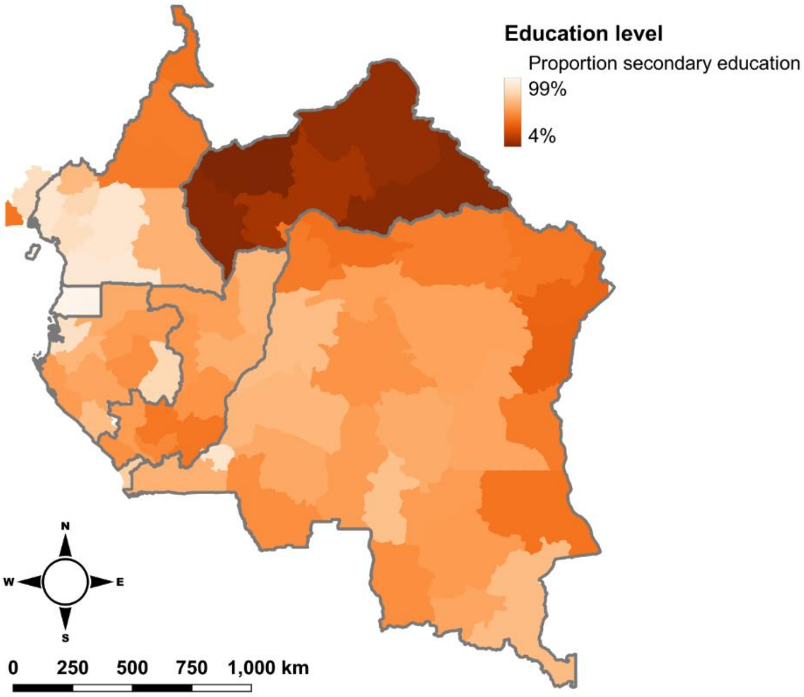

Supplementary Table 1: List of priors used for the parameters estimated in the models.

Parameter	Description	Prior	Sub-model
α0	Intercept	$Normal \sim (0, 1.4)$	Probability of consumption
α1…k	Slope – continuous variables	$Normal \sim (0, 0.5)$	
	Factor – categorical/random variables	$Normal \sim (0, 1.4)$	
κ	Sample size – Beta distribution	$Exponential \sim (1)$	Frequency of consumption
σ	Standard deviation	$Exponential \sim (5)$	
β0	Intercept	$Normal \sim (0, 1.4)$	
β1…k	Slope – continuous variables	$Normal \sim (0, 0.5)$	
	Factor – categorical/random variables	$Normal \sim (0, 1.4)$	
τ	Non-centered parameterization	$Normal \sim (0, 1)$	
θ	Scale – Gamma distribution	$Exponential \sim (1)$	Quantity consumed
γ0	Intercept	$Normal \sim (0, 5)$	
γ1…k	Slope – continuous variables	$Normal \sim (0, 0.5)$	
	Factor – categorical/random variables	$Normal \sim (0, 5)$	
ζ	Marginal standard deviation	$Normal \sim (0, 1)$	Spatial autocorrelation
ρ	Length scale	$Inverse\ Gamma \sim (5, 5)$	
η	Generates the latent gaussian process	$Normal \sim (0, 1)$	
ν	Mean	$Normal \sim (\underline{AME}, 1)$	AME imputation
ψ	Standard deviation	$Exponential \sim (1)$	

Supplementary Table 2: Values assigned to each parameter estimated in the simulation study.

Parameter	Value	Description	Sub-model
	0.80		
α0	-3.50	Intercept varying by region	
	-1.00		
	0.40		
α1	0.30	Slope (*V1*), varying by region	Probability of consumption
	0.10		
	2.00		
α2	0.30	Random factor (*V3*)	
ζ	0.80	Marginal standard deviation	
ρ	2.00	Length scale	
κ	15.00	Sample size parameter	
σ	0.03	Standard deviation	
	0.90		
$\beta 0$	-0.50	Intercept varying by region	Frequency of consumption
	0.50		
	0.15		
$\beta 1$	0.20	Slope (*V2*), varying by region	
	0.08		
	1.00		
$\beta 2$	-0.90	Random factor (*V3*)	
Θ	10.00	Scale parameter	
	5.80		
$\gamma 0$	5.00	Intercept varying by region	
	5.50		Quantity consumed
	0.01		
$\gamma 1$	0.06	Slope (*V2*), varying by region	
	-0.02		
$\gamma 2$	-0.07	Slope (AME)	

Supplementary Table 3: Continuous, categorical, and random variables included in the models. * Studies included in this analysis.

Level	Name	Abbreviation	Index	Type	Description	Source
Study	Study ID	*S*	*s*	Random	30 levels.<br>One for each study included in the analysis.	*
	Study type	*ST*	*st*	Categorical	3 levels.<br>**1**: 24hrs; 48hrs; 72hrs; **2**: week; month; year; **3**: cooking-pot	
Site	Location ID	*L*	*l*	Random	252 levels.<br>One for each location included in the analysis.	*
	Location type	*LT*	*lt*	Categorical	3 levels.<br>**1**: Village (< 10,000 inhabitants); **2**: Town (>10,000 and <100,000); **3**: City (>100,000)	
	Distance between locations	*D*	/	Continuous	Euclidean distances between locations.	
	Human population density	*HPD*	/	Continuous	Human population density, i.e. people/km^2 (mean within 40km around site location).<br>Year of data: 2000; 2005; 2010; 2015; 2020<br>Resolution: 100m<br>Range: 0.0 – 492.7	[77]
	Remoteness	*REM*	/	Continuous	Remoteness, i.e. travel time in minutes, to urban areas between >10,000 and <100,000 people (mean within 40km around site location).<br>Year of data: 2015<br>Resolution: 1km<br>Range: 172.9 – 4,070.9	[78]
	Human Development Index	*HDI*	/	Continuous	Subnational Human Development Index.<br>Year of data: continuous from 2000-2019<br>Resolution: Administrative level 1<br>Range: 0.31 – 0.74	[79]
	Forest condition index	*FCI*	/	Continuous	Continuous index of forest condition, determined by anthropogenic modification (mean within 40km around site location).<br>Year of data: 2019<br>Resolution: 300m<br>Range: 1,367.9 – 9,644.7	[80]
Household	Household ID	*HH*	*h*	Random	66,580 levels.<br>One for each household included in the analysis.	*
	Education level	*ED*	*ed*	Categorical	7 levels.<br>**1**: =< primary in villages; **2**: =< primary in towns; **3**: =< primary in cities; **4**: >primary in villages; **5**: >primary in towns; **6**: >primary in cities; **7**: unknown	
Recall	Recall ID	*R*	*r*	Random	163,896 levels.<br>One for each recall event included in the analysis.	*
	Adult Male Equivalent	*AME*	/	Continuous	Number of AME present in the household during a recall event.	

Supplementary Table 4: Surveys providing the subnational proportion of secondary school attendance. *Country* | Where the data were collected: CAF = Central Africa Republic; CMR = Cameroon; COD = Democratic Republic of the Congo; COG = Republic of Congo; GNQ = Equatorial Guinea; GAB = Gabon; NGA = Nigeria (Cross River State only). *Year* | When data were collected. *Households* | Number of monitored households. *Survey* | DHS = ICF Demographic Health Surveys (https://dhsprogram.com/methodology/survey-Types/dHs.cfm); MICS = UNICEF Multiple Indicator Cluster Surveys "MICS" (https://mics.unicef.org/).

Country	Year	Households	Survey
CAF	2006	11622	MICS
	2010	11725	MICS
	2018	8107	MICS
CMR	2000	2936	MICS
	2004	10459	DHS
	2006	9624	MICS
	2011	13993	DHS
	2015	10199	MICS
	2018	11708	DHS
COD	2001	11302	MICS
	2007	8882	DHS
	2010	11302	MICS
	2014	18170	DHS
	2018	20785	MICS
COG	2005	5877	DHS
	2009	6086	DHS
	2012	11615	DHS
	2015	12776	MICS
GAB	2000	6039	DHS
	2012	1749	DHS
GNQ	2000	2640	MICS
NGA	2007	1357	MICS
	2011	1585	MICS
	2017	1808	MICS
	2021	147	DHS

Supplementary Results

Supplementary Fig. 8: Estimated effect (0 = no effect) of the interaction between Human Population Density (HPD) and settlement type on wild meat consumption probability (blue) and frequency of consumption (pink). Estimates are broken down by settlement type. *Coloured clouds of dots*: posterior distribution of the effect estimated by the model (n = 4,000 posterior draws). *Coloured diamonds*: mean of the posterior distribution; Solid bars: 95% Highest Posterior Density Intervals.

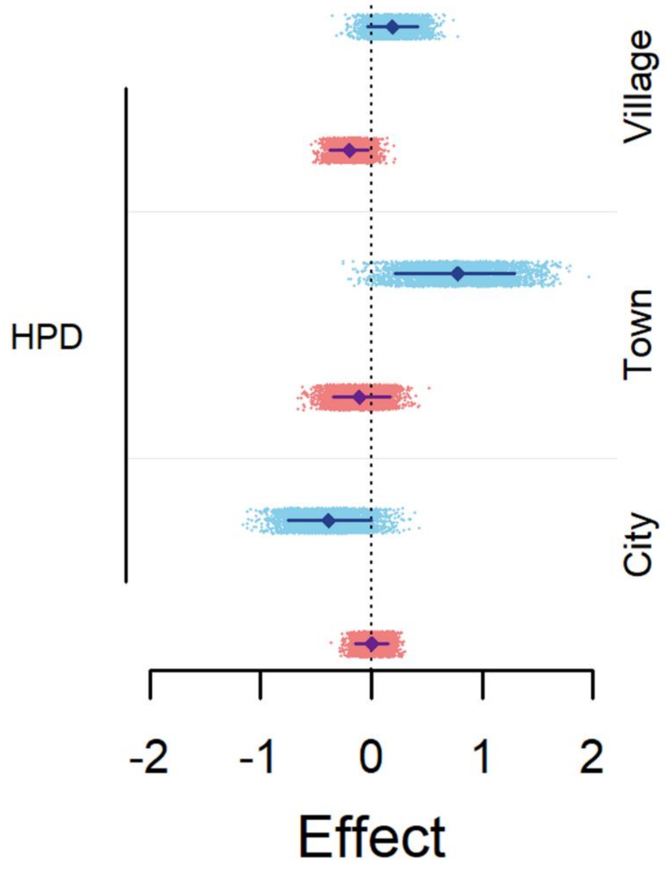

Supplementary Table 5: Results of the model investigating the interaction between education level and settlement type. *Parameter* | Parameters estimated by the model (Extended data Equation 1-3). *Description* | Parameter explanation (cfr. Table 3 and Extended data Equation 1-3). *Mean* | Estimated mean value. *SD* | Standard deviation. *95% HPDI* | 95% Highest Posterior Density Intervals. *Rhat* | Scale reduction factor measuring convergence. *Sub-model* | Sub-model where the parameter is estimated.

Parameter	Description	Mean	SD	95% HPDI	Rhat	Sub-model
α1[1]	*HPD* [village]	0.19	0.15	-0.09 – 0.48	1.00	
α1[2]	*HPD* [town]	0.75	0.33	0.13 – 1.36	1.00	
α1[3]	*HPD* [city]	-0.40	0.23	-0.86 – 0.08	1.00	
α2	*REM*	0.20	0.24	-0.28 – 0.66	1.00	
α3	*HDI*	-0.31	0.22	-0.75 – 0.11	1.00	
α4	*FCI*	0.28	0.14	0.00 – 0.55	1.00	
α5[1]	*ED* [=< primary, village]	-0.63	0.36	-1.39 – 0.03	1.01	
α5[2]	*ED* [>= secondary, village]	-0.60	0.36	-1.36 – 0.06	1.01	**Probability of consumption**
α5[3]	*ED* [=< primary, town]	-0.59	0.36	-1.36 – 0.07	1.01	
α5[1]	*ED* [>= secondary, town]	-0.02	1.41	-2.82 – 2.65	1.00	
α5[2]	*ED* [=< primary, city]	-0.04	1.42	-2.79 – 2.72	1.00	
α5[3]	*ED* [>= secondary, city]	0.01	1.38	-2.68 – 2.69	1.00	
α5[3]	*ED* [unknown]	-0.02	1.39	-2.73 – 2.66	1.00	
α7	*Days*	4.95	1.66	1.75 – 8.27	1.00	
ζ	Marginal standard deviation	0.68	0.10	0.51 – 0.89	1.00	
ρ	Length scale	3.02	2.01	0.90 – 8.26	1.00	
β1[1]	*HPD* [village]	-0.19	0.11	-0.40 – 0.03	1.00	
β1[2]	*HPD* [town]	-0.11	0.16	-0.42 – 0.21	1.00	
β1[3]	*HPD* [city]	0.01	0.09	-0.17 – 0.20	1.00	
β2	REM	0.55	0.27	0.00 – 1.07	1.00	
β3	*HDI*	0.12	0.14	-0.16 – 0.40	1.00	
β4	*FCI*	0.12	0.08	-0.03 – 0.28	1.01	
β5[1]	*ED* [=< primary, village]	-0.26	0.41	-1.06 – 0.54	1.00	
β5[2]	*ED* [>= secondary, village]	-0.22	0.41	-1.02 – 0.58	1.00	
β5[3]	*ED* [=< primary, town]	-0.25	0.41	-1.05 – 0.55	1.00	**Frequency of consumption**
β5[4]	*ED* [>= secondary, town]	-0.02	1.40	-2.80 – 2.79	1.00	
β5[5]	*ED* [=< primary, city]	0.00	1.44	-2.76 – 2.84	1.00	
β5[6]	*ED* [>= secondary, city]	0.03	1.40	-2.72 – 2.69	1.00	
β5[7]	*ED* [unknown]	-0.01	1.39	-2.69 – 2.74	1.00	
κ	Sample size	21.20	1.02	19.33 – 23.26	1.00	
σ	Standard deviation	0.00	0.00	0.00 – 0.01	1.00	
ξ	Marginal standard deviation	0.27	0.04	0.19 – 0.36	1.00	
ο	Length scale	1.56	0.81	0.64 – 3.65	1.00	
γ1	*AME*	-0.06	0.01	-0.08 – -0.05	1.01	
γ2[1]	*ED* [=< primary, village]	-0.14	1.04	-2.13 – 1.90	1.00	
γ2[2]	*ED* [>= secondary, village]	-0.16	1.04	-2.17 – 1.89	1.01	
γ2[3]	*ED* [=< primary, town]	-0.20	1.05	-2.21 – 1.84	1.00	
γ2[4]	*ED* [>= secondary, town]	-0.01	2.00	-3.89 – 3.78	1.00	
γ2[5]	*ED* [=< primary, city]	-0.01	2.06	-3.99 – 4.12	1.00	
γ2[6]	*ED* [>= secondary, city]	0.00	2.00	-3.82 – 4.02	1.00	
γ2[7]	*ED* [unknown]	-0.01	2.04	-3.97 – 3.92	1.00	
γ3[1]	*LT* [village]	-0.12	0.98	-2.04 – 1.79	1.00	**Quantity consumed**
γ3[2]	*LT* [town]	-0.14	0.99	-2.09 – 1.77	1.00	
γ3[3]	*LT* [city]	-0.37	0.99	-2.31 – 1.55	1.00	
γ4[1]	*ST* [=< 72 hours]	-0.11	1.18	-2.51 – 2.15	1.00	
γ4[2]	*ST* [> 72 hours]	0.00	1.99	-3.86 – 3.95	1.00	
γ4[3]	*ST* [cooking pot]	-0.38	1.31	-2.94 – 2.17	1.00	
θ	Scale	3.76	0.06	3.65 – 3.87	1.00	
ν	Mean (imputed *AME*)	5.38	0.01	5.37 – 5.40	1.00	
ψ	Standard deviation (imputed *AME*)	2.76	0.01	2.74 – 2.77	1.00	

Parameters with more than seven levels (e.g., intercepts [n = 30] and random factors [n = 66,580]) are not shown.

Supplementary Table 6: Results of the model including only two settlement types (rural and urban). *Parameter* | Parameters estimated by the model (Extended data Equation 1-3). *Description* | Parameter explanation (cfr. Supplementary Table 3 and Extended data Equation 1-3). *Mean* | Estimated mean value. *SD* | Standard deviation. *95% HPDI* | 95% Highest Posterior Density Intervals. *Rhat* | Scale reduction factor measuring convergence. *Sub-model* | Sub-model where the parameter is estimated.

Parameter	Description	Mean	SD	95% HPDI	Rhat	Sub-model
α1[1]	*HPD* [rural]	0.16	0.15	-0.13 – 0.44	1.00	**Probability of consumption**
α1[2]	*HPD* [urban]	-0.02	0.21	-0.42 – 0.40	1.00	
α2	*REM*	0.19	0.25	-0.31 – 0.67	1.01	
α3	*HDI*	-0.30	0.23	-0.75 – 0.14	1.01	
α4	*FCI*	0.32	0.14	0.04 – 0.60	1.00	
α5[1]	*ED* [=< primary]	-0.63	0.34	-1.32 – 0.00	1.01	
α5[2]	*ED* [>= secondary]	-0.61	0.34	-1.32 – 0.04	1.01	
α5[3]	*ED* [unknown]	-0.59	0.34	-1.29 – 0.04	1.01	
α7	*Days*	0.26	0.22	0.01 – 0.85	1.00	
ζ	Marginal standard deviation	0.70	0.10	0.53 – 0.91	1.00	
ρ	Length scale	3.36	2.40	0.93 – 9.71	1.00	
β1[1]	*HPD* [rural]	-0.18	0.11	-0.4 – 0.04	1.00	**Frequency of consumption**
β1[2]	*HPD* [urban]	-0.02	0.09	-0.19 – 0.15	1.01	
β2	REM	0.54	0.27	0.01 – 1.06	1.01	
β3	*HDI*	0.11	0.15	-0.17 – 0.41	1.01	
β4	*FCI*	0.11	0.07	-0.04 – 0.25	1.00	
β5[1]	*ED* [=< primary]	-0.23	0.42	-1.03 – 0.59	1.01	
β5[2]	*ED* [>= secondary]	-0.19	0.42	-1.01 – 0.63	1.01	
β5[3]	*ED* [unknown]	-0.22	0.42	-1.03 – 0.60	1.01	
κ	Sample size	20.90	1.01	18.98 – 22.96	1.00	
σ	Standard deviation	0.00	0.00	0.00 – 0.01	1.00	
ξ	Marginal standard deviation	0.27	0.04	0.20 – 0.35	0.00	
ο	Length scale	1.53	0.79	0.62 – 3.49	1.00	
γ1	*AME*	-0.06	0.01	-0.07 – -0.05	1.01	**Quantity consumed**
γ2[1]	*ED* [=< primary]	-0.10	0.99	-2.00 – 1.91	1.00	
γ2[2]	*ED* [>= secondary]	-0.11	0.99	-2.01 – 1.88	1.00	
γ2[3]	*ED* [unknown]	-0.16	0.99	-2.04 – 1.81	1.00	
γ3[1]	*LT* [rural]	-0.26	1.14	-2.44 – 1.94	1.00	
γ3[2]	*LT* [urban]	-0.33	1.15	-2.56 – 1.89	1.00	
γ4[1]	*ST* [=< 72 hours]	-0.07	1.24	-2.53 – 2.38	1.00	
γ4[2]	*ST* [> 72 hours]	0.05	1.99	-3.79 – 3.93	1.00	
γ4[3]	*ST* [cooking pot]	-0.38	1.34	-2.98 – 2.18	1.00	
θ	Scale	3.76	0.05	3.66 – 3.87	1.00	
ν	Mean (imputed *AME*)	5.38	0.01	5.36 – 5.40	1.00	
ψ	Standard deviation (imputed *AME*)	2.76	0.01	2.74 – 2.77	1.00	

Parameters with more than seven levels (e.g., intercepts [n = 30] and random factors [n = 66,580]) are not shown.

Simulation study

Our model correctly estimated the total amount of wild meat consumed in the simulated study area, with precision increasing with increasing coverage (Supplementary Fig. 8). The model also successfully estimated the effect of the continuous (Supplementary Fig. 9) and categorical variables (Supplementary Fig. 10) on consumption probability, frequency of consumption and quantity consumed.

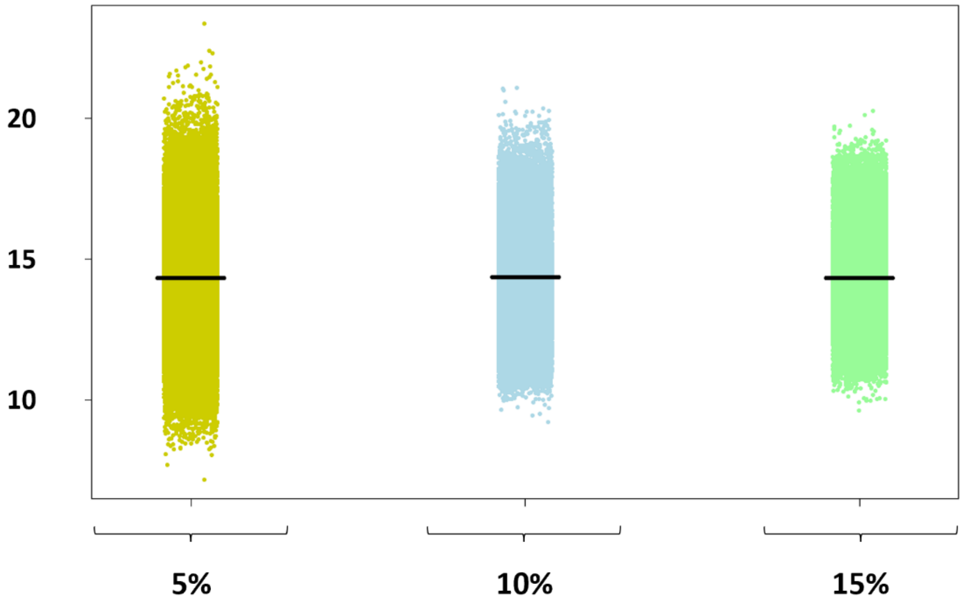

Supplementary Fig. 9: Simulation results – Tonnes of wild meat consumed. Posterior distribution (100,000 draws) of estimated number of tonnes of wild meat consumed resulting from 100 generated datasets for 3 different scenarios of survey coverage: 5% (yellow), 10% (blue) 15% (green). The real simulated values are represented by the black horizontal lines.

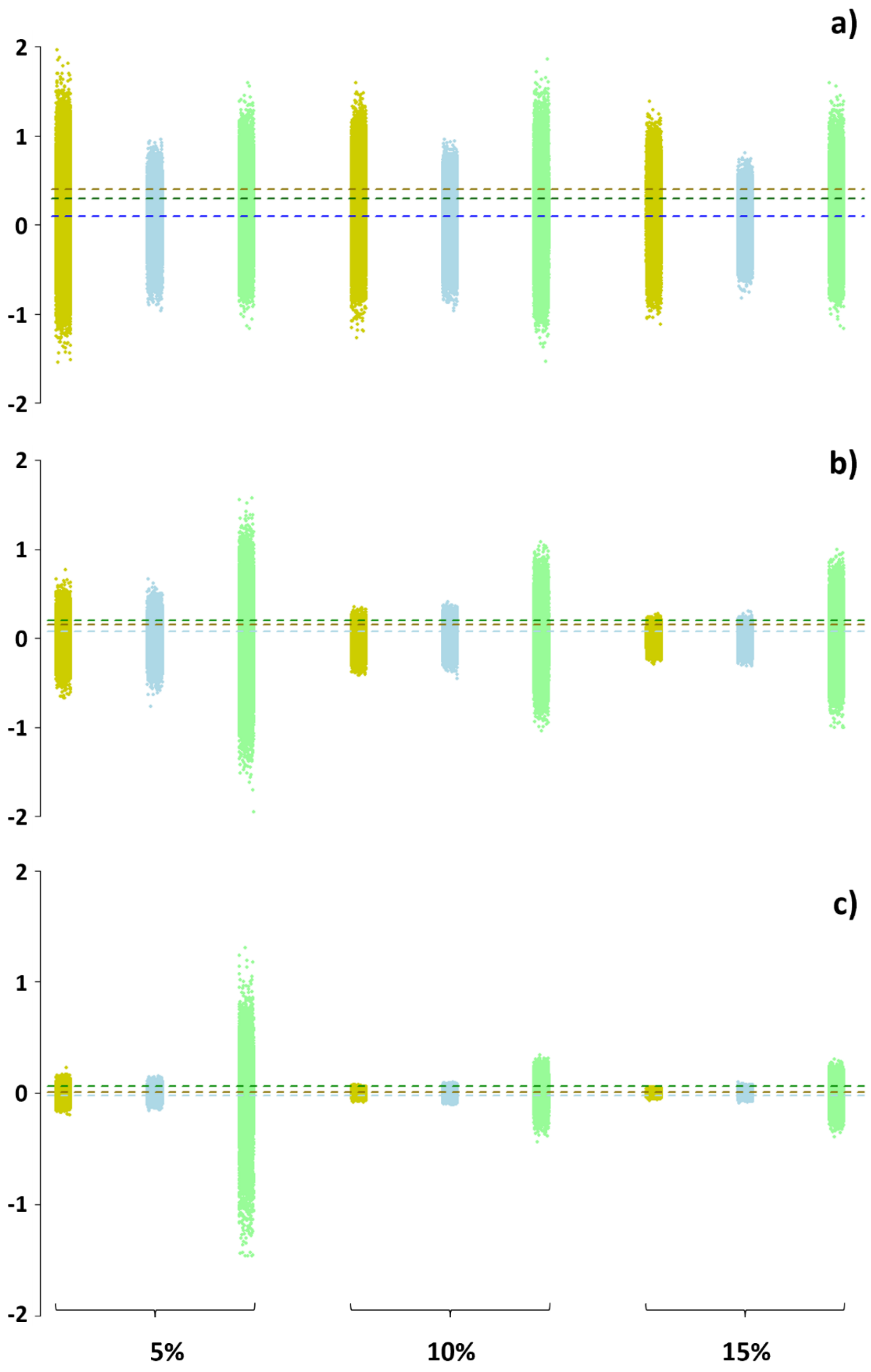

Supplementary Fig. 10: Simulation results – Continuous variables. Posterior distribution (100 generated datasets, 100,000 draws) of the estimated effect of continuous variables varying by region - region 1 (yellow), 2 (blue), 3 (green) - on consumption probability (a) frequency of consumption (b) and quantity consumed (c) for 3 different scenarios of survey coverage. The real simulated values are represented by the yellow (region 1), blue (region 2) and green (region 3) horizontal dashed lines.

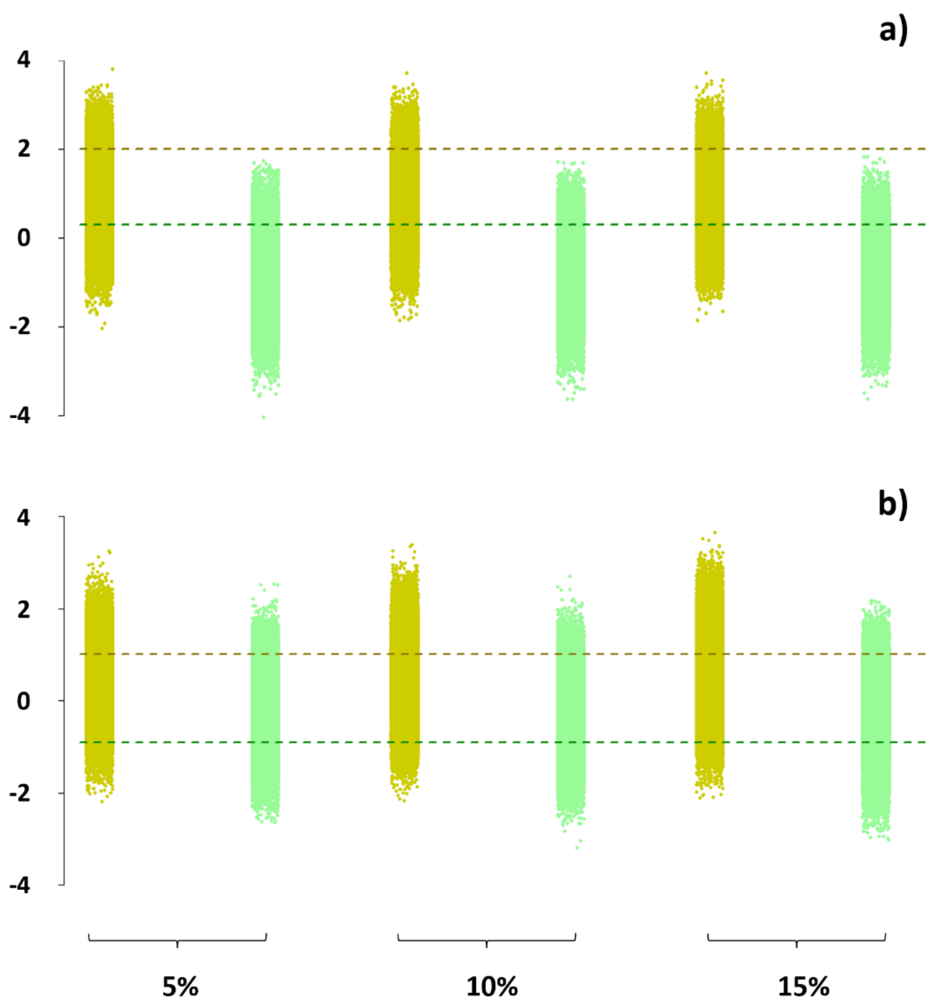

Supplementary Fig. 11: Simulation results – Categorical variables. Posterior distribution (100 generated datasets, 100,000 draws) of the estimated effect of categorical variables - level 1 (yellow) and 2 (green) - on consumption probability (a) and frequency of consumption (b) for 3 different scenarios of survey coverage. The real simulated values are represented by the yellow (level 1) and green (level 2) horizontal dashed lines.

Variables selection

Consumption probability

When we evaluated the full sub-model, we observed severe issues of collinearity between human development index *HDI* and remoteness *REM* (Supplementary Fig. 11a). However, the addition of a spatial component solved most issues (Supplementary Fig. 11b) and significantly improved the model's predictive power (Difference in ELPD = 50.3, Standard Error = 9.9). Although some minor problems remained, for the final analysis we decided to consider all continuous variables and included spatial autocorrelation to limit issues of collinearity. Nevertheless, we further checked for collinearity issues after running the model including the full dataset (see "Model diagnostics" below).

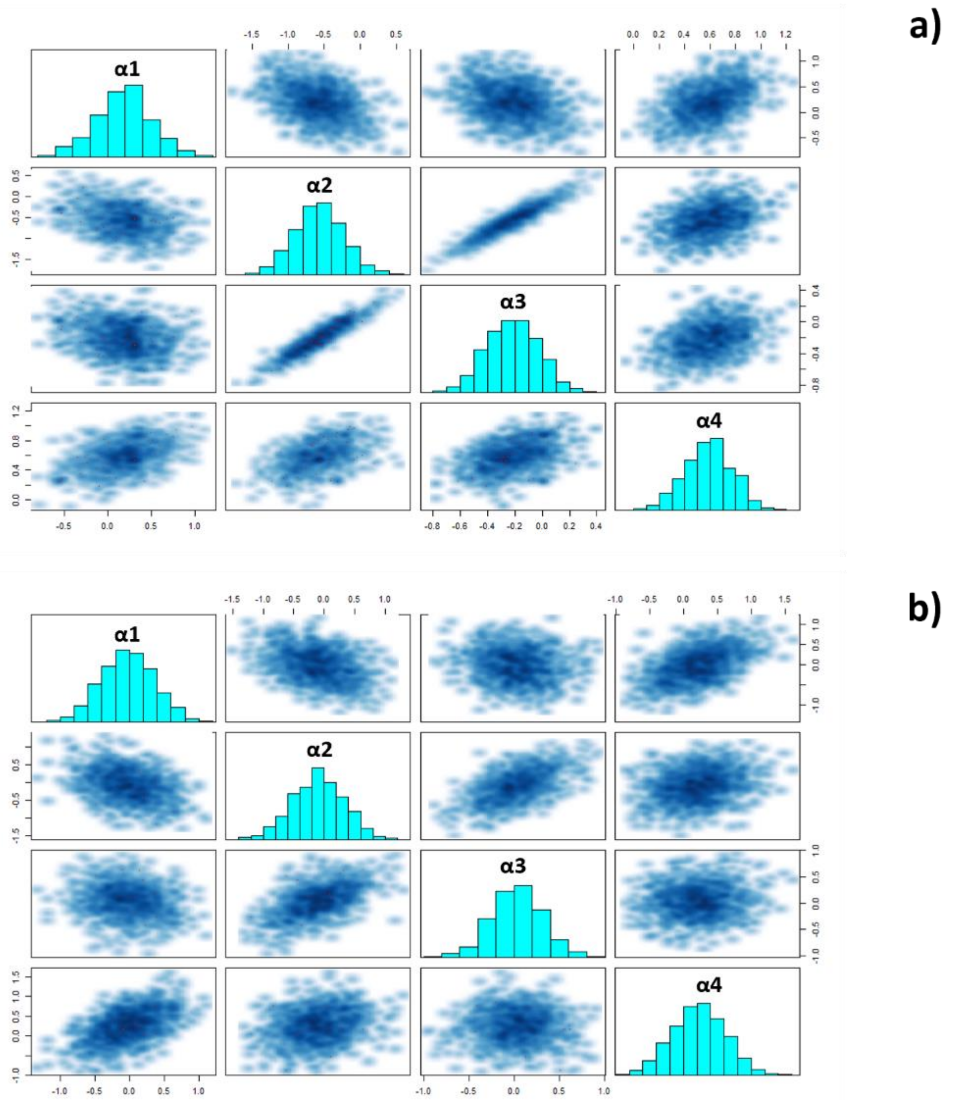

Supplementary Fig. 12: Pair plots (n = 2,000 posterior draws) used to evaluate collinearity in the full model of consumption probability. Considered continuous variables: Human Population Density (α1); Remoteness (α2); Human Development Index (α3); Forest Condition Index (α4). Top: does not include spatial autocorrelation. Bottom: includes spatial autocorrelation.

As we were also interested in testing for different responses to human population density *HPD* between rural villages, towns, and cities (i.e., location type *LT*), we included an interaction on α1 and replaced Eq. 3 (see Methods "Model definition"), with the equation in Extended Data Fig. 1a.

Frequency of consumption

As before, the full model without spatial autocorrelation component showed issues of collinearity between *REM* and *HDI* (Supplementary Fig. 12a). However, as including a spatial component mostly solved the problem (Supplementary Fig. 12b) and improved predictive power (Difference in ELPD = 6.0, SE = 2.7), we selected the model including all four continuous variables as well as the spatial autocorrelation component. We evaluated the interaction between *LT* and *HPD*, replacing Eq. 7 (see Methods - Model definition), with the equation in Extended Data Fig. 1b.

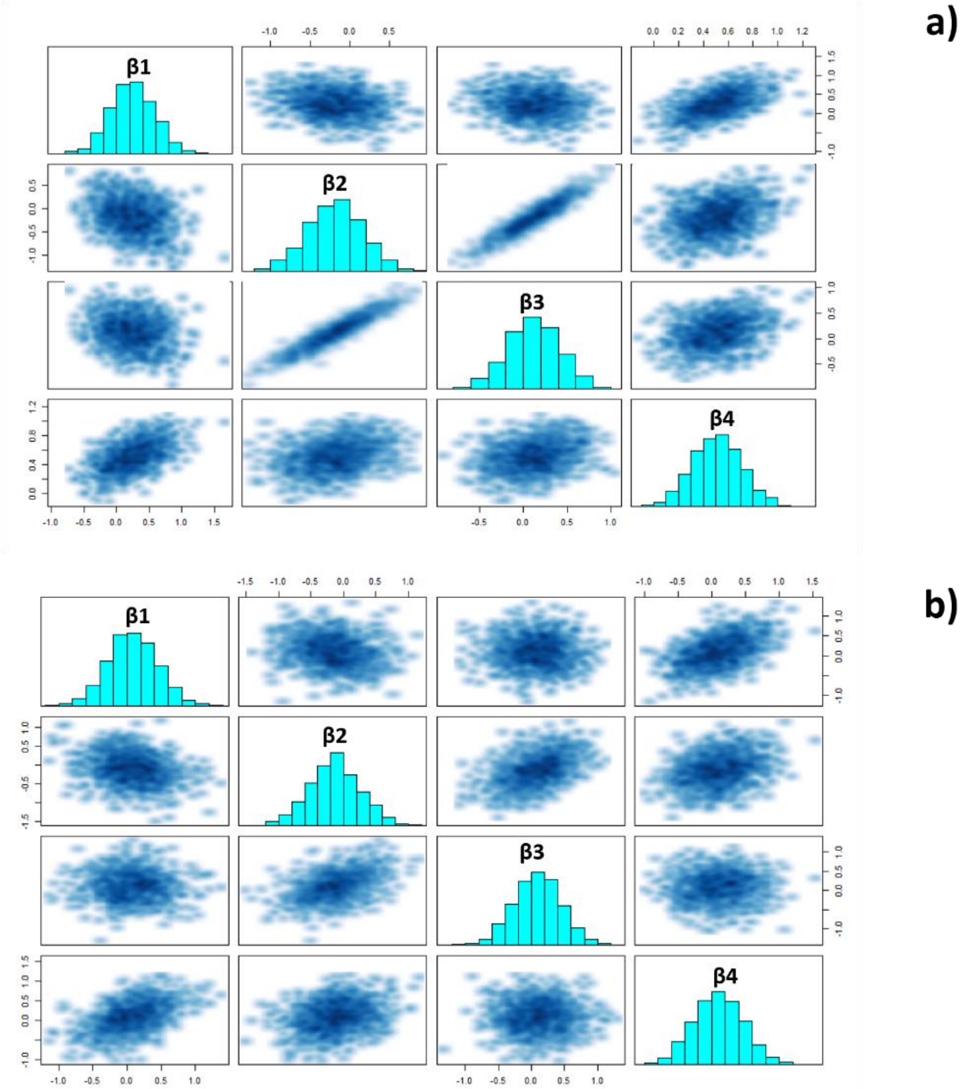

Supplementary Fig. 13: Pair plots (n = 2,000 posterior draws) used to evaluate collinearity in the full model of frequency of consumption. Considered continuous variables: Human Population Density (β1); Remoteness (β2); Human Development Index (β3); Forest Condition Index (β4). Top: does not include spatial autocorrelation. Bottom: includes spatial autocorrelation.

Quantity consumed

The full model did not show major problems of collinearity (Supplementary Fig. 13) and we did not expect spatial autocorrelation to have an influence on the quantity of wild meat consumed. Therefore, we tested if including continuous variables improved the predictive power of the model. We found that none of them did, and that a “null” model, with no continuous variables, had a difference in ELPD of 4 and a standard error= 3.7, only 0.3 lower than the estimated difference in ELPD. As these results strongly suggested negligible differences in predictive power between the “null” and the “full” model (Difference in ELPD = 4.0, SD = 3.7), for the final analysis we selected the simplest model, only including categorical and random variables, plus the number of AME present at the recall, and replaced Eq. 10, with the equation in Extended Data Fig. 1c.

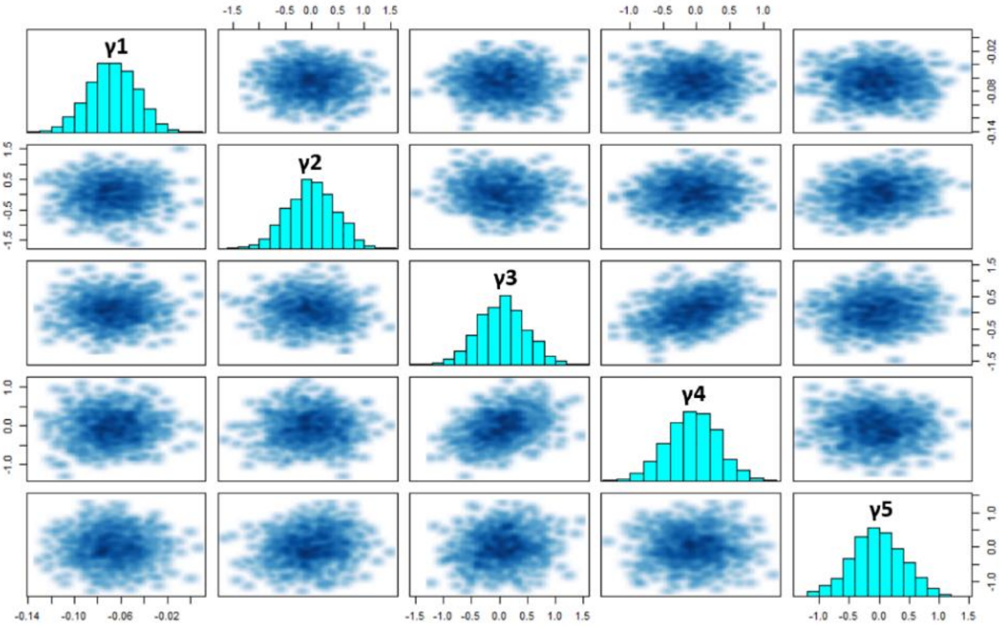

Supplementary Fig. 14: Pair plots (n = 2,000 posterior draws) used to evaluate collinearity in the full model of quantity consumed. Considered continuous variables: number of Adult Male Equivalent units (γ1); Human Population Density (γ2); Remoteness (γ3); Human Development Index (γ4); Forest Condition Index (γ5). The plot shows no major issues of collinearity.

Model diagnostics

Our model converged well, with Rhat values of all parameters below 1.05 (Extended Data Table 2) and satisfactory mixing of the four chains (Supplementary Fig. 15). The final model also did not show any issue of collinearity (Supplementary Fig. 16), significantly improving our preliminary tests (see "Variable selection" above).

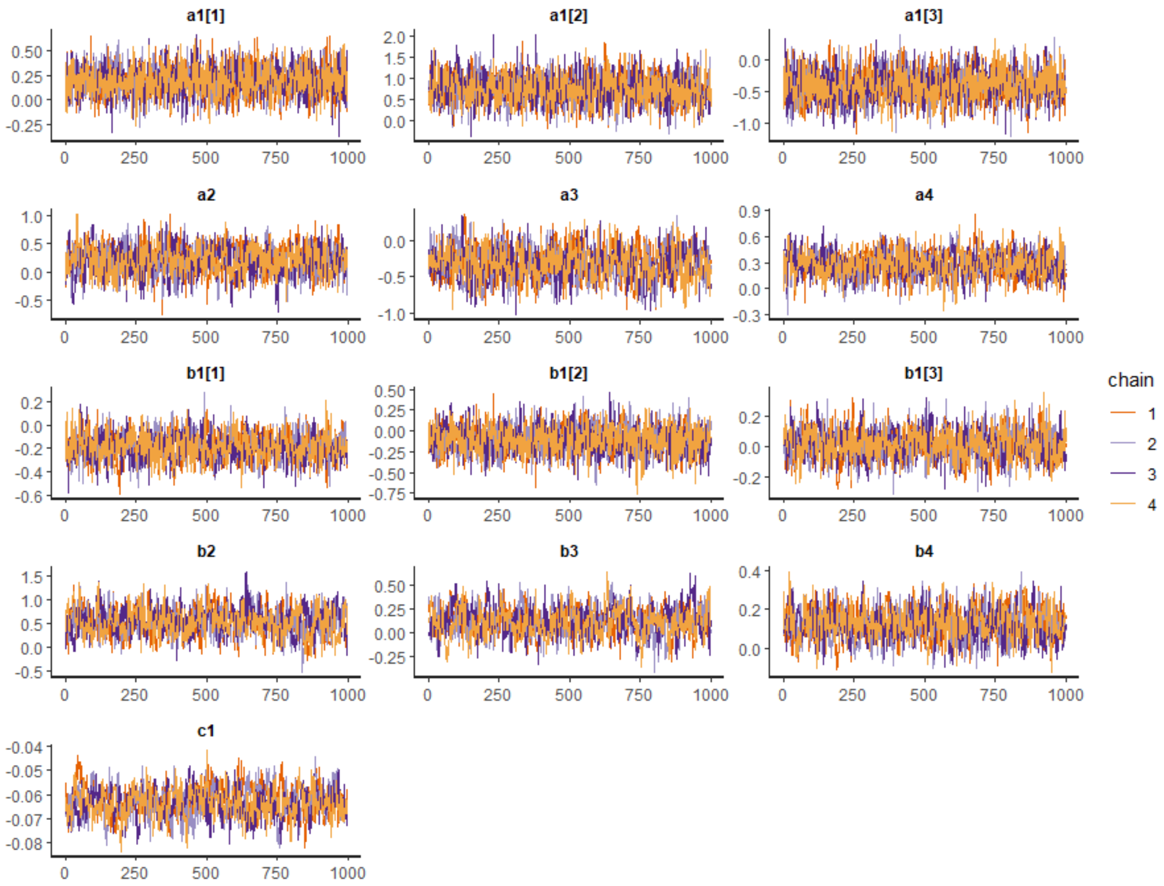

Supplementary Fig. 15: Trace plots used to evaluate the model convergence (4 chains, 1,000 iterations each). Only parameters of the effect of continuous variables shown to allow readability of the plot. Consumption probability: *HPD* (α1); *REM* (α2); *HDI* (α3); *FCI* (α4); Frequency of consumption: *HPD* (β1); *REM* (β2); *HDI* (β3); *FCI* (β4); Quantity consumed: AME (γ1).

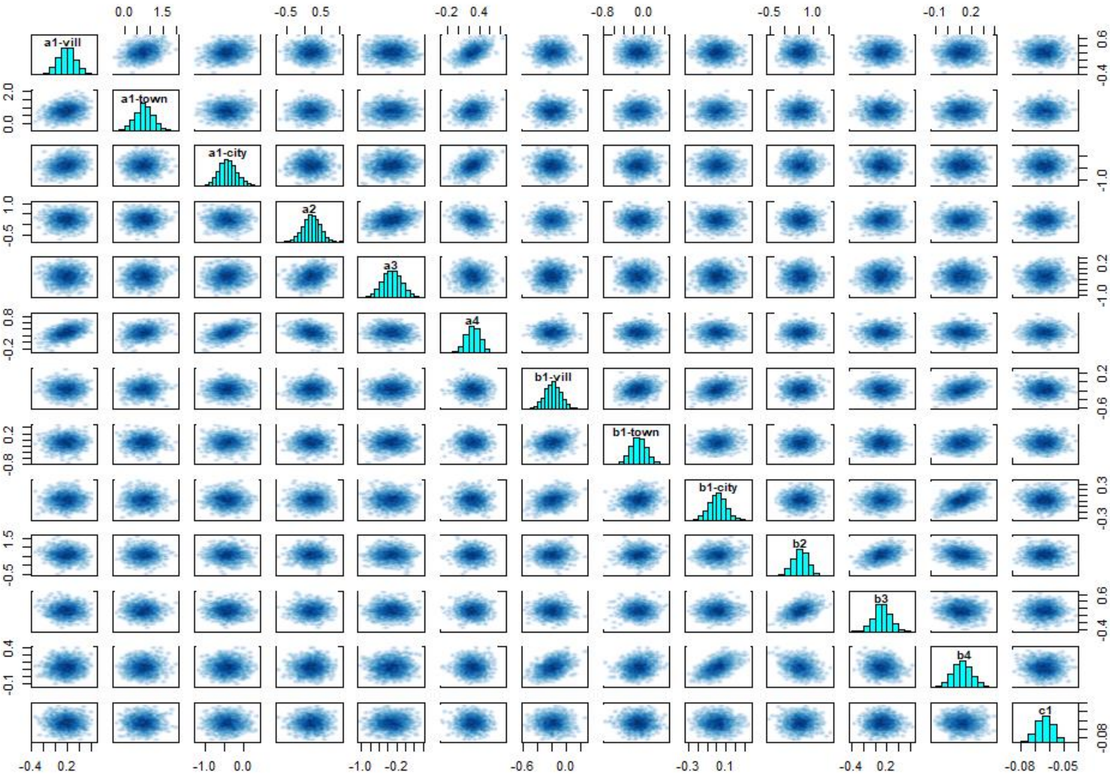

Supplementary Fig. 16: Pair plots used to evaluate collinearity in the final model (4,000 posterior draws). Only continuous variables are shown to allow readability of the plot. Consumption probability: *HPD* (α1); *REM* (α2); *HDI* (α3); *FCI* (α4); Frequency of consumption: *HPD* (β1); *REM* (β2); *HDI* (β3); *FCI* (β4); Quantity consumed: AME (γ1).

Supplementary discussion

Database limitations and possible improvements

Estimated weights

In our database, 9 out of 19 studies providing data on the quantity of wild meat consumed did not always record the exact weight (in grams of kilograms) consumed in the household during the recall period. In some cases, they only noted consumed local units, whereas in other they weighed the meat consumed in certain occasions but did not in other (Extended Data Table 1). A full list of the units included in the database, with their definition is provided below (Supplementary Table 7).

Supplementary Table 7: List of wild meat local units found in our database.

Unit	Definition	Language
Entire	Entire animal	English
Gigot	Leg and shoulder	French
Half	Half carcass	English
Head	Head of the animal	English
Intestines	The intestines of the animals	English
Kilogram	Weighed or estimated weight	English
Morceau	A piece of meat, can be of different sizes depending on the area	French
Packet	A small pile of meat, usually composed of ~10 small pieces, but sold wrapped in leaves or plastic bags	English
Plate	A plate, usually consumed in restaurants	English
Quarter	The hind or rear quarter of a carcass	English
Ribs and leg	Like a gigot, but includes the ribs	English
Tas	A small pile of meat, usually composed of ~10 small pieces	French
Tail	The tail of the animal	English
Viscera	Includes intestines, liver, hearth, kidneys, and lungs of the animal	English

For this reason, we estimated consumed kilograms following procedures specific for each unit. If the consumed units were reported as “entire”, “half”, “quarter” or “gigot” we assigned the species-specific average mass value using data available from the literature 59 or empirical data obtained in Gabon by authors of this study (LC, Dibouka, 2001 -2010; DF & DC, Lastourville area, 2021 [123]). For all other units (3) including “piece”, “pile”, “plate”, etc. we used estimated conversion factors, based on empirical observation collected by various authors of this study (LC, KA, FS, DD). In case we were unable to estimate a reasonable weight, we assigned a missing value and treated the data-point as if we had no information on quantity consumed. Although often based on empirical data and field observations, estimated values approximated the real quantity consumed and might not be entirely accurate.

Coverage and representativity

Although the number of sites and households included in our database is unprecedented in wild meat literature, it only included data regarding a minimal fraction of the entire population of Central Africa (~60,000 people or 0.0005% of the population in 2022). Similarly, the geographical coverage of our sites is, although impressive, incomplete (Main text, Fig. 1). Large swaths of the Democratic Republic of the Congo and Central Africa Republic are not represented, and so are the North and Centre of Cameroon. In addition, the studies included in our database concentrated around forested areas, with only two having been conducted in the forest-savannah mosaic. As such, the estimated consumption rates and resulting biomass consumed shown in Fig. 3, are less accurate in savannahs and semi-desertic areas, the North (Central Africa Republic and Cameroon) of the Central African region, as well as in areas of high population density. In addition, because most studies conducted in urban areas focussed on individual, rather than household consumption, our database only include data collected in four cities and 24 towns. Although this was not ideal, the limited representativeness of cities and towns in the database translates in the uncertainty of the estimated parameters (Extended Data Table 3), not impacting our estimates. Below, we expand the discussion of these results, considering current knowledge of wild meat consumption in Central Africa and the associated uncertainty maps (Extended Data Fig. 4).

Remote classification of settlement type

For our predictions we needed a standardized categorisation of settlement type based on available data across the entire region to ensure replicability. Unfortunately, there is no regional, nor national, database available in Central African countries providing a classification for each settlement. In the same way, there are no databases of, e.g. the facilities present in each settlement, which could be used to attempt a facility-based classification. As done by several other studies, either focussing specifically on wild meat [30,90,91] or more generally on urbanization [92-95], the only tested and replicable approach to (remotely) classify villages, cities and towns across Central Africa is to use population size. Based on the classification made by data providers, this approach allowed us to correctly classify 229 of the 252 locations in our database (91%). As all cities were corrected classified, the remaining 9% were towns (n = 12) and villages (n = 11). Towns identified as villages were all surrounded by sparse buildings in an otherwise rural landscape. Therefore, only their centre was classified as urban by the global settlement layer [6]. As a result, these towns were identified as having < 10,000 inhabitants and were assigned to the category "village" (e.g., Fougamou, GAB). In contrast, villages identified as towns, were all located in the highly populated Eastern DRC and fell within areas categorised as urban by the global settlement layer [33].

While the cut point of 10,000 inhabitants has been used previously to discern villages and towns [92], we could also have selected a different cut point to identify towns, for example 5,000

inhabitants [90]. However, we suspect that this approach would have resulted in a higher difference in consumption rates between villages and towns, giving further support to our results. Our prediction is confirmed by the model including only two settlement type: rural and urban (Extended Data Fig. 5). By aggregating towns and cities, the estimated consumption rates for major cities like Kinshasa and Yaoundé were not too different from those estimated for rural settlements (Extended Data Fig. 5). Accordingly, the biomass consumed in these cities increased massively (Extended Data Fig. 5d). Lowering the urban cut-point to, e.g., 5,000 people, would results in even higher urban consumption rates, as city-dwellers will be assumed to consume wild meat in similar quantities as people living in small rural towns.

Instead, we took a more conservative approach, by including settlement type as a random slope, defining the interaction between the type of settlement and population density (Extended Data Fig. 1a, Extended Data Fig. 1b, Eq. 22, Eq. 23.), an approach that "...largely eliminates the risk of strong false positives but reduces the chance of obtaining strong evidence for true effects" [124]. Finally, in the only case where we explicitly included settlement type in the model (quantity - Extended Data Fig. 1c, Eq. 24), the estimated parameters for "village", "town" and "city" did not differ significantly (Extended Data Table 3), indicating a limited impact on the final estimates.

Single-year predictors

When compiling site-specific continuous variables potentially affecting wild meat consumption in Central Africa (human population density "HPD", remoteness "REM", human development index "HDI" and forest condition index "FCI"), we aimed to use layers that filled the following criteria: 1) coverage of the entire central African region; 2) resolution =< 1km^2; 3) multi-year data covering the entire period of study (i.e., 2001-2021). However, multi-year layers of remoteness and forest condition were not available, and so were layers providing region-wide, fine scale HDI data. As a result, we selected the layers that most closely matched our criteria. While we do not expect our predictions to be affected by the use of low-resolution (i.e., administrative level 1) HDI data, we acknowledge that using single year layers might have reduced the power of our model in correctly detecting temporal trends in wild meat consumption. In fact, in several part of the region both REM and FCI were likely higher in 2001-2010, when me lack REM and FCI data. If this was true, we might have underestimated wild meat consumption rates for the period 2000-2010. In fact, as 1) remoteness has a positive on wild meat frequency of consumption (Fig. 2) and 2) FCI has a positive effect on both wild meat probability and frequency of consumption (Fig. 2) consumption rates would have been predicted to be higher in all areas with values of REM and FCI in 2001-2010 higher than in 2015 (REM) and 2019 (FCI). Accordingly, the underestimation of "past" consumption rates would result in a decrease in the difference in the total biomass consumed between periods. All other results would be mostly unaffected.

Geographical variation in consumption rates (Fig. 3a and 3b)

We found the highest rates of wild meat consumption, hence the highest contribution of wild meat to the recommended daily protein intake, in northern Republic of Congo, at the border with Southeast Cameroon and the Sangha-Mbaéré province in Central Africa Republic. This area is one of the remotest of the region and, being home to Noubalé Ndoki (ROC) and Dzanga-Sangha (CAR) National Parks, one of the strongholds of wildlife in the continent. Wild meat consumption is reportedly high in this specific region [107], and estimated rates are in line with the data from the same area included in the analysis (three studies), confirming the prediction of our model. Another area where we predicted similar consumption rates includes the Haut-Mboumou and Mboumou Provinces, in East Central Africa Republic. Although we did not have data to support our findings, reports from the nearby Chinko Natural Reserve suggest that wild meat consumption is a significant threat to wildlife and includes commercial hunting perpetrated by livestock herders, seasonally entering the area from Sudan, and military militia [125-127]. The plausibility of our results is further supported by evidence for the importance of wild meat in savannah regions [128], and by considering that the area is very remote (Supplementary Fig. 2), has one of the lowest HDI of the continent (Supplementary Fig. 4), and has recently suffered civil unrest and the presence of armed militia, all factors known to favour wild meat consumption [24]. However, we wish to stress that the precision of our estimates in the area is low (Extended Data Fig. 4a and b) and estimated rates should be considered with care. Our model also predicts high consumption rates along the Congo River in Democratic Republic of the Congo, both on the right and left banks, and in northeast Gabon, at the border with the Republic of Congo. Both areas are characterized by the proximity to healthy forests, including the parks of Salonga, Lomami and Kahuzi-Biega National Parks (DRC), Odzala-Kokoua (ROC) and Minkebe, Ivindo and Mwagna (GAB).

Conversely, we predicted the lowest consumption rates in urbanized areas, like regions of Douala and Yaoundé (CMR), Brazzaville and Pointe Noire (ROC), Kinshasa, and the Kasai and Kivu Provinces (DRC). All these areas have easy access to alternative food items and wild meat is expected to be expensive. However, when we assessed consumption rates by aggregating towns and cities (Extended Data Fig. 5; Supplementary Table 6), we obtained higher median consumption rates both for the Central African forest region (57 g/AME/day; mean = 123; SD = 218), and the entire Central Africa (median = 56 g/AME/day; mean = 121; SD = 214). This increase is likely due to the higher consumption rates estimated in major cities because assuming similar consumption rates in small towns and large cities of more than 100,000 inhabitants. Remarkably, the model also predicts very low consumption rates in North Cameroon (with good precision – Extended Data Fig. 3b), a semi-arid region where wildlife had been severely depleted and livestock rearing is well developed [129].

Nevertheless, we do not have data to support our findings (Extended Data Fig. 3a), nor we are aware of recent studies that investigated wild meat consumption in the area.

Geographical variation in biomass consumed (Fig.3d)

The geographic variation in biomass consumed shows a pattern opposite to what described for consumption rates. Here, hotspot of biomass consumed are found around areas of high population density. Our results point out that perhaps the largest quantity of biomass is consumed in highly populated areas far away from capital cities. The Kasai and Kivu regions (DRC) are the most striking. Here, consumption rates are generally higher than those estimated for capital cities, due to the proximity to areas of high forest condition, where wild meat is sourced and has been characterized by high rates of population growth and urbanisation [33,77] since the end of the Second Congo war (1998-2003). Interestingly, most capital cities, including Yaoundé (CMR), Kinshasa (DRC), Brazzaville (ROC) and Libreville (GAB), do not stand out as hotspots. The only capital city showing up as a major hotspot is Bangui (CAR), perhaps the only capital city where wild meat was reported being cheaper than alternatives sources of protein [130]. Here too, we wish to highlight that although we are confident about the relative importance of these areas on the regional biomass consumed, the low precision of our estimates (Extended Data Fig. 3a and c) advises to consider with care the absolute values estimated by the model and provided in the maps. For example, the Kinshasa/Brazzaville area, with a population approaching 20,000,000 people is an extreme outlier in the region. Here, because consumed biomass is the product of estimated consumption rate and the population living in the area, even a minimum increase in consumption rates would imply a major increase in the biomass consumed per year. This is well exemplified by our analysis including only two settlement types, rural and urban (Extended Data Fig. 5; Supplementary Table 6). By aggregating cities and towns, the consumption rate in cities increased considerably (Extended Data Fig. 5) due to the flattening effect of considering major cities > 100,000 people like small towns of 10,000-15,000 inhabitants. Accordingly, the large number of people living in major cities like Kinshasa and Yaoundé, resulted in an increase in the biomass consumed in cities, which are then highlighted as the main hotspots of wild meat consumption in the region (Extended Data Fig. 5) and resulted in a 25% increase in the median wild meat biomass estimated to be consumed in Central Africa in 2022: from 1.62 million tonnes (mean = 2.37; SD = 2.42; 95% CI = 0.27 – 8.77) to 2.03 million tonnes (mean = 3.13; SD = 3.68; 95% CI = 0.33 – 12.04). As it is well known that people of these cities have low consumption rates (a few times/year), which is not the case for smaller towns, the three-settlement approach highlighted the differences in consumption rates between cities and towns, allowing for more realistic results.

Conversely, despite showing highest consumption rates (see above) the contribution of the remotest areas of the region on total biomass consumed appear negligible. Remarkably, these are also the areas where the reliance on wild meat for nutritional need is likely to be highest (Fig. 3c), like the interior of the Democratic Republic of the Congo, the North of the Republic of Congo and large swaths of Central Africa Republic. In these cases, the precision of predicted values was good (Extended Data Fig. 4c) although the majority of Eastern Central Africa Republic showed features highly dissimilar from the sites included in our database (Extended Data Fig. 4a).

Temporal changes in biomass consumed (Extended Data Fig. 5)

Some of the areas of highest consumed biomass described above area also those where our model predicted the largest increase in biomass consumed between periods (2000-2010 vs 2011-2021), most of them in Democratic Republic of the Congo (DRC). The main hotspot is found in the Kasai Region (DRC), including the cities of Mbuji Mayi, Kananga and Tshikapa, an area characterised by high rates of population growth and urbanisation, currently attracting thousands of people from other areas of the country. Although the area enjoys relatively good connection with the capital city, Kinshasa, imported good are generally delivered via river, a dangerous trip that takes several days. As a result, wild meat sourced from forested regions to the north is expected to be still the cheapest source of meat, favoured by new inhabitant coming from rural areas. A similar pattern might explain the high increase in biomass consumed predicted in Goma and Lubumbashi, two cities of > 1,000,000 inhabitants where wild meat is consumed by a large proportion of the population (86.6% in Lubumbashi [131]). Another hotspot is represented by the savannah areas surrounding Upemba National Park (DRC), where estimated consumption rates were relatively high (in addition to Upemba, Kundelungu National Park also is accessible from the area). Although today this area is still mostly rural and characterized by the presence of small towns, rather than large cities, human population have been growing significantly over the past 20 years [33,77]. Apart from the Democratic Republic of the Congo, areas of increased consumed biomass are all areas of increasing urbanisation, like the Franceville/Moanda area in Gabon and the Northwest region of Cameroon.

Interestingly one of the few areas where biomass consumed appear to be decreasing is also in DRC, around the cities of Boma and Matadi. The latter is the main seaport of the country, where most imported goods are delivered. Cheap imported meat, in combination with large availability of fish due to the proximity to the Atlantic Ocean and Congo River and depletion of wildlife populations might have favoured a shift towards other sources of protein within the population of the area.

Supplementary references

122 Jung, M. *et al.* A global map of terrestrial habitat types. *Scientific Data* **7**, 256 (2020). https://doi.org:10.1038/s41597-020-00599-8

123 Cornélis, D. *et al.* "Hunting offtake dataset of the project "Sustainable Wildlife Management" in Gabon (Mulundu Department).", https://doi.org/10.18167/DVN1/75ZDAM, CIRAD Dataverse, V1 (2023).

124 Oberauer, K. (2022). The Importance of Random Slopes in Mixed Models for Bayesian Hypothesis Testing. *Psychological Science*, *33*(4), 648-665. https://doi.org/10.1177/09567976211046884

125 Aebischer, T. *et al.* Apex predators decline after an influx of pastoralists in former Central African Republic hunting zones. *Biological Conservation* **241**, 108326 (2020).

126 Ondoua Ondoua, G. *et al.* An assessment of poaching and wildlife trafficking in the Garamba-Bili-Chinko transboundary landscape. Report No. 1858504260, (Traffic, 2017).

127 Blom, A., Yamindou, J. & Prins, H. H. Status of the protected areas of the Central African Republic. *Biological Conservation* **118**, 479-487 (2004).

128 Van Velden, J. L. *et al.* Bushmeat hunting and consumption is a pervasive issue in African savannahs: insights from four protected areas in Malawi. *Biodiversity and Conservation* **29**, 1443-1464 (2020).

129 Scholte, P. Population trends of antelopes in Waza National Park (Cameroon) show escalating effects of poaching and livestock intrusion. *African Journal of Ecology* **52**, 370-374 (2014).

130 Fargeot, C., Drouet-Hoguet, N. & Le Bel, S. The role of bushmeat in urban household consumption: Insights from Bangui, the capital city of the Central African Republic. *Bois & Forets Des Tropiques* **332**, 31-42 (2017).

131 Tshikung, K., Pongombo, S., Roland, L. & Hornick, J. Consumption of Bushmeat in Lubumbashi/DR Congo: Sociocultural Approaches. *Journal of Health Science* **7**, 79-88 (2019).
