# Supplementary material for: Increase in wild animal consumption across Central Africa
Source: Nature. 2026 Apr 29;653(8116):1092–8. doi: 10.1038/s41586-026-10422-w (PMC13216070; doi:10.1038/s41586-026-10422-w)
Supplement: Supplementary file 2 — Reporting Summary [file 41586_2026_10422_MOESM2_ESM.pdf]

nature portfolio

Corresponding author(s): Mattia Bessone

Last updated by author(s): Feb 10, 2026

Reporting Summary

Nature Portfolio wishes to improve the reproducibility of the work that we publish. This form provides structure for consistency and transparency in reporting. For further information on Nature Portfolio policies, see our Editorial Policies and the Editorial Policy Checklist.

Statistics

For all statistical analyses, confirm that the following items are present in the figure legend, table legend, main text, or Methods section.

n/a	Confirmed	
☐	☒	The exact sample size (*n*) for each experimental group/condition, given as a discrete number and unit of measurement
☐	☒	A statement on whether measurements were taken from distinct samples or whether the same sample was measured repeatedly
☒	☐	The statistical test(s) used AND whether they are one- or two-sided<br>*Only common tests should be described solely by name; describe more complex techniques in the Methods section.*
☐	☒	A description of all covariates tested
☒	☐	A description of any assumptions or corrections, such as tests of normality and adjustment for multiple comparisons
☐	☒	A full description of the statistical parameters including central tendency (e.g. means) or other basic estimates (e.g. regression coefficient) AND variation (e.g. standard deviation) or associated estimates of uncertainty (e.g. confidence intervals)
☒	☐	For null hypothesis testing, the test statistic (e.g. *F*, *t*, *r*) with confidence intervals, effect sizes, degrees of freedom and *P* value noted<br>*Give P values as exact values whenever suitable.*
☐	☒	For Bayesian analysis, information on the choice of priors and Markov chain Monte Carlo settings
☐	☒	For hierarchical and complex designs, identification of the appropriate level for tests and full reporting of outcomes
☒	☐	Estimates of effect sizes (e.g. Cohen's *d*, Pearson's *r*), indicating how they were calculated

Our web collection on statistics for biologists contains articles on many of the points above.

Software and code

Policy information about availability of computer code

Data collection	Most recent studies included in our analysis used KoboToolBox https://www.kobotoolbox.org/, and different versions of the KoboCollect App (ver. 2.020.40 and subsequent releases). The software is open-source.<br>All other studies did not use software for data collection and recorded data with pen and paper.
Data analysis	We prepared the data, ran the models and drew figures in R 4.2.0 77. We coded our models in Stan using the R package "rstan", ver. 2.26.11. For model selection, we used the R package “loo”, ver. 2.5.1 We drew figures using the R package "base", ver. 4.3.1. Maps shown in figures were created using QGis 3.22.1. All softwares are open-source. The Stan and R code used for analyses are provided at: https://github.com/mattiabessone/Wild-animal-consumption-is-increasing-in-Central-Africa

For manuscripts utilizing custom algorithms or software that are central to the research but not yet described in published literature, software must be made available to editors and reviewers. We strongly encourage code deposition in a community repository (e.g. GitHub). See the Nature Portfolio guidelines for submitting code & software for further information.

Data

Policy information about availability of data

All manuscripts must include a data availability statement. This statement should provide the following information, where applicable:

- Accession codes, unique identifiers, or web links for publicly available datasets
- A description of any restrictions on data availability
- For clinical datasets or third party data, please ensure that the statement adheres to our policy

Data availability statement (provided in the manuscript): "Our study uses raw data from studies conducted from the year 2000 until 2022 and thus did not generate new data. Wild meat consumption data were extracted from different published and unpublished sources as described in Extended Data Table 1. Due to the sensitive nature of the data (including illegal activities, such as the consumption of protected wildlife species), unprocessed datasets are available with restrictions through the WILDMEAT Data Portal (https://explorer.wildmeat.org/). Each dataset is available under different data sharing conditions through a Data User Agreement, which gives data users control over the distribution and use of their data. The full processed dataset used for analysis will be shared upon request with researchers seeking to replicate the study results. Other requests must clearly specify the study objectives, and access will be granted on a case-by-case basis following permission from the original data providers. In all cases, data recipients will be required to abide by the data-sharing agreements of WILDMEAT. All requests should be addressed to: mattia.bessone@gmail.com. The data needed to reproduce the figures and maps shown in the main text are available at https://doi.org/10.5281/zenodo.19021125. The spatial layers used in our analysis are described in Extended Data Table 2 and are available at: https://www.forestintegrity.com/ (forest condition index – under CC BY 4.0), https://human-settlement.emergency.copernicus.eu/ghs_pop2019.php/ (human population density – under CC BY 4.0), https://human-settlement.emergency.copernicus.eu/ghs_smod2023.php (settlement type – under CC BY 4.0), https://malariaatlas.org (remoteness – under CC BY 3.0), https://globaldatalab.org/ (Sub-national Human Development Index), https://dhsprogram.com and https://mics.unicef.org (education level). Forest blocks shown in Fig. 1 are available at https://data.mendeley.com/datasets/7gskp92yx6/1 under a CC BY 4.0."

Research involving human participants, their data, or biological material

Policy information about studies with human participants or human data. See also policy information about sex, gender (identity/presentation), and sexual orientation and race, ethnicity and racism.

Reporting on sex and gender	Our study is a meta-analysis that uses the raw data from many other studies. Although information about the sex and gender of responders was collected in some studies, we did not use this in our meta-analyses.
Reporting on race, ethnicity, or other socially relevant groupings	We use two socially relevant categorization variable in our study. The "human development index" and the "education level" of the respondents. The human development index HDI is a freely available subnational indicator of human development, calculated as the geometric mean of the normalized indices of 1) life expectancy at birth, 2) average years (for adults >25 years) and expected years of schooling for children; 3) gross national income per capita. The values used in our study are a translation of the UNDP's official HDI and GDI (hdr.undp.org) to the subnational level, i.e. the first administrative level of each country considered in our study. The methods used to calculate the index are described in Smits, J., Permanyer, I. The Subnational Human Development Database. Sci Data 6, 190038 (2019).<br>The "education level" of each respondent was provided by the respondent (i.e., self-report) in interviews. For this study we only considered two categories: 1) highest education was primary (or no education); 2) highest education was secondary (or higher). In this case, we also controlled for the confounding effect of settlement type (village, town or city), which can affect the quality of education.
Population characteristics	Although in some cases, information about the age and sex of individual respondents were available, we did not use this information in our analyses as we restricted our research to studies investigating wild meat consumption at the household level, discarding those monitoring consumption of individual consumers. Accordingly, the data used in our study referred to the entire households and were thus not biased by, e.g. sex and age of the respondents.
Recruitment	In our meta-analysis we considered peer-reviewed articles, technical reports, PhD and Master's dissertations, online data repositories and unpublished data, adopting a snowball sampling approach to search reference lists and online libraries. We used "wildmeat"; "wild meat"; "bushmeat"; "bush meat"; "viande de brousse" as main keywords, and "consumption"; "nutrition"; "food" as secondary keywords. We defined a "study" as a set of data collected using a single methodology in a specific study area over a determined timeframe. In this way, each data source could provide more than one study. For example, large projects that monitored multiple regions in different countries, were split so that each study area represented a single study. For consistency, we restricted our research to studies investigating wild meat consumption at the household level, discarding those monitoring consumption of individual consumers who could not be aggregated to households, for example by enquiring people randomly met in the streets, a methodology mostly used in cities, where household surveys are difficult to implement. When possible, we downloaded the raw data from online resources (e.g., publicly available databases). Alternatively, we contacted the authors to request the raw data.
Ethics oversight	Ethics review committee of CIFOR/ICRAF (project n: SLF6430000-UFW044-AI2; 13/12/2021)

Note that full information on the approval of the study protocol must also be provided in the manuscript.

Field-specific reporting

Please select the one below that is the best fit for your research. If you are not sure, read the appropriate sections before making your selection.

☐ Life sciences ☒ Behavioural & social sciences ☐ Ecological, evolutionary & environmental sciences

For a reference copy of the document with all sections, see nature.com/documents/nr-reporting-summary-flat.pdf

Behavioural & social sciences study design

All studies must disclose on these points even when the disclosure is negative.

Study description	Our study is a meta-analysis where we used the raw data from many other studies. While most of these studies provided qualitative data (i.e. the quantity of wild meat consumed per day in a household), some other provided only qualitative data (frequency of consumption, described in qualitative categories). The process used to convert qualitative categories into quantitative frequency data is described in the methods of our manuscript, including how we handled the uncertainty around the reported categories.
Research sample	For our meta-analysis, we aimed to include data from all studies on wild meat consumption conducted in Central Africa at the time of analysis. Following the data recruitment rationale described in the "Recruitment" section above, we gathered data on wild meat consumption originated from 30 studies, representing 12,000 households from 252 locations in Central Africa, including rural and urban sites from the year 2001 until 2022. A detailed description of all datasets included and their source are described in Extended Data Table 1.<br>Although this is the largest dataset of wild meat consumption assembled until today (n = 163,896 data-points), it represents an incomplete sample of the total population (~60,000 people or 0.0005% of the population in 2022) and geographical coverage of Central Africa. To ensure that our sample was representative, we conducted a simulation study, which confirmed that our model's estimates were unbiased even when simulating similar sample size and geographical coverage.<br>In some cases, information about the age and sex of individual respondents were available. However, we did not use this information in our analyses as we restricted our research to studies investigating wild meat consumption at the household level, discarding those monitoring consumption of individual consumers. Accordingly, the data used in our study referred to the entire households and were thus not biased by, e.g. sex and age of the respondents.
Sampling strategy	Our study is a meta-analysis that uses the raw data from many other studies. However, we gathered information about the recruitment of respondents in each study. Most studies used either a fully random [n = 16] or a stratified random (by ethnicity [n = 3], or income [n = 1]) recruitment. In a few cases authors of included studies, used convenience sampling to select collaborating households [n=3]. In the remaining cases [n = 5], we do not have information about household recruitment. In all cases, only household that accepted to collaborate were included.<br>Here we outline that convenience sampling could have resulted in a sample not fully representative of the site being surveyed. However, given that wild meat consumption is highly common in these areas, we expect this bias to be negligible.<br>All studies collected quantitative data on wild meat consumption and recorded only household-related ancillary qualitative data. Accordingly, data saturation was not considered.
Data collection	Our meta-analysis include studies collected from the year 2000 until 2022. Accordingly we were aware of the experimental conditions and conclusions of individual studies when performing the analyses. The methods used for data-collection thus varies by study with older studies using pen and paper, while more recent studies using KoboToolBox https://www.kobotoolbox.org/, and different versions the KoboCollect App (ver. 2.020.40 and subsequent releases). Field researcher were unblinded to experimental conditions but generally blinded to the study hypotheses.
Timing	Data collection starting and ending date of each study (listed in Extended Data Table 1) included in our analysis is reported below:<br><br>ID country start end<br>01 CAF 01/07/2006 25/01/2007<br>02 CMR 09/10/2005 22/11/2006<br>03 CMR 29/03/2009 14/04/2009<br>04 CMR 01/02/2016 28/02/2016<br>05 CMR 13/03/2018 07/06/2018<br>06 CMR 01/04/2019 30/06/2019<br>07 CMR 27/02/2021 18/03/2021<br>08 COD 17/11/2007 02/09/2008<br>09 COD 08/01/2009 09/12/2009<br>10 COD 01/05/2015 31/08/2015<br>11 COD 14/09/2017 11/10/2017<br>12 COD 16/05/2019 31/01/2020<br>13 COD 20/10/2019 08/09/2020<br>14 COG 01/01/2000 31/12/2008<br>15 COG 14/03/2014 16/10/2014<br>16 COG 17/03/2020 19/10/2020<br>17 GAB 03/09/2000 29/11/2002<br>18 GAB 14/06/2001 04/12/2002<br>19 GAB 01/01/2002 28/02/2003<br>20 GAB 01/02/2005 30/12/2005<br>21 GAB 01/02/2006 31/05/2006<br>22 GAB 01/08/2009 31/07/2010<br>23 GAB 19/05/2019 22/09/2019<br>24 GAB 13/02/2021 27/03/2021<br>25 GNQ 01/06/2002 30/06/2002<br>26 GNQ 08/04/2005 28/02/2006<br>27 GNQ 03/07/2009 11/11/2009<br>28 GNQ 01/03/2011 31/03/2012<br>29 NGA 07/12/2007 10/11/2008<br>30 NGA 01/04/2021 31/03/2022

Data exclusions	No data was excluded from the analyses.
Non-participation	Our study is a meta-analysis of 30 different published and unpublished studies, including some conducted in the early years 2000. Unfortunately in most cases we do not have information about drop-off or refusal to participate.
Randomization	For our meta-analysis, participants were not allocated in experimental groups. Four individual studies however, used a stratified random design to ensure equal representation of household from different ethnic groups [n=3] or wealth [n=1].

Reporting for specific materials, systems and methods

We require information from authors about some types of materials, experimental systems and methods used in many studies. Here, indicate whether each material, system or method listed is relevant to your study. If you are not sure if a list item applies to your research, read the appropriate section before selecting a response.

Materials & experimental systems

n/a	Involved in the study
☒	☐ Antibodies
☒	☐ Eukaryotic cell lines
☒	☐ Palaeontology and archaeology
☒	☐ Animals and other organisms
☒	☐ Clinical data
☒	☐ Dual use research of concern
☒	☐ Plants

Methods

n/a	Involved in the study
☒	☐ ChIP-seq
☒	☐ Flow cytometry
☒	☐ MRI-based neuroimaging

Plants

Seed stocks	Not relevant
Novel plant genotypes	Not relevant
Authentication	Not relevant
